# Exploring the Roles of *CREBRF* and *TRIM2* in the Regulation of Angiogenesis by High-Density Lipoproteins

**DOI:** 10.3390/ijms19071903

**Published:** 2018-06-28

**Authors:** Nathan K. P. Wong, Helena Cheung, Emma L. Solly, Laura Z. Vanags, William Ritchie, Stephen J. Nicholls, Martin K. C. Ng, Christina A. Bursill, Joanne T. M. Tan

**Affiliations:** 1 Immunobiology Research Group, Heart Research Institute, 7 Eliza Street, Newtown, NSW 2042, Australia; nathan.wong@sahmri.com (N.K.P.W.); chelenacheung@gmail.com (H.C.); laura.mcinnes3@gmail.com (L.Z.V.); christina.bursill@sahmri.com (C.A.B.); 2 Discipline of Medicine, The University of Sydney School of Medicine, Camperdown, NSW 2006, Australia; william.ritchie@igh.cnrs.fr (W.R.); mkcng@med.usyd.edu.au (M.K.C.N.); 3 Heart Health Theme, South Australian Health and Medical Research Institute, North Terrace, Adelaide, SA 5000, Australia; emma.solly@sahmri.com (E.L.S.); stephen.nicholls@sahmri.com (S.J.N.); 4 Centenary Institute, Royal Prince Alfred Hospital, Camperdown, NSW 2050, Australia; 5 Institute of Human Genetics–CNRS, 141 rue de la Cardonille, 34396 Montpellier CEDEX 5, France; 6 Adelaide Medical School, Faculty of Health & Medical Sciences, University of Adelaide, Adelaide, SA 5000, Australia; 7 Department of Cardiology, Royal Prince Alfred Hospital, Camperdown, NSW 2050, Australia

**Keywords:** angiogenesis, high-density lipoproteins, hypoxia, inflammation, *TRIM2*, *CREBRF*

## Abstract

Angiogenesis, the process of forming new blood vessels, is crucial in the physiological response to ischemia, though it can be detrimental as part of inflammation and tumorigenesis. We have previously shown that high-density lipoproteins (HDL) modulate angiogenesis in a context-specific manner via distinct classical signalling pathways, enhancing hypoxia-induced angiogenesis while suppressing inflammatory-driven angiogenesis. Whether additional novel targets exist to account for these effects are unknown. A microarray approach identified two novel genes, cyclic-adenosine-monophosphate-response-element-binding protein 3 regulatory factor (*CREBRF*) and tripartite motif-containing protein 2 (*TRIM2*) that were upregulated by reconstituted HDL (rHDL). We measured *CREBRF* and *TRIM2* expression in human coronary artery endothelial cells following incubation with rHDL and exposure to either hypoxia or an inflammatory stimulus. We found that *CREBRF* and *TRIM2* mRNA were significantly upregulated by rHDL, particularly in response to its phospholipid component 1-palmitoyl-2-linoleoyl-phosphatidylcholine, however, protein expression was not significantly altered. Knockdown of *TRIM2* impaired endothelial cell tubulogenesis in vitro in both hypoxia and inflammation, implying a necessary role in angiogenesis. Furthermore, *TRIM2* knockdown attenuated rHDL-induced tubule formation in hypoxia, suggesting that it is important in mediating the pro-angiogenic action of rHDL. Our study has implications for understanding the regulation of angiogenesis in both of these pathophysiological contexts by HDL.

## 1. Introduction

Angiogenesis, the process in which new blood vessels are formed from pre-existing vessels, is vital in many physiological contexts, such as during wound healing and as an adaptive response to tissue ischemia [1]. Angiogenesis, however, also plays a key role in many pathological conditions. Excessive angiogenesis stimulated by inflammation can accelerate the development of atherosclerotic plaque by providing additional conduits for the delivery of inflammatory cells and cytokines [2]. Dysregulated angiogenesis can also promote the growth, survival and metastasis of tumors [3,4]. Angiogenesis-associated pathologies such as cardiovascular disease and cancer contribute significantly to mortality and morbidity rates worldwide [5,6]. Any agent capable of differentially modulating angiogenesis in a context-specific manner would conceivably be of great therapeutic value.

In addition to its well-recognized athero-protective, anti-inflammatory, anti-oxidative and anti-thrombotic properties [7], a growing body of evidence suggests that high-density lipoproteins (HDL) are able to regulate angiogenesis in a multifunctional manner depending on the pathophysiological context [8]. Treatment of human coronary artery endothelial cells (HCAECs) with reconstituted HDL (rHDL), composed of the main apolipoprotein A-I (apoA-I) constituent of native HDL, complexed with the phospholipid 1-palmitoyl-2-linoleoyl-phosphatidylcholine (PLPC), significantly increased tubulogenesis, proliferation and migration of HCAECs in response to hypoxia [9,10]. These effects were attenuated when rHDL-treated HCAECs were exposed to the inflammatory cytokine, tumor necrosis factor α (TNFα) [9], suggesting a dichotomous role for HDL in angiogenesis. In an in vivo setting, intravenous delivery of rHDL or apoA-I in murine models of hindlimb ischemia (HLI) resulted in accelerated recovery of blood perfusion and increased capillary density in the ischemic hindlimbs [9,11,12,13]. Similar results were seen in a rabbit model of HLI following treatment with des-fluoro-anacetrapib, an inhibitor of cholesteryl ester transfer protein (CETP) which raises HDL levels [14]. Conversely, intravenous apoA-I impaired the inflammatory response that was induced by a silicon cuff placed around the femoral arteries of mice, reducing the formation of adventitial neovessels and the infiltration of macrophages [9]. Similarly, in a rabbit carotid cuff model, infusions of rHDL or apoA-I reduced the generation of vascular reactive oxygen species, leukocyte infiltration and expression of inflammatory chemokines [15]. These studies highlight that HDL is able to enhance hypoxia-induced angiogenesis while inhibiting inflammation-driven angiogenesis.

Many classically known angiogenic pathways and mediators have been implicated downstream of HDL. In hypoxia, HDL augments angiogenesis by interacting with its receptor scavenger receptor class B type I (SR-BI), activating the phosphatidylinositol-3-kinase (PI3K)/Akt pathway. This leads to modulation of the transcription factor hypoxia-inducible factor-1α (HIF-1α) and in turn upregulates the expression of vascular endothelial growth factor (VEGF) and chemokine CXCL12, both potent stimulators of angiogenesis [9,10]. Meanwhile in response to inflammation, HDL is able to inhibit the key transcription factor nuclear factor kappa B (NFκB) thereby reducing nucleic p65 (the active subunit of NFκB) and various mediators such as TNFα, monocyte chemoattractant protein-1 (MCP-1), vascular cell adhesion molecule-1 (VCAM-1) and intercellular adhesion molecule 1 (ICAM-1) which crucially recruit macrophages to sustain the inflammatory response [16,17,18]. As opposed to hypoxia, the expression of HIF-1α, VEGF and its receptor, VEGF receptor 2 (VEGFR2), are inhibited by HDL in inflammation [9], leading to suppression of angiogenesis where it can be detrimental.

Whether HDL’s effects on angiogenesis are mediated through novel and yet uncharacterized molecular pathways remains unknown, but it is clear that further elucidation of HDL’s mechanistic targets is needed to facilitate potential clinical translation of HDL-related therapies in diseases associated with angiogenesis. In this study, we aimed to explore the possibility of novel genes that could be involved in the regulation of angiogenesis by HDL under both hypoxic and inflammatory conditions. We undertook a microarray approach and identified, for the first time, two genes cyclic-adenosine-monophosphate-response-element-binding protein 3 regulatory factor (*CREBRF*) and tripartite motif-containing protein 2 (*TRIM2*), that were upregulated by rHDL under both angiogenic conditions. We further investigated the expression profiles of these genes in HCAECs as triggered by rHDL and its individual components apoA-I and PLPC, showing the latter to be particularly important in promoting the transcription of both genes. Moreover, we demonstrate that knockdown of *TRIM2* impairs tubulogenesis in vitro, highlighting a putative role for this gene in angiogenesis, and more broadly in vascular biology, that has not been previously characterized. Finally, *TRIM2* knockdown attenuated the ability of rHDL to augment tubule formation in hypoxia, suggesting that it is important in mediating the pro-angiogenic action of rHDL in physiological conditions.

## 2. Results

### 2.1. CREBRF and TRIM2 Are Novel Targets of rHDL in Response to Angiogenic Stimuli

**Figure 1:**
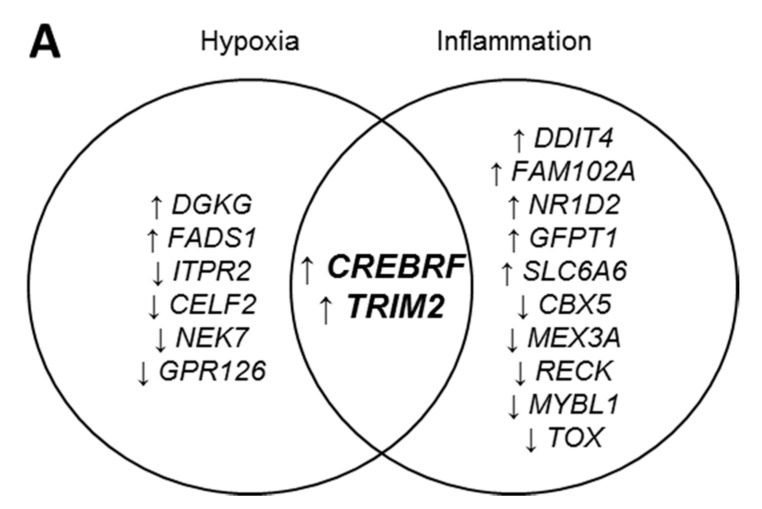

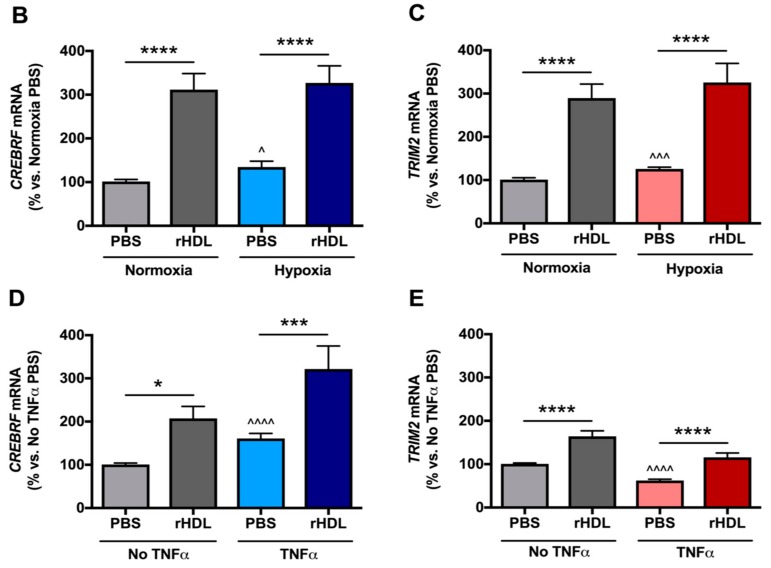
*CREBRF* and *TRIM2* mRNA expression in human coronary artery endothelial cells (HCAECs) in response to treatment with reconstituted high-density lipoproteins (rHDL) or phosphate-buffered saline (PBS, vehicle control) in hypoxia and inflammation. (**A**) A microarray approach identified genes whose expression was increased (↑) or decreased (↓) in response to rHDL in hypoxia and inflammation. (**B**) *CREBRF* mRNA and (**C**) *TRIM2* mRNA in PBS- and rHDL-treated cells exposed to normoxia and hypoxia; (**D**) *CREBRF* mRNA and (**E**) *TRIM2* mRNA in PBS- and rHDL-treated cells with and without tumor necrosis factor alpha (TNFα) stimulation. mRNA expression was measured by real-time quantitative polymerase chain reaction (qPCR). Results are mean ± SEM. * *p* < 0.05, *** *p* < 0.001, **** *p* < 0.0001 by two-way ANOVA with post hoc analysis using Bonferroni’s multiple comparisons test. ^ *p* < 0.05, ^^^ *p* < 0.001, ^^^^ *p* < 0.0001 by unpaired two-tailed *t*-test.

A microarray study using human microvascular endothelial cells (HMVECs) was undertaken to investigate potential gene targets of rHDL in hypoxia and inflammation. Over 20,000 genes mapped through RefSeq or via UniGene annotation were screened using the GeneChip^®^ PrimeView^TM^ Human Gene Expression Arrays. In response to hypoxia stimulation, rHDL significantly regulated 1452 genes (Supplementary Table S1) while 1256 genes were differentially regulated by rHDL following an inflammatory stimulus (Supplementary Table S2). Further analysis was then undertaken to identify the top differentially expressed genes with no established role in angiogenesis that were specifically regulated by rHDL in response to angiogenic stimuli. Eight genes demonstrated significant changes in expression when treated with rHDL under hypoxic conditions (Figure 1A). Four genes were significantly upregulated (*CREBRF*, *TRIM2*, *DGKG* and *FADS1*) while four were downregulated (*CELF2*, *GPR126*, *NEK7* and *ITPR2*). Conversely, twelve genes exhibited significant changes in expression when HMVECs were treated with rHDL then exposed to the inflammatory cytokine TNFα. Seven genes were upregulated (*CREBRF*, *TRIM2*, *FAM102A*, *GFTP1*, *DDIT4*, *NR1D2* and *SLC6A6*) while five were downregulated (*TOX*, *MEX3A*, *CBX5*, *MYBL1* and *RECK*). Two genes, *CREBRF* and *TRIM2*, were significantly upregulated by rHDL in response to both hypoxia and inflammation.

### 2.2. rHDL Modulates CREBRF and TRIM2 Expression in Human Coronary Artery Endothelial Cells (HCAECs) under Hypoxia and Inflammation

HCAECs pre-treated with rHDL under normal oxygen conditions showed significant increases in *CREBRF* (307 ± 36%, Figure 1B) and *TRIM2* (286 ± 32%, Figure 1C) mRNA levels compared to phosphate-buffered saline (PBS) controls (both *p* < 0.0001). Under hypoxic conditions, there was also increased expression of *CREBRF* (243 ± 29%) and *TRIM2* (259 ± 35%) following treatment with rHDL (both *p* < 0.0001 vs. hypoxia PBS controls). Meanwhile, HCAECs treated with PBS alone had significantly upregulated *CREBRF* (132 ± 13%) and *TRIM2* (124 ± 4%) mRNA levels in hypoxia compared to normoxia (*p* < 0.05 and *p* < 0.001, respectively). This suggested that rHDL was able to augment the hypoxia-driven increase in mRNA expression of both *CREBRF* and *TRIM2*. In the absence of an inflammatory stimulus, rHDL also led to upregulation of *CREBRF* (206 ± 27%, Figure 1D) and *TRIM2* (163 ± 12%, Figure 1E) mRNA levels relative to PBS controls (*p* < 0.05 and *p* < 0.0001, respectively). Subsequent exposure of HCAECs to TNFα induced a significant increase in *CREBRF* mRNA (160 ± 12%) but interestingly, a significant reduction in *TRIM2* mRNA (62 ± 3%). rHDL pre-treatment of TNFα-stimulated HCAECs further increased expression of *CREBRF* (200 ± 33%) and was able to rescue the drop in *TRIM2* mRNA levels (175 ± 29%) that occurred (*p* < 0.001 and *p* < 0.0001, respectively vs. TNFα PBS control). These findings collectively indicate that rHDL was able to induce both *CREBRF* and *TRIM2* mRNA expression under basal conditions, however, these effects were further modulated when cells were exposed to hypoxia and inflammatory stimuli.

**Figure 2:**
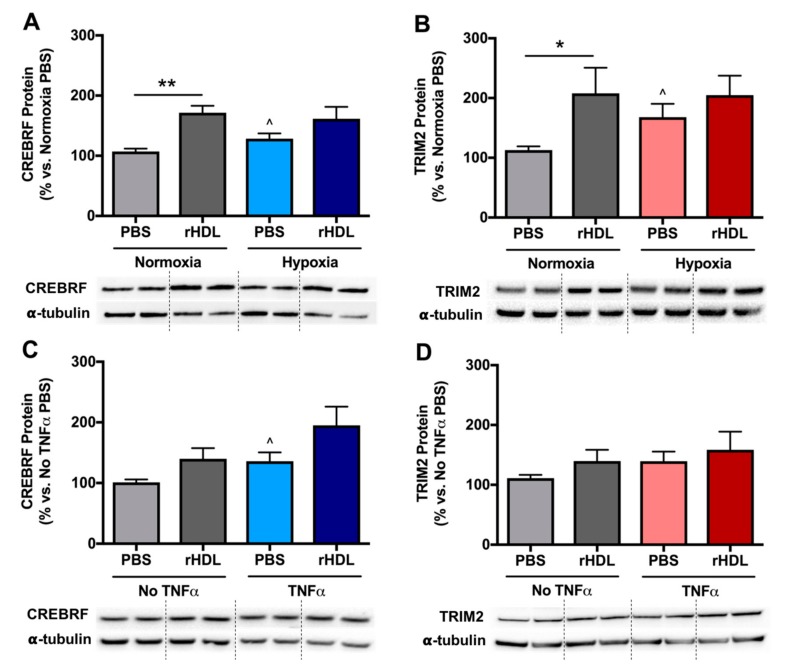
CREBRF and TRIM2 protein expression in HCAECs in response to rHDL or PBS treatment in hypoxia and inflammation, with representative Western blot images. (**A**) CREBRF protein and (**B**) TRIM2 protein in PBS- and rHDL-treated cells exposed to normoxia and hypoxia; (**C**) CREBRF protein and (**D**) TRIM2 protein in PBS- and rHDL-treated cells with and without tumor necrosis factor alpha (TNFα) stimulation. Results are mean ± SEM. * *p* < 0.05, ** *p* < 0.01 by two-way ANOVA with post hoc analysis using Bonferroni’s multiple comparisons test. ^ *p* < 0.05 by unpaired two-tailed *t*-test.

Consistent with changes seen with mRNA expression, HCAECs exposed to hypoxic conditions alone exhibited significantly higher CREBRF (120 ± 8%; Figure 2A) and TRIM2 (149 ± 20%, Figure 2B) protein levels compared to normoxia controls (both *p* < 0.05). In normoxia, pre-treatment with rHDL significantly increased CREBRF protein (160 ± 11%) and TRIM2 protein (184 ± 38%) compared to pre-treatment with PBS alone (*p* < 0.01 and *p* < 0.05 respectively), reflecting a similar trend seen with mRNA expression. However, the rise in CREBRF protein (126 ± 15%) and TRIM2 protein (122 ± 19%) following rHDL treatment in hypoxia was not statistically significant.

HCAECs stimulated with TNFα had higher CREBRF (134 ± 14%, Figure 2C) and TRIM2 (125 ± 15%, Figure 2D) protein levels compared to PBS controls, however only the former was statistically significant (*p* < 0.05). In the absence of TNFα, pre-treatment with rHDL increased CREBRF and TRIM2 protein (138 ± 17% and 126 ± 17%, respectively vs. PBS controls), though this did not reach statistical significance. Following exposure to TNFα, CREBRF and TRIM2 protein levels were increased in response to rHDL treatment (144 ± 23% and 114 ± 22%, respectively vs. TNFα PBS controls), however, again this was not significant. These findings collectively imply that rHDL has more striking effects on *CREBRF* and *TRIM2* expression at the transcriptional rather than the translational level.

### 2.3. rHDL Upregulates CREBRF and TRIM2 mRNA Expression in HCAECs Predominantly through PLPC

**Figure 3:**
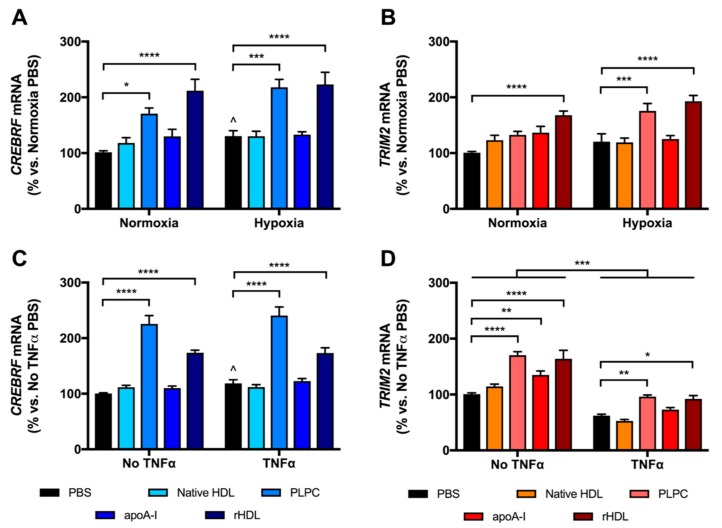
*CREBRF* and *TRIM2* mRNA expression in HCAECs following treatment with native HDL, 1-palmitoyl-2-linoleoyl-phosphatidylcholine (PLPC), lipid-free apolipoprotein A-I (apoA-I), rHDL or PBS in hypoxia and inflammation. (**A**) *CREBRF* mRNA and (**B**) *TRIM2* mRNA in cells exposed to normoxia and hypoxia; (**C**) *CREBRF* mRNA and (**D**) *TRIM2* mRNA in cells with and without TNFα stimulation. mRNA expression was measured by real-time quantitative polymerase chain reaction (qPCR). Results are mean ± SEM. * *p* < 0.05, ** *p* < 0.01, *** *p* < 0.001, **** *p* < 0.0001 by two-way ANOVA with post hoc analysis using Bonferroni’s multiple comparisons test.

To determine the forms and components of HDL that may be responsible for the effects on *CREBRF* and *TRIM2* mRNA expression, HCAECs were treated separately with native HDL, rHDL and its individual components, apoA-I and PLPC. Consistent with previous findings, treatment with rHDL significantly increased *CREBRF* mRNA expression in normoxia and hypoxia (209 ± 20% and 171 ± 17% vs. respective PBS controls, both *p* < 0.0001, Figure 3A), as well as increasing *TRIM2* mRNA in normoxia and hypoxia (167 ± 8% and 160 ± 9% vs. respective PBS controls, both *p* < 0.0001, Figure 3B). This effect was largely attributable to the PLPC component of rHDL, as treatment with PLPC alone significantly increased *CREBRF* mRNA expression in normoxia and hypoxia (168 ± 10% and 167 ± 11% vs. PBS controls, *p* < 0.05 and *p* < 0.0001, respectively), as well as increasing *TRIM2* mRNA in hypoxia (146 ± 11% vs. PBS control, *p* < 0.001). There were no significant differences in *CREBRF* or *TRIM2* mRNA following treatment with native HDL or apoA-I in normoxia or hypoxia.

Treatment of HCAECs with rHDL increased *CREBRF* mRNA expression with and without TNFα (146 ± 8% and 173 ± 5% vs. respective PBS controls, both *p* < 0.0001, Figure 3C), again consistent with previous findings. Strikingly however, treatment with the PLPC component of rHDL alone resulted in increased *CREBRF* mRNA expression beyond that induced by rHDL, both in the presence and absence of TNFα (203 ± 13% and 225 ± 15% vs. respective PBS controls, both *p* < 0.0001). Native HDL or apoA-I treatment did not alter *CREBRF* mRNA significantly. Meanwhile, *TRIM2* mRNA was significantly increased in HCAECs in response to PLPC, apoA-I and rHDL (189 ± 21%, 134 ± 7% and 163 ± 15% vs. PBS control with *p* < 0.0001, *p* < 0.01 and *p* < 0.0001, respectively, Figure 3D). In response to TNFα, a significant global decrease in *TRIM2* mRNA was seen (*p* < 0.001). However, only PLPC and rHDL treatment rescued this drop in *TRIM2* mRNA (154 ± 5% and 148 ± 10% vs. TNFα PBS control with *p* < 0.01 and *p* < 0.05, respectively). Collectively, these findings imply that rHDL modulates *CREBRF* and *TRIM2* mRNA expression predominantly via PLPC.

### 2.4. Tubulogenesis is Impaired by Lentiviral Knockdown of TRIM2 in Hypoxia and Inflammation

*CREBRF* and *TRIM2* expression was suppressed by treating HCAECs with short hairpin RNA (shRNA) delivered using a lentiviral approach. shRNA-mediated knockdown of CREBRF and TRIM2 protein expression was confirmed by Western blotting (Supplementary Figure S1).

**Figure 4:**
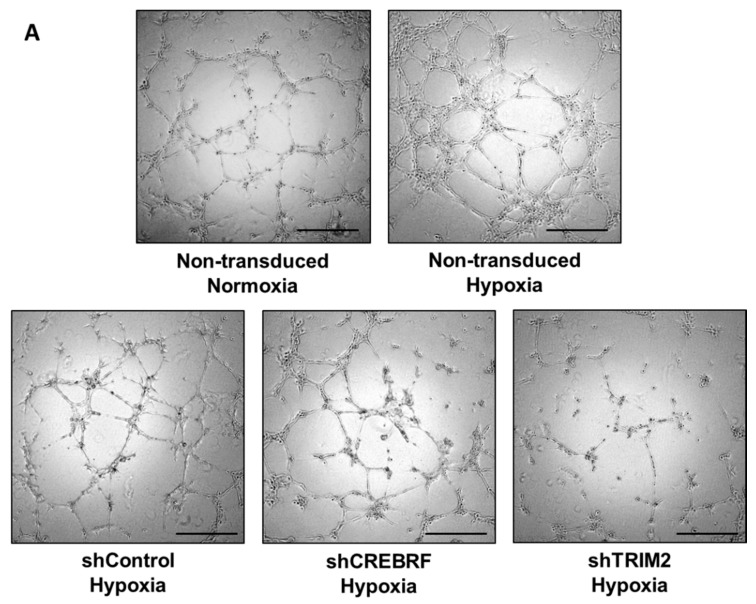

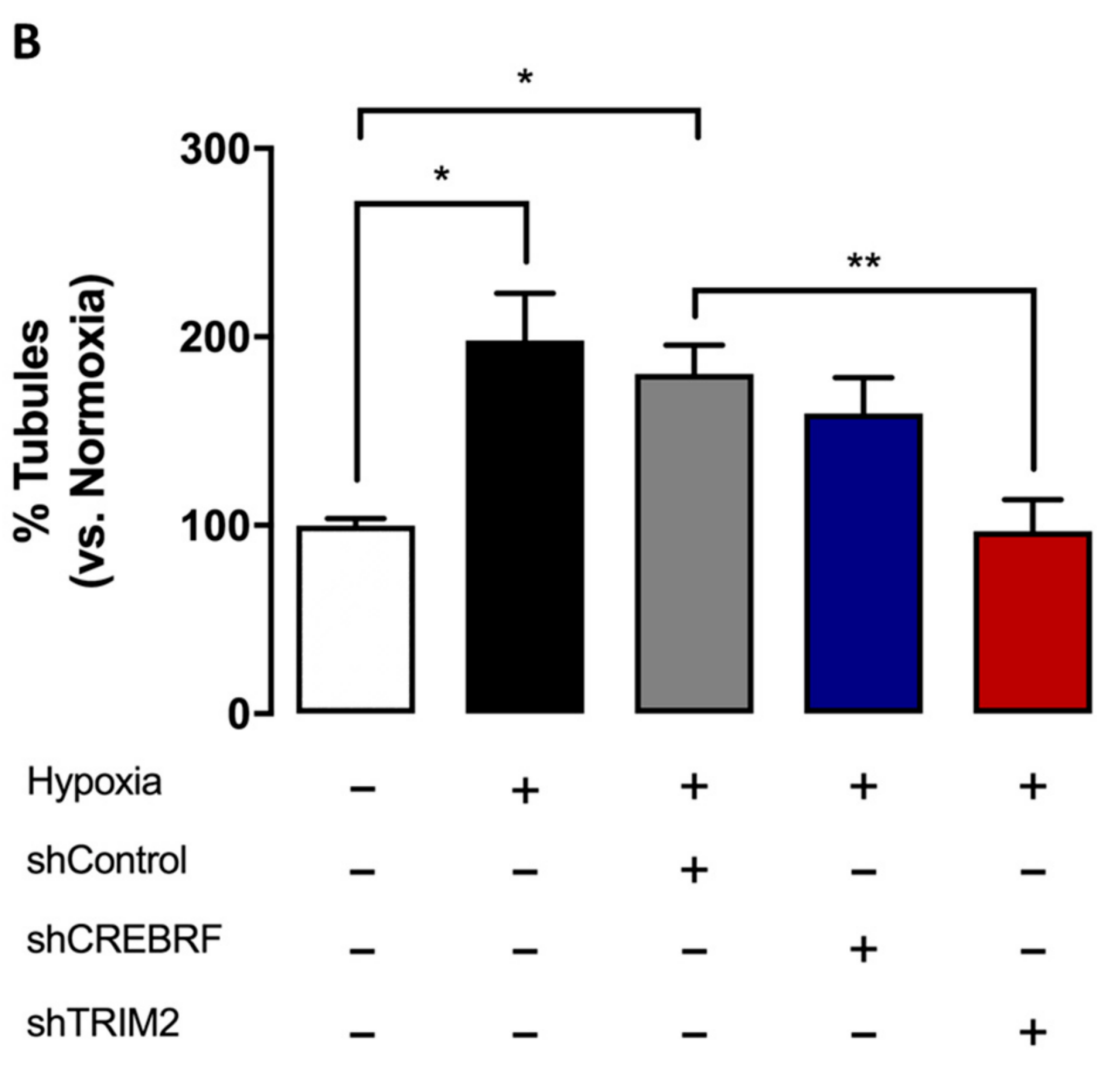
Tubulogenesis under hypoxic conditions in HCAECs transduced with lentivirus containing shRNA against a control sequence (shControl), *CREBRF* (shCREBRF) or *TRIM2* (shTRIM2) compared to non-transduced HCAECs under normoxic conditions. (**A**) Representative images of tubule formation were photographed under light microscopy. Scale bars, 50 μm; (**B**) Number of tubules was counted and analyzed using ImageJ software. Results are mean ± SEM. * *p* < 0.05, ** *p* < 0.01 by unpaired two-tailed *t*-test.

Consistent with our previous studies [9,10], we found that hypoxia induces endothelial tubule formation when compared to the normoxia control (*p* < 0.05, Figure 4). Under hypoxic conditions, the extent of tubulogenesis in HCAECs transduced with lentivirus containing shRNA against *TRIM2* was significantly reduced (54 ± 9%) compared to cells transduced with shRNA against a random control sequence (shControl, *p* < 0.01). HCAECs transduced with lentivirus containing shRNA against *CREBRF* also demonstrated reduced tubulogenesis (88 ± 11%) relative to shControl cells, however this was not statistically significant.

**Figure 5:**
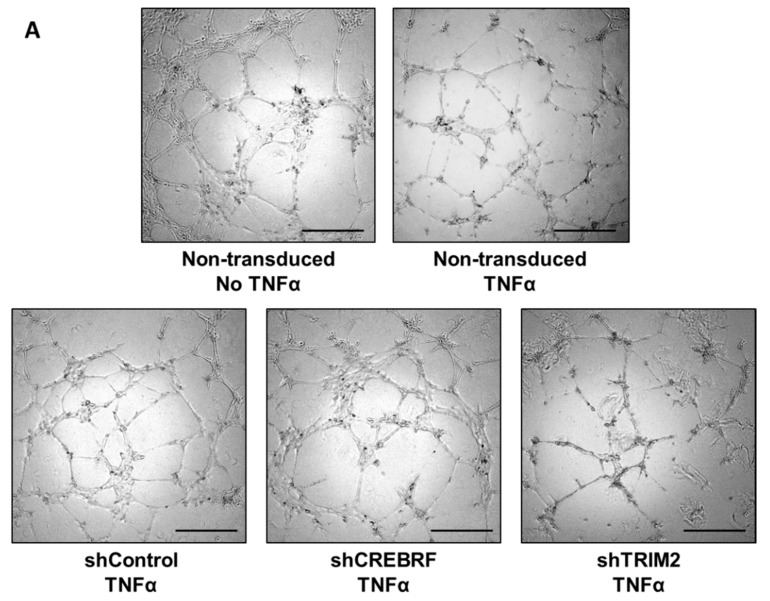

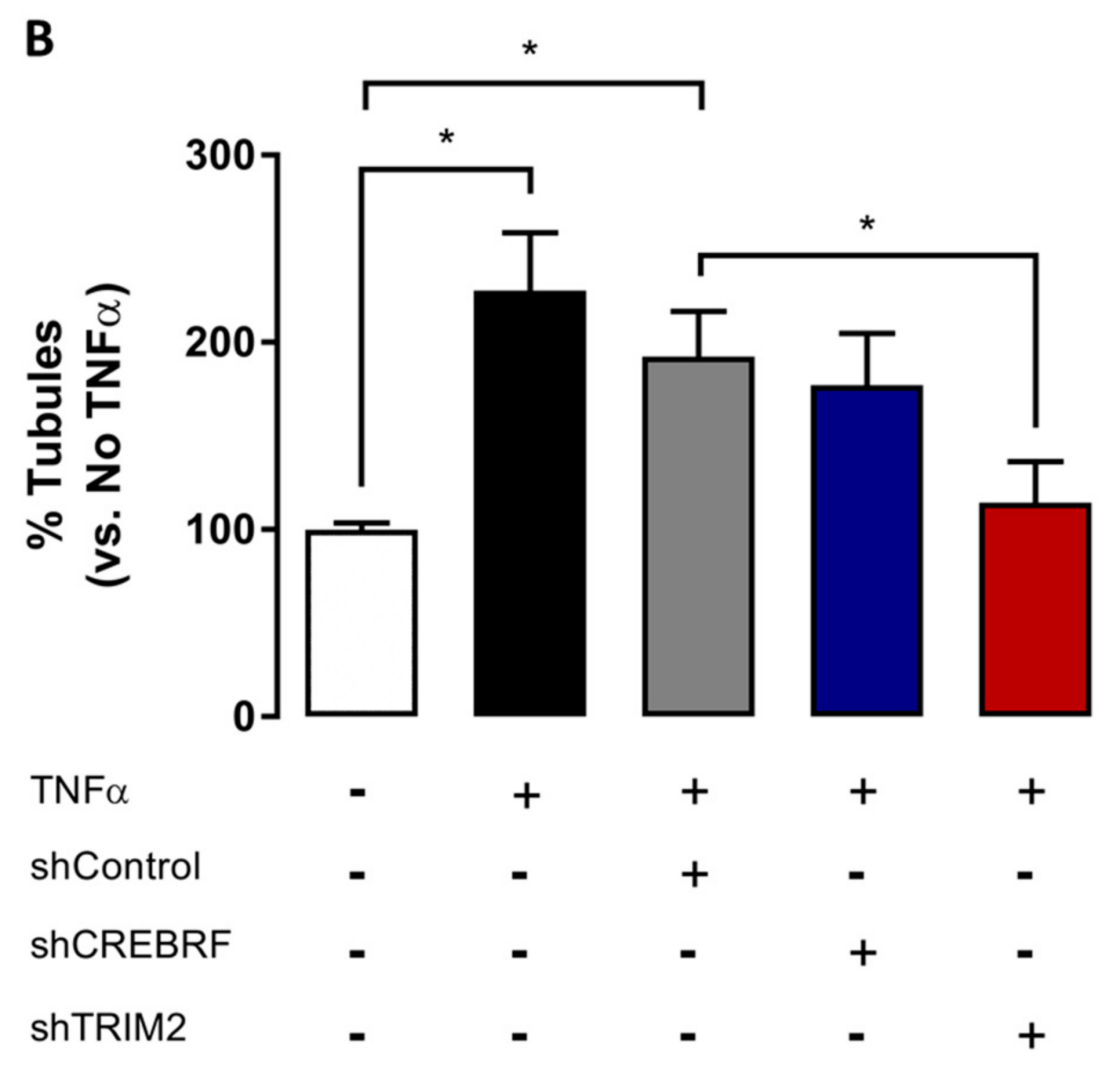
Tubulogenesis following TNFα stimulation in HCAECs transduced with lentivirus containing shControl, shCREBRF or shTRIM2 compared to non-transduced non-TNFα-stimulated cells. (**A**) Representative images of tubule formation were photographed under light microscopy. Scale bars, 50 μm. (**B**) Number of tubules was counted and analyzed using ImageJ software. Results are mean ± SEM. * *p* < 0.05 by unpaired two-tailed *t*-test.

Stimulation with the inflammatory cytokine TNFα promoted tubule formation compared to non-TNFα-stimulated cells (*p* < 0.05, Figure 5). Interestingly, TNFα-stimulated tubule formation was significantly reduced in HCAECs transduced with lentivirus containing shRNA against *TRIM2* (59 ± 11%) compared to cells transduced with shControl (*p* < 0.05). HCAECs transduced with lentivirus containing shRNA against *CREBRF* demonstrated marginally reduced tubulogenesis (92 ± 14%) relative to shControl cells but this was not statistically significant.

### 2.5. Lentiviral Knockdown of TRIM2 Attenuates the Pro-Angiogenic Action of rHDL in Hypoxia

**Figure 6:**
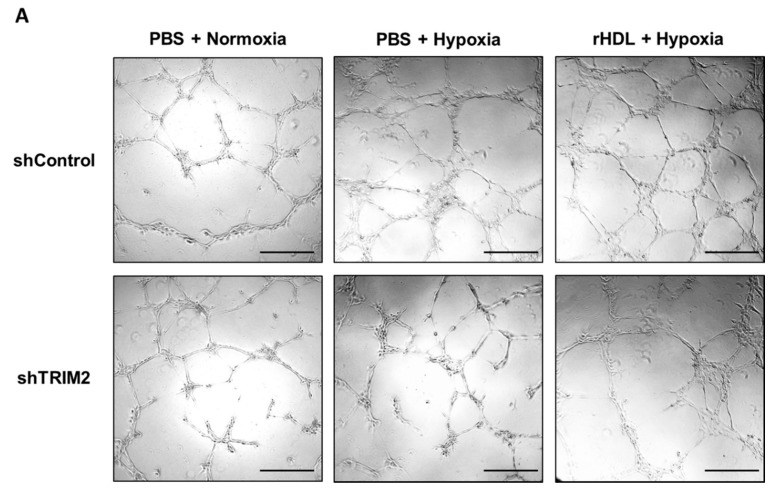

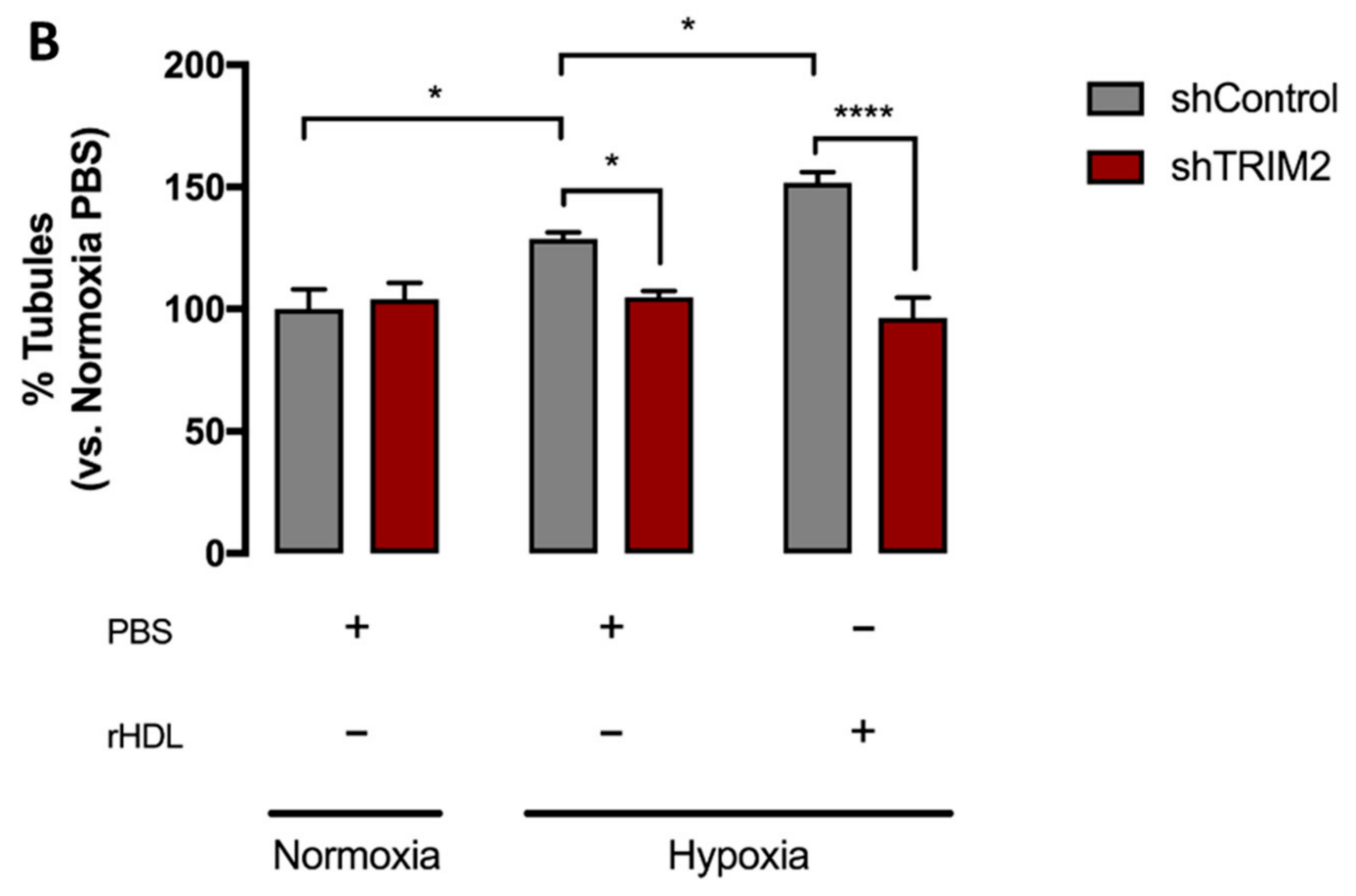
Tubulogenesis following exposure to hypoxia in HCAECs transduced with lentivirus containing shControl or shTRIM2 treated with either PBS or rHDL compared to cells under normoxic conditions. (**A**) Representative images of tubule formation were photographed under light microscopy. Scale bars, 50 μm; (**B**) Number of tubules was counted and analyzed using ImageJ software. Results are mean ± SEM. * *p* < 0.05, **** *p* < 0.0001 by two-way ANOVA with post hoc analysis using Bonferroni’s multiple comparisons test.

**Figure 7:**
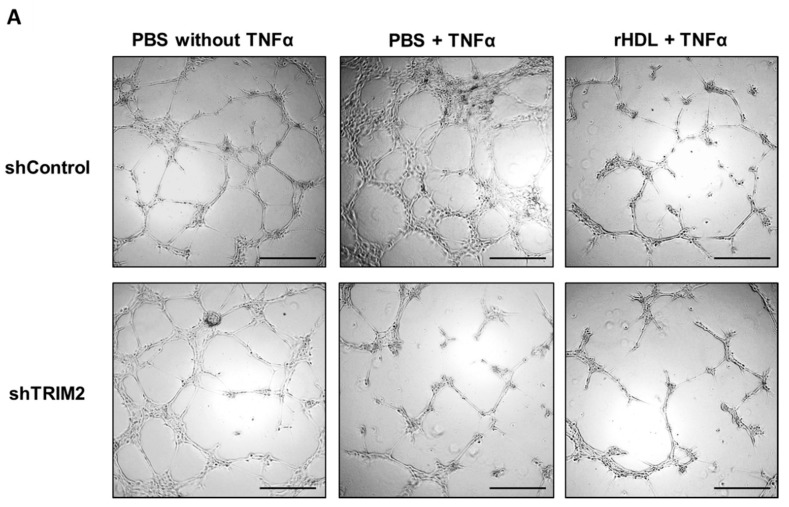

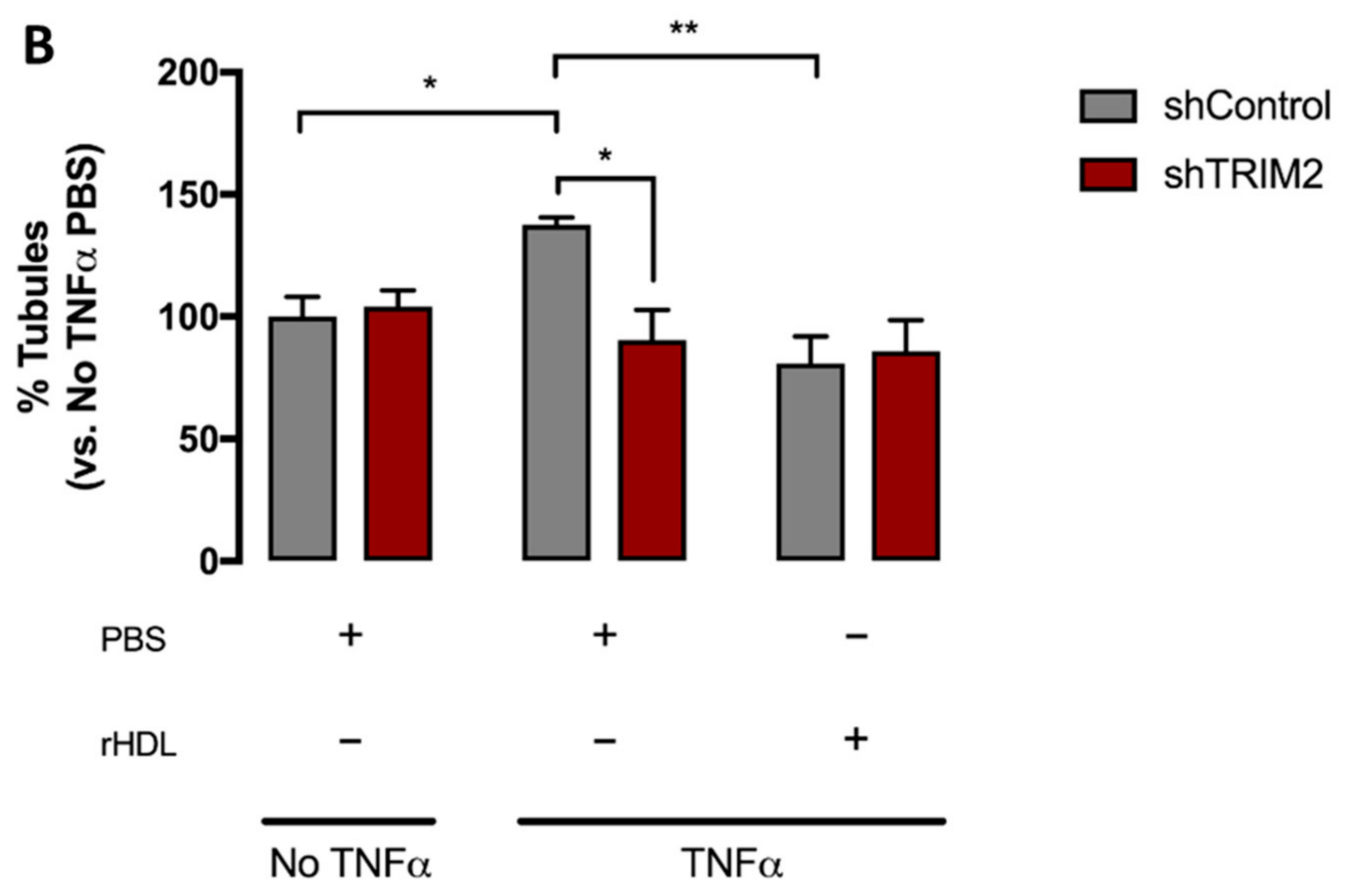
Tubulogenesis following TNFα stimulation in HCAECs transduced with lentivirus containing shControl or shTRIM2 treated with either PBS or rHDL compared to non-stimulated cells. (**A**) Representative images of tubule formation were photographed under light microscopy. Scale bars, 50 μm; (**B**) Number of tubules was counted and analyzed using ImageJ software. Results are mean ± SEM. * *p* < 0.05, ** *p* < 0.01 by two-way ANOVA with post hoc analysis using Bonferroni’s multiple comparisons test.

We next sought to determine whether TRIM2 is important in mediating the angiogenic action of rHDL. Cells transduced with either shControl or shTRIM2 were treated with PBS or rHDL, then underwent a Matrigel tubulogenesis assay. We found that rHDL significantly augmented hypoxia-induced tubule formation in shControl cells (*p* < 0.05, Figure 6). However, the pro-angiogenic action of rHDL in hypoxia was attenuated in shTRIM2 cells (*p* < 0.0001). On the other hand, treatment with rHDL suppressed TNFα-stimulated tubule formation (*p* < 0.05, Figure 7), however, interestingly, TRIM2 knockdown did not inhibit this effect any further.

## 3. Discussion

The finding that HDLs are able to promote hypoxia-induced physiological angiogenesis yet inhibit inflammation-driven pathological angiogenesis represents an exciting therapeutic prospect for a range of angiogenesis-associated diseases. Our current study employed a microarray approach to screen for novel genes that may regulate angiogenesis downstream of HDL. We identified *CREBRF* and *TRIM2* as being upregulated by rHDL under both hypoxia and TNFα-induced inflammation. This finding was supported by measuring mRNA and protein expression of these genes in HCAECs, though interestingly, the observed changes were much more striking at a transcriptional rather than translational level. Moreover, the effects appeared to be predominantly attributable to the PLPC component of rHDL. Knockdown of *TRIM2* was found to impair tubulogenesis in vitro particularly under hypoxic conditions, indicating its potential role in physiological angiogenesis.

To date, relatively little is known about the function of *TRIM2* and its role in cardiovascular biology has not yet been previously explored. *TRIM2* encodes the tripartite motif-containing protein 2, an E3 ubiquitin ligase highly expressed in the central nervous system that functions to ubiquitinate and target certain proteins for proteasomal degradation [19]. In particular, *TRIM2* is known to target neurofilament light-chain (NF-L), and in doing so, directs neuronal polarization by allowing the specification of particular neurites to become axons [20]. In a *TRIM2*-knockout mouse model, intra-axonal accumulation of NF-L secondary to TRIM2 deficiency caused progressive neurodegeneration that manifested as juvenile-onset tremor and ataxia [19]. Human case reports have also described loss-of-function mutations in *TRIM2* causing early-onset axonal neuropathies [21,22]. Interestingly, *TRIM2* has been found to confer neuroprotection following brief periods of ischemia by targeting the pro-apoptotic factor Bim for degradation [23]. It is conceivable that *TRIM2* may well play a similar role in mediating the angiogenic response to ischemia downstream of HDL.

By comparison, even less is known about *CREBRF*, which encodes a regulatory factor for cyclic adenosine monophosphate (cAMP)-response element binding protein 3 (CREB3). *CREBRF* is highly expressed in mouse heart and kidney, and functions as a negative regulator of CREB3, a transcription factor activated as part of the endoplasmic reticulum (ER) unfolded protein response (UPR) [24]. Under conditions of prolonged ER stress, the ER membrane-bound protein CREB3 is cleaved and translocates to the nucleus where it activates UPR-related target genes. When present, CREBRF binds and sequesters CREB3 into discrete nuclear sub-regions, suppressing its activity and promoting its degradation [24]. How this mechanism may be linked to angiogenesis or HDL is unclear, however the role of ER stress and UPR activation in tumor-related angiogenesis [25,26] and endothelial dysfunction [27,28] is increasingly being recognized. HDL has been shown to directly inhibit ER stress induced by oxidized low-density lipoproteins which promote the formation of macrophage-derived foam cells in atherosclerotic plaque [29,30].

In our current study, there was marked upregulation of both *CREBRF* and *TRIM2* mRNA secondary to rHDL treatment in HCAECs, validating the findings of the microarray study. This effect was more related to rHDL itself rather than hypoxia or TNFα stimulation. Interestingly, we observed that *TRIM2* mRNA was reduced in response to TNFα while it was increased in hypoxia independent of HDL treatment. rHDL treatment subsequently reversed the TNFα-induced drop in *TRIM2* mRNA, while augmenting the rise in *TRIM2* mRNA in hypoxia. These dichotomous effects may reflect a role for *TRIM2* as a molecular switch that directs HDL’s actions down distinct angiogenic pathways in a context-specific manner, though more studies will be required to clarify this effect. It is worth nothing that native HDLs did not significantly alter mRNA expression of *CREBRF* or *TRIM2* in HCAECs when compared to PBS, as opposed to the effect of rHDL. This finding is consistent with previous studies of angiogenesis in both hypoxia and inflammation [9], and likely reflects the heterogeneity of native HDL particles that have been isolated from multiple human volunteers. These HDL particles may have also undergone certain chemical and/or compositional modifications in vivo that have rendered them somewhat dysfunctional. On the other hand, rHDL is synthesized de novo with purified apoA-I and PLPC; and should therefore have minimal loss of function.

Moreover, by isolating rHDL into its components, our study also demonstrated that PLPC, as opposed to apoA-I, appeared to be primarily driving the transcriptional upregulation of *CREBRF* and *TRIM2*. Few studies have directly explored which components of HDL may be responsible for its regulation of angiogenesis. Several studies have shown that apoA-I can modulate physiological and pathological angiogenesis in vivo [9,31,32] but the effect of phospholipids alone and whether these activate alternative signalling pathways to apoA-I is unknown. Prosser et al. demonstrated that rHDL significantly enhances hypoxia-induced tubule formation in HCAECs and increases the expression of angiogenic mediators HIF-1α and VEGF, but these effects were not seen with lipid-free apoA-I (i.e., without PLPC), suggesting a potential role for PLPC [9]. Earlier studies also found that phospholipid composition significantly alters the ability of rHDL to inhibit TNFα-induced VCAM-1 expression, finding PLPC was particularly potent in its anti-inflammatory effect compared to other phospholipid species [33]. In our study, the ability of PLPC vesicles to induce *CREBRF* mRNA expression beyond that of rHDL under inflammatory conditions was surprising. This was, however, an isolated finding not seen under hypoxic conditions or with TRIM2 mRNA expression. Moreover, previous studies by our group have found that PLPC vesicles alone do not augment tubulogenesis in HCAECs [10], implying that while it is interesting that PLPC upregulated *CREBRF* and *TRIM2* mRNA expression, this may not translate to a functional angiogenic response.

Though we observed marked changes in *CREBRF* and *TRIM2* mRNA in response to rHDL, these changes were generally not translated to significant changes in protein expression. This may reflect the presence of additional aspects of post-transcriptional regulation that have yet to be characterized. One example is the finding that *TRIM2* is a target of non-coding microRNA-181c [34], which has been implicated in cancer signalling [35,36,37]. A recent in vivo study also demonstrated that microRNA-181c, through its modulation of *TRIM2*, can attenuate cognitive impairment in rats induced by chronic cerebral ischemia [38]. In the case of *CREBRF*, the rapid turnover of CREBRF protein, with a reported half-life of less than 20 min [24], may also account for the lack of changes seen at the protein level. Further studies will be needed to elucidate whether microRNAs or other post-transcriptional or post-translational mechanisms significantly alter the expression of these genes, and whether changes in mRNA levels alone are sufficient to affect cellular signalling and functional angiogenesis.

In line with this, we assessed the effect of *CREBRF* and *TRIM2* knockdown on angiogenesis in vitro using a well-validated tubulogenesis assay. shRNA knockdown of *CREBRF* did not produce any significant inhibition of tubulogenesis, suggesting that *CREBRF* may not play a critical role in angiogenesis despite being transcriptionally upregulated in response to rHDL under both conditions. However, *TRIM2*-deficient HCAECs had significantly impaired tubulogenesis in both hypoxic and inflammatory conditions, suggesting that *TRIM2* is more important for angiogenic function. Furthermore, we find that knockdown of *TRIM2* attenuated the ability of rHDL to augment tubule formation in hypoxia. However, *TRIM2* knockdown did not have any further additional benefits to the anti-angiogenic action of rHDL under inflammatory conditions. This demonstrates that TRIM2 may be a novel regulator of the pro-angiogenic action of rHDL in hypoxia.

## 4. Materials and Methods

### 4.1. Preparation of Native HDL, Discoidal rHDL, ApoA-I, and Phospholipid Vesicles

Native HDLs were isolated from pooled samples of normal human plasma (5 donors, Red Cross Blood Service, Alexandria, NSW, Australia). The HDL fraction (1.063–1.210 g/mL) was isolated by ultracentrifugation followed by dialysis against phosphate-buffered saline. ApoA-I was further isolated from delipidated HDL by anion-exchange chromatography. rHDL was prepared from purified apoA-I complexed with PLPC using the cholate dialysis method at an initial PLPC:apoA-I molar ratio of 100:1. The final PLPC:apoA-I molar ratio was 80–100:1. PLPC vesicles were prepared by sonication (3 cycles of 5 min each on ice) in PBS and cholate then dialysed against PBS. The protein concentration of apoA-I was determined using the bicinchoninic acid (BCA) assay (ThermoFisher Scientific, Scoresby, VIC, Australia), and phospholipid concentrations were determined enzymatically (Wako, Richmond, VA, USA).

### 4.2. Microarray

GeneChip^®^ PrimeView^TM^ Human Gene Expression Arrays were purchased from Affymetrix, Inc. (Santa Clara, CA, USA). Human microvascular endothelial cells (HMVECs, Cell Applications, San Diego, CA, USA) were cultured and used at passage 5. They were seeded at a density of 8 × 10^4^ cells/well and 1.5 × 10^5^ cells/well for the hypoxia and inflammation experiments respectively, then pre-incubated for 24 h with either PBS or rHDL (20 µM, final apoA-I concentration). Cells were exposed to hypoxia (1.2% O_2_ balanced with N_2_) for 8 h or the inflammatory cytokine TNFα (0.6 ng/mL) for 4.5 h. Total RNA was extracted as outlined below. The GeneChip^®^ 3′ IVT PLUS Reagent Kit (Affymetrix, Inc., Santa Clara, CA, USA) was then used to prepare targets for the arrays. Poly-A RNA control stock was diluted 1:200,000 then added to 250 ng total RNA. This was reverse transcribed into first strand complementary DNA (cDNA) in an Eppendorf^®^ Mastercycler, followed by second strand cDNA synthesis. Biotin-labelled amplified RNA (aRNA) was generated by in vitro transcription following incubation for 16 h. aRNA was purified on a magnetic plate using aRNA Binding Mix containing magnetic beads, then quantified using a NanoDrop spectrophotometer. 12 μg aRNA was taken to undergo fragmentation, followed by quality assessment using a Bio-Rad Experion electrophoresis system. 10 μg of fragmented aRNA underwent hybridization to the array for 16.5 h in a GeneChip^®^ Hybridization Oven 645 (Affymetrix), followed by washing and staining on a GeneChip^®^ Fluidics Station 450 (Affymetrix). The arrays were scanned using a GeneChip^®^ Scanner 3000 7G (Affymetrix), then results were analyzed by the Bioinformatics Lab, Centenary Institute, NSW, Australia.

### 4.3. Cell Culture

Human coronary artery endothelial cells (Cell Applications, San Diego, CA, USA) were cultured in MesoEndo medium (Cell Applications) and used at passages 3–4. HCAECs were then trypsinized, counted and seeded at a density of 8 × 10^4^ cells/well and 1.5 × 10^5^ cells/well for the hypoxia and inflammation experiments, respectively, and cultured at 37 °C in 5% CO_2_. HCAECs were then pre-incubated for 24 h with either PBS (vehicle control) or rHDL comprised of apoA-I complexed with PLPC at a final apoA-I concentration of 20 µM. Following incubation, cells were washed with PBS and replaced with fresh MesoEndo media. The cells were then exposed to an angiogenic stimulus, either by incubation for 8 h at 5% CO_2_ and 1.2% O_2_ balanced with N_2_ (to mimic hypoxia), or by treatment with TNFα (0.6 ng/mL) for 4.5 h (to mimic inflammation). Each experiment was performed at least six times independently with duplicates or triplicates for each condition.

To investigate the effect of various components of HDL, HCAECs were cultured in MesoEndo media as described above. HCAECs were pre-incubated for 16 h with either PBS control or rHDL, apoA-I, native HDL (all 20 μM final apoA-I concentration) or PLPC vesicles (2 mM). The cells were then exposed to hypoxia or TNFα as described above. Each experiment was performed at least three times independently with duplicates or triplicates for each condition.

### 4.4. mRNA Expression

To measure mRNA expression, total RNA was extracted with TRI reagent and the amount quantitated using a NanoDrop spectrophotometer. Absorbance was measured at 260 and 280 nm, and the purity was determined from the absorbance ratio (*A*_260_/*A*_280_). 200 ng of total RNA was reverse transcribed in triplicate using the iScript cDNA synthesis kit (Bio-Rad Laboratories, Hercules, CA, USA). The expression of *TRIM2* and *CREBRF* was measured by real-time PCR using SYBR Green fluorophore in a Bio-Rad Cfx96 thermocycler. All amplicons were amplified using iQ SYBR Green Supermix (Bio-Rad Laboratories), and 20 pmol each of forward and reverse primers for *TRIM2* (F: 5′-ATCCGATCCGCTGATGTGTC-3′; R: 5′-TGTACATGCTTGCGGGTCTT-3′), *CREBRF* (F: 5′-GGAAGGTCCTGGGTCACTTG-3′; R: 5′-TGGCTGTTCACCCAAGTTGT-3′) and β_2_-microglobulin (*B2M*) as the reference housekeeper gene (F: 5′-CATCCAGCGTACTCCAAAGA-3′; R: 5′-GACAAGTCTGAATGCTCCAC-3′). Relative changes in mRNA expression were calculated using the ^ΔΔ^*C_t_* method, normalized to *B2M*.

### 4.5. Protein Expression

To measure protein expression, cells were lysed with radioimmunoprecipitation assay lysis buffer (20 mM Tris-HCl, 1 mM EDTA, 1 mM EGTA, 1 mM dithiothreitol, 0.5 mM phenylmethylsulfonyl fluoride, 1.5 μg/mL aprotinin, 1 μg/mL leupeptin, 1 μg/mL pepstatin, 1 mM sodium orthovanadate, and 0.2% Triton X-100; pH 7.4). Protein concentrations were determined using the bicinchoninic assay (BCA), and 8 μg of the protein extracts were loaded for Western blot analysis. These were probed with antibodies for CREBRF (sc-133747, 1:200; Santa Cruz Biotechnology, Inc., Santa Cruz, CA, USA), TRIM2 (ab123899, 1:500; Abcam, Cambridge, UK) and α-tubulin (ab40742, 1:5000; Abcam, Cambridge, UK), used to confirm equal protein loading. Protein levels were quantified from digitized images of the nitrocellulose membranes using Bio-Rad ImageLab software (Bio-Rad Laboratories).

### 4.6. Lentiviral shRNA Knockdown of CREBRF and TRIM2—Matrigel Tubulogenesis Assay

HCAECs were seeded at 5 × 10^4^ cells/well and cultured at 37 °C in 5% CO_2_. HCAECs were then exposed for 24 h to either PBS (non-transduced control) or 1 × 10^4^ infectious units (IFU)/mL of lentiviral particles containing shRNA directed against *CREBRF*, *TRIM2* or a control sequence in the presence of polybrene (8 μg/mL). After lentiviral transduction, the virus-containing media was removed and replaced with fresh MesoEndo media for 72 h. Matrigel tubulogenesis assays were used to assess the effect of *CREBRF* and *TRIM2* knockdown. Transduced HCAECs were trypsinized, counted and seeded at a density of 1.2 × 10^4^ cells/well onto growth factor-reduced Matrigel matrix, phenol red-free (40 μL; Becton Dickinson, Franklin Lakes, NJ, USA) that had been polymerized for 30 min at 37 °C. Cells were incubated for 4 h under normoxic or hypoxic conditions (1.2% O_2_ as above), or with TNFα (0.5 ng/mL) to mimic inflammatory conditions. In parallel experiments, HCAECs transduced with either shControl or shTRIM2 were treated with either PBS or rHDL for 16 h prior to Matrigel tubulogenesis assay. Tubules were photographed at x4 magnification under light microscopy and analyzed using ImageJ (U.S. National Institutes of Health, Bethesda, MD, USA) by counting the total number of tubules formed. This assay was undertaken twice, with four replicates for each condition.

### 4.7. Statistical Analyses

Values are expressed as the mean ± SEM. Differences between treatment groups were analyzed using either an unpaired two-tailed *t*-test or one-way ANOVA, or for grouped treatments, a two-way ANOVA was employed followed by post hoc analysis using Bonferroni’s multiple comparisons test. Significance was set at a value of *p* < 0.05.

## 5. Conclusions

Our current study has identified, for the first time, two novel genes *CREBRF* and *TRIM2* that are transcriptionally upregulated by rHDL, particularly through its PLPC component. *TRIM2* appears to play a critical role in angiogenesis secondary to both hypoxia and inflammation, though further in vivo studies will be needed to clarify this. Furthermore, *TRIM2* knockdown attenuated rHDL-induced tubule formation in hypoxia, suggesting that it is important in mediating the pro-angiogenic action of rHDL under physiological conditions. Characterization of such novel regulators of angiogenesis may hold the key to developing more effective targeted therapies for angiogenesis-related diseases and may help to better understand the multifunctional modulation of these by HDL.

## Supplementary Materials

Supplementary materials can be found at http://www.mdpi.com/1422-0067/19/7/1903/s1.
