# Supplementary material for: Exploring the Roles of CREBRF and TRIM2 in the Regulation of Angiogenesis by High-Density Lipoproteins
Source: Int J Mol Sci. 2018 Jun 28;19(7):1903. doi: 10.3390/ijms19071903 (PMC6073236; doi:10.3390/ijms19071903)
Supplement: Supplementary file 1 [file ijms-19-01903-s001.pdf]

Supplementary Table S1. Genes differentially expressed in human microvascular endothelial cells (HMVECs) in response to treatment with reconstituted high-density lipoproteins (rHDL) in hypoxia. Fold changes and *P*-values are shown relative to HMVECs treated with phosphate-buffered saline (PBS) alone prior to hypoxia stimulation.

Probe ID	Gene	Fold Change	*P*-value
11724634_s_at	ABCB10	0.77	1.00E-03
11716187_a_at	ABCB6 /// ATG9A	2.36	1.00E-04
11730509_s_at	ABCC5	1.73	5.00E-03
11747860_x_at	ABCE1	0.62	5.00E-03
11725515_a_at	ABCG1	0.23	2.00E-04
11720859_s_at	ABHD3	1.79	1.00E-03
11718255_at	ABHD4	2.22	2.00E-06
11727019_at	ABI2	0.69	2.00E-03
11745737_x_at	ACADVL	1.51	2.00E-04
11746009_a_at	ACAT2	2.30	6.00E-07
11755357_s_at	ACO1	0.68	1.00E-05
11716302_s_at	ACSL1	1.32	2.00E-03
11743606_a_at	ACSL3	1.49	2.00E-03
11722346_a_at	ACSS2	1.77	7.00E-06
11720784_a_at	ACTL6A	0.70	3.00E-03
11728375_x_at	ACTN1	0.72	8.00E-04
11739032_x_at	ACTR2	0.78	4.00E-03
11728643_s_at	ACTR3	0.81	9.00E-03
11718647_at	ACVR1	1.43	8.00E-03
11731110_at	ADAM17	1.59	4.00E-03
11755797_x_at	ADAM9	0.77	6.00E-03
11754662_s_at	ADAMTS1	2.39	4.00E-03
11730923_x_at	ADAMTS4	2.30	9.00E-04
11756164_s_at	ADIPOR2	1.45	2.00E-03
11716499_x_at	ADRM1	1.75	2.00E-09
11727680_a_at	ADSL	0.72	1.00E-04
11733383_a_at	AFAP1	0.58	6.00E-07
11740338_a_at	AGFG1	0.60	7.00E-03
11725496_a_at	AGPAT9	2.71	8.00E-04
11753687_x_at	AGTRAP	1.47	4.00E-05
11744648_s_at	AIFM2	2.13	8.00E-05
11751675_s_at	AK2	0.66	1.00E-04
11724490_x_at	AKR1B10	7.21	3.00E-09
11724489_s_at	AKR1B10 /// AKR1B15	3.14	3.00E-08
11742379_x_at	AKR1B15	1.56	2.00E-03
11719171_a_at	AKR1C1	4.17	2.00E-06
11754189_s_at	AKR1C1 /// AKR1C2	3.36	4.00E-04
11715711_a_at	AKR1C3	1.92	7.00E-07
11733041_a_at	ALAS1	1.72	2.00E-05
11752549_x_at	ALDH1A1	0.23	4.00E-06
11746896_a_at	ALDH2	2.31	1.00E-06
11754201_s_at	ALDH7A1	0.55	5.00E-08
11720274_x_at	ALKBH6	1.72	1.00E-06
11763990_a_at	ALKBH7	1.29	9.00E-03
11727575_a_at	ALS2CR4	0.55	4.00E-03
11748140_s_at	AMD1	0.62	2.00E-04
11737334_a_at	AMIGO2	0.52	6.00E-03
11721892_a_at	ANAPC1	0.73	8.00E-04
11721569_a_at	ANG	2.48	3.00E-05
11719004_at	ANKH	2.22	3.00E-06
11722638_at	ANKRD28	1.61	3.00E-03
11726350_at	ANKRD39	1.32	5.00E-03
11760257_a_at	ANKZF1	1.41	8.00E-03
11744787_a_at	ANLN	0.44	6.00E-04
11717412_x_at	ANO6	1.46	8.00E-05
11756300_a_at	ANP32B	0.76	7.00E-04
11756334_x_at	ANXA3	0.64	5.00E-03

11744534_a_at	ANXA7	1.61	5.00E-06
11756982_x_at	AP3S2	1.38	4.00E-03
11734823_a_at	APBB2	0.58	2.00E-05
11715398_a_at	APEX1	0.78	3.00E-03
11746855_a_at	APOA1BP	0.73	3.00E-03
11732901_a_at	APOBEC3A /// APOBEC3B	0.41	9.00E-04
11758898_x_at	APOBEC3C	0.71	3.00E-03
11737791_s_at	APOL1 /// APOL2	2.22	2.00E-03
11734915_at	ARHGAP11A	0.62	6.00E-05
11715167_at	ARHGAP22	0.53	2.00E-03
11723813_at	ARHGAP29	0.78	6.00E-03
11715466_at	ARHGDIB	0.64	4.00E-03
11739285_a_at	ARL6IP6	0.70	3.00E-03
11744690_a_at	ARMC10	0.63	1.00E-06
11758294_s_at	ARMCX1	1.73	6.00E-05
11744077_at	ARPC1A /// ARPC1B /// LOC653888	1.30	3.00E-03
11746862_x_at	ARSJ	0.51	5.00E-03
11720264_at	ASCC3	0.67	3.00E-05
11751629_a_at	ASF1B	0.60	2.00E-03
11748793_a_at	ASNS	6.02	3.00E-10
11756885_a_at	ATAD2	0.42	2.00E-06
11719344_a_at	ATF3	4.76	5.00E-03
11715755_a_at	ATIC	0.53	9.00E-06
11718081_a_at	ATP2B4	1.47	2.00E-03
11724190_s_at	ATP5B	0.77	7.00E-03
11734653_x_at	ATP5F1	0.80	1.00E-03
11720314_s_at	ATP6V0B	1.69	1.00E-06
200096_PM_s_at	ATP6V0E1	1.39	8.00E-05
11716072_s_at	ATP6V1B2	1.52	2.00E-03
11741370_a_at	ATP6V1E1	1.43	4.00E-04
11754004_a_at	ATP6V1G1	1.48	7.00E-06
200041_PM_s_at	ATP6V1G2 /// BAT1	0.61	7.00E-04
11725232_at	ATP8B2	1.89	3.00E-03
11716305_at	ATXN10	0.75	4.00E-03
11756564_a_at	AUP1	1.30	1.00E-02
11720203_a_at	AURKB	0.52	2.00E-03
11759664_a_at	B4GALNT1	2.20	2.00E-07
11716856_a_at	B4GALT3	1.34	2.00E-03
11756612_a_at	BARD1	0.46	3.00E-04
11718050_a_at	BASP1	0.62	5.00E-05
11754350_a_at	BAT4	1.32	7.00E-03
11755326_x_at	BAT5	1.35	7.00E-03
11720443_s_at	BAZ1A	0.74	1.00E-04
11715424_x_at	BCAP31	1.31	5.00E-04
11749654_x_at	BCAR1	0.62	1.00E-03
11755006_x_at	BCAR3	0.62	3.00E-05
11727544_a_at	BCAT2	0.69	2.00E-03
11743504_at	BCCIP	0.51	1.00E-05
11717364_a_at	BCL2L12	0.68	9.00E-04
11728948_at	BCMO1	1.51	2.00E-03
11758566_s_at	BCS1L	0.76	1.00E-02
11735902_a_at	BDNF	0.26	1.00E-04
11719475_a_at	BEX1	0.27	5.00E-03
11726202_s_at	BHLHB9	1.43	6.00E-03
11746667_a_at	BHMT2	2.89	2.00E-04
11754110_x_at	BIRC5	0.54	1.00E-03
11747384_x_at	BIVM	1.55	7.00E-03
11756409_a_at	BLM	0.61	2.00E-03
11738906_a_at	BLVRA	0.73	5.00E-03
11756756_a_at	BLVRB	2.46	7.00E-08
11726120_a_at	BMP2K	1.67	1.00E-03
11734013_a_at	BMP4	0.29	3.00E-07

11735400_a_at	BNC1	0.42	2.00E-03
11757102_a_at	BOP1	0.54	2.00E-06
11735584_x_at	BRCA1	0.61	1.00E-03
11719169_s_at	BRI3	1.44	5.00E-05
11759029_at	BRI3BP	0.66	3.00E-03
11736602_at	BRIP1	0.69	9.00E-03
11720330_at	BRIX1	0.64	6.00E-06
11757724_x_at	BSCL2	1.57	3.00E-03
11733263_a_at	BSDC1	1.48	2.00E-04
11723420_s_at	BTF3L4 /// LOC100505945	0.76	1.00E-02
11747643_s_at	BUB3	0.67	3.00E-05
11717444_at	BYSL	0.63	1.00E-04
11748735_s_at	BZW1	0.67	2.00E-04
11747678_x_at	BZW2	0.65	3.00E-04
11728936_at	C11orf41	0.57	9.00E-03
11718063_a_at	C11orf48	0.64	2.00E-04
11728525_at	C11orf63	1.51	4.00E-03
11723561_x_at	C11orf75	1.59	9.00E-03
11744907_a_at	C12orf11	0.64	9.00E-05
11721899_x_at	C12orf24	0.45	4.00E-06
11726112_at	C12orf26	0.64	1.00E-04
11723719_at	C12orf45	0.72	3.00E-03
11719732_at	C12orf5	0.69	2.00E-04
11728156_s_at	C13orf31	1.97	4.00E-03
11757616_s_at	C14orf1	2.33	2.00E-08
11754198_at	C14orf139	4.76	3.00E-10
11716270_a_at	C14orf156	0.67	3.00E-06
11717150_s_at	C16orf57	0.72	6.00E-03
11723885_at	C17orf103	1.80	2.00E-03
11722729_a_at	C17orf95	1.44	6.00E-03
11729885_at	C18orf54	0.59	9.00E-03
11757443_s_at	C18orf55	0.69	3.00E-03
11717536_x_at	C19orf6	1.39	7.00E-03
11757526_x_at	C19orf60	1.53	3.00E-05
11728258_at	C1orf107	1.74	2.00E-03
11742483_a_at	C1orf110	0.24	5.00E-07
11725837_a_at	C1orf112	0.59	5.00E-03
11716538_x_at	C1orf144	0.71	9.00E-03
11728096_a_at	C1orf201	1.54	8.00E-03
11725726_a_at	C1orf56	1.53	9.00E-03
11756435_a_at	C1orf85	1.55	2.00E-03
11743322_at	C20orf111	1.55	7.00E-03
11747215_x_at	C20orf24	1.54	3.00E-06
11724506_s_at	C21orf45	0.63	2.00E-03
11747047_a_at	C21orf7	2.33	5.00E-07
11736220_x_at	C22orf25	1.85	4.00E-07
11725352_a_at	C22orf9	1.53	7.00E-04
11720690_a_at	C2orf18	1.52	2.00E-03
11739790_a_at	C2orf3	0.71	9.00E-03
11751637_a_at	C3orf21	0.68	6.00E-03
11716982_at	C3orf26	0.68	4.00E-03
11722937_s_at	C3orf59	0.47	7.00E-05
11743406_x_at	C4orf34	2.00	1.00E-06
11728745_at	C4orf46	0.63	7.00E-03
11727935_at	C4orf49	0.17	1.00E-03
11737948_a_at	C5	0.65	2.00E-03
11724481_a_at	C5orf13	0.45	4.00E-04
11716579_a_at	C5orf32	1.71	1.00E-05
11730128_a_at	C5orf35	0.67	6.00E-03
11761149_a_at	C5orf45	2.17	4.00E-05
11721051_at	C5orf51	1.32	6.00E-03
11756876_a_at	C6orf125	0.66	2.00E-05

11718540_at	C7orf23	1.42	7.00E-03
11718604_a_at	C7orf41	1.79	1.00E-03
11743161_x_at	C7orf68	0.74	4.00E-03
11725003_s_at	C8orf58	0.68	7.00E-04
11716890_a_at	C9orf123	0.78	9.00E-03
11725149_at	C9orf40	0.74	6.00E-03
11720532_s_at	C9orf72	2.35	6.00E-04
11718531_a_at	CAD	0.66	9.00E-04
11744396_x_at	CALCOCO2	1.56	8.00E-05
11730274_at	CALCRL	2.31	6.00E-07
11740134_a_at	CALD1	0.67	6.00E-04
11754149_s_at	CALM2	0.64	4.00E-06
11717268_a_at	CAMK2G	1.43	7.00E-05
11736080_a_at	CAMLG	1.40	3.00E-03
11727424_s_at	CANX	1.64	3.00E-03
11728617_at	CARD6	1.29	5.00E-03
11716843_a_at	CASC4	1.44	3.00E-05
11748529_x_at	CASP4	1.52	3.00E-03
11715584_a_at	CAV1	0.62	2.00E-05
11753301_a_at	CBR1	1.31	2.00E-03
11744285_a_at	CBS	2.03	3.00E-03
11715546_s_at	CBX1	0.64	6.00E-04
11754967_s_at	CBX3	0.70	8.00E-04
11716276_a_at	CBX5	0.66	5.00E-03
11749706_a_at	CCDC138	0.58	4.00E-05
11751479_a_at	CCDC152	0.75	7.00E-03
11721904_a_at	CCDC28B	0.70	7.00E-03
11758016_s_at	CCDC34	0.63	5.00E-03
11727311_a_at	CCDC41	0.73	6.00E-03
11743324_a_at	CCDC50	0.79	9.00E-03
11739483_a_at	CCDC88A	0.66	9.00E-04
11716384_at	CCL2	0.18	8.00E-05
11719198_s_at	CCND2	0.49	9.00E-03
11728301_a_at	CCNE2	0.43	1.00E-05
11722833_a_at	CCNY	0.74	9.00E-04
11750355_a_at	CCPG1	2.99	6.00E-06
11741990_s_at	CCRL2	2.41	4.00E-03
11715421_a_at	CCT2	0.65	5.00E-07
11716383_x_at	CCT3	0.74	1.00E-05
11749821_x_at	CCT4	0.81	3.00E-03
11753081_a_at	CCT5	0.65	3.00E-04
11737913_s_at	CCT7	0.75	3.00E-04
11715918_s_at	CD24	0.43	7.00E-03
11739616_x_at	CD3EAP	0.68	8.00E-03
11731818_a_at	CD40	2.04	5.00E-03
11743903_a_at	CD46	1.36	3.00E-03
11743348_a_at	CD47	1.37	7.00E-03
11755084_x_at	CD55	1.84	6.00E-06
11748314_a_at	CDC20	0.50	2.00E-03
11726756_a_at	CDC25A	0.43	8.00E-05
11721886_a_at	CDC42	0.73	1.00E-02
11751123_a_at	CDC45	0.57	2.00E-04
11743065_at	CDC6	0.36	4.00E-03
11755466_a_at	CDCA2	0.57	9.00E-03
11719598_s_at	CDCA3	0.56	7.00E-04
11718183_at	CDCA5	0.50	9.00E-05
11758478_s_at	CDCA7	0.23	6.00E-10
11717436_a_at	CDCA7L	0.46	6.00E-06
11759413_s_at	CDH11	0.48	2.00E-03
11726614_at	CDH2	0.70	6.00E-03
11745843_a_at	CDK1	0.52	7.00E-03
11724959_s_at	CDK14	0.67	7.00E-04

11719253_at	CDK19	1.55	3.00E-03
11716622_x_at	CDK2AP2	1.88	1.00E-05
11729118_a_at	CDKN1C	1.43	5.00E-03
11753763_x_at	CDKN3	0.56	5.00E-03
11722949_at	CDRT4	1.74	5.00E-03
11721666_at	CDT1	0.67	8.00E-03
11723464_s_at	CELF2	0.48	7.00E-08
11739328_x_at	CENPH	0.61	1.00E-03
11755168_x_at	CENPL	0.65	2.00E-03
11727353_a_at	CENPM	0.63	1.00E-03
11725788_a_at	CENPN	0.68	8.00E-03
11720827_a_at	CENPW	0.54	1.00E-03
11720247_a_at	CEP55	0.55	4.00E-03
11720016_at	CGNL1	0.59	9.00E-03
11728808_at	CHAC2	0.58	8.00E-04
11727879_s_at	CHAF1A	0.70	4.00E-03
11721851_x_at	CHCHD6	0.70	1.00E-03
11729663_a_at	CHEK1	0.51	1.00E-04
11741135_s_at	CHN1	0.55	8.00E-04
11717460_a_at	CHPF2	1.66	4.00E-04
11754623_x_at	CHPT1	1.27	3.00E-03
11723973_at	CHST12	1.38	6.00E-04
11757950_s_at	CHSY1	0.61	6.00E-07
11717511_a_at	CIRH1A	0.62	2.00E-04
11726583_s_at	CKAP4	1.23	5.00E-03
11753856_a_at	CKS2	0.58	2.00E-05
11743022_a_at	CLCC1	1.53	3.00E-03
11725767_at	CLCF1	1.40	7.00E-03
11742798_a_at	CLCN3	1.65	7.00E-03
11729619_at	CLCN4	0.54	2.00E-04
11720435_s_at	CLCN6	1.57	2.00E-03
11719182_a_at	CLCN7	1.42	8.00E-03
11747704_a_at	CLDN11	0.43	3.00E-07
11727413_at	CLN8	1.67	5.00E-03
11754044_at	CLSPN	0.54	2.00E-05
11726437_a_at	CMTM8	2.41	7.00E-04
11744825_a_at	CNBP	0.74	5.00E-04
11754644_x_at	CNN2	0.61	4.00E-06
11757481_a_at	COPS6	0.70	9.00E-05
11719224_s_at	COQ10A	1.51	6.00E-03
11715524_a_at	COTL1	0.57	5.00E-03
11733802_a_at	CPPED1	0.75	3.00E-03
11744958_x_at	CPSF6	0.73	9.00E-03
11756474_a_at	CPT1A	1.52	4.00E-04
11718556_s_at	CREB3L2	0.66	7.00E-04
11722883_a_at	CREBRF	1.99	2.00E-04
11719327_a_at	CRELD2	2.06	3.00E-06
11730763_x_at	CRIPT	1.43	6.00E-04
11729632_a_at	CSDA	0.75	2.00E-04
11746367_a_at	CSE1L	0.54	3.00E-03
11722981_a_at	CSPG5	0.35	1.00E-05
11730215_a_at	CSRP1	0.43	3.00E-06
11728320_a_at	CSTB	1.29	6.00E-03
11727580_x_at	CSTF3	0.57	2.00E-03
11763238_x_at	CTCFL /// HMGB1	0.67	7.00E-03
11720494_a_at	CTNNAL1	0.39	4.00E-06
11749064_x_at	CTNS	1.66	8.00E-04
11743908_a_at	CTPS	0.57	6.00E-07
11715710_s_at	CTSC	0.65	1.00E-03
11755925_s_at	CTSD	1.77	6.00E-03
11725332_a_at	CTSL1	1.41	4.00E-03
11754114_a_at	CXCL1	0.25	3.00E-04

11763250_x_at	CXCL1 /// CXCL2	0.24	1.00E-04
11721692_x_at	CYB561	2.03	8.00E-03
11756999_a_at	CYB561D2	1.51	2.00E-03
11748855_a_at	CYB5R1	2.30	6.00E-07
11758590_s_at	CYBRD1	1.84	9.00E-04
11757429_a_at	CYC1	0.77	3.00E-03
11717676_a_at	CYFIP2	0.49	4.00E-04
11722563_x_at	CYP51A1	2.60	1.00E-03
11716317_at	CYTH2	1.38	1.00E-02
11720146_a_at	DAPK1	0.57	7.00E-04
11726602_at	DARS2	0.77	6.00E-03
11752175_x_at	DBF4	0.63	3.00E-03
11730327_s_at	DCAF13	0.72	4.00E-03
11736132_s_at	DCBLD2	0.67	2.00E-03
11726670_a_at	DCLRE1B	0.65	1.00E-04
11743061_at	DCTPP1	0.75	7.00E-04
11750080_a_at	DDB2	0.71	9.00E-03
11724796_at	DDIT4L	3.76	7.00E-06
11716708_a_at	DDR1	2.25	5.00E-04
11759442_s_at	DDR2	2.16	2.00E-04
11718598_x_at	DDRGK1	1.42	5.00E-03
11755268_a_at	DDX18	0.77	1.00E-02
11756223_s_at	DDX21	0.59	3.00E-06
11724298_a_at	DDX31	0.74	8.00E-03
11715903_at	DDX3X	0.76	3.00E-03
11715849_a_at	DDX47	0.70	2.00E-04
11743651_a_at	DEK	0.63	5.00E-03
11746252_a_at	DENR	0.73	1.00E-03
11719194_a_at	DFNA5	1.43	4.00E-03
11730959_a_at	DGKG	2.30	2.00E-04
11715460_a_at	DHCR24	2.35	6.00E-08
11743808_a_at	DHCR7	2.46	4.00E-06
11747280_x_at	DHFR	0.60	4.00E-03
11729960_x_at	DHFRL1	1.45	3.00E-03
11749344_a_at	DHRS3	0.68	9.00E-03
11744862_x_at	DHRS7	1.39	2.00E-03
11743114_at	DHX15	0.68	3.00E-05
11727949_at	DHX33	0.76	7.00E-03
11752968_a_at	DHX9	0.73	5.00E-04
11722339_a_at	DIAPH2	1.85	4.00E-05
11752028_a_at	DIAPH3	0.49	8.00E-04
11748536_a_at	DIMT1L	0.75	3.00E-03
11724416_at	DISP1	1.93	5.00E-06
11743467_at	DKC1	0.56	1.00E-05
11763480_x_at	DLEU2	0.47	8.00E-03
11741748_a_at	DLG1	0.41	2.00E-03
11724711_a_at	DMXL1	1.87	5.00E-04
11717189_x_at	DNAJA1	0.76	4.00E-03
11742905_a_at	DNAJC1	1.58	3.00E-06
11752102_s_at	DNAJC10	1.61	4.00E-04
11717372_s_at	DNAJC3	1.99	2.00E-03
11731987_a_at	DNAJC9	0.57	4.00E-06
11763164_at	DNAJC9 /// MRPS16	0.58	1.00E-05
11730732_at	DNASE2	1.40	2.00E-03
11737604_a_at	DNM1L	0.75	5.00E-03
11736405_a_at	DNMT1	0.56	9.00E-03
11724825_a_at	DOLK	1.48	4.00E-05
11724208_a_at	DPH2	0.72	2.00E-04
11755170_s_at	DPY30 /// MEMO1	0.77	9.00E-04
11728729_at	DSCC1	0.62	5.00E-03
11719941_s_at	DSCR3	1.31	8.00E-03
11727219_a_at	DSE	1.69	8.00E-03

11730623_at	DSEL	0.54	2.00E-03
11723120_x_at	DSTYK	1.27	4.00E-03
11726302_a_at	DTL	0.26	1.00E-06
11716642_s_at	DTYMK	0.71	2.00E-03
11726596_x_at	DUT	0.55	4.00E-04
11724908_a_at	DYNC1I1	1.73	3.00E-05
11719373_at	DYNLT3	1.42	5.00E-03
11722703_a_at	DZIP1	0.69	1.00E-03
11728957_a_at	E2F8	0.63	5.00E-04
11748565_a_at	EBNA1BP2	0.52	2.00E-08
11749332_x_at	EBP	1.72	9.00E-07
11745833_s_at	ECT2	0.65	4.00E-03
11722261_at	EDEM1	1.60	4.00E-03
11747223_a_at	EDNRB	2.58	4.00E-03
11750166_a_at	EED	0.76	5.00E-03
11724920_a_at	EFCAB7	1.39	4.00E-03
11749564_a_at	EFEMP1	0.65	2.00E-06
11715913_s_at	EFNB1	1.92	4.00E-05
11753665_x_at	EGLN1	0.70	1.00E-03
11750276_x_at	EHD1	0.67	5.00E-03
11719596_at	EHD3	1.73	7.00E-03
11720919_s_at	EIF1	1.44	1.00E-04
11716778_s_at	EIF1AX	0.74	8.00E-03
11759312_at	EIF2C4	1.51	2.00E-03
11716220_x_at	EIF2S1	0.71	3.00E-04
11725638_a_at	EIF3J	0.78	3.00E-03
11762854_a_at	EIF4A1	0.66	5.00E-03
11744531_x_at	EIF5B	0.72	5.00E-03
11716529_a_at	ELAVL1	0.73	8.00E-03
11757300_s_at	ELOVL5	1.33	2.00E-03
11728708_at	EMP2	0.62	1.00E-04
11729574_at	ENC1	0.39	4.00E-05
11753811_x_at	ENY2	0.77	1.00E-02
11722358_a_at	EPB41L2	0.54	3.00E-03
11727142_a_at	EPB41L3	0.46	9.00E-03
11759882_a_at	EPS15L1	1.66	2.00E-03
11717915_s_at	ERCC5	1.60	2.00E-04
11726441_a_at	ERCC6L	0.58	8.00E-04
11716644_a_at	ERLEC1	1.57	9.00E-04
11720444_s_at	ERLIN1	0.78	1.00E-03
11730914_a_at	ERLIN1	0.70	2.00E-03
11734857_at	ERO1LB	2.83	4.00E-03
11718354_a_at	ERP44	1.51	3.00E-03
11715659_s_at	ERRFI1	0.42	4.00E-03
11727968_at	ESCO2	0.38	1.00E-05
11729647_s_at	ESM1	1.53	3.00E-04
11721451_a_at	ETF1	0.64	5.00E-03
11721261_a_at	ETNK2	1.46	3.00E-03
11721209_a_at	EXO1	0.55	5.00E-04
11731563_a_at	EXOSC7	0.80	8.00E-03
11753011_a_at	EXOSC9	0.68	1.00E-03
11717223_a_at	EXTL3	1.37	5.00E-03
11748690_a_at	EZH2	0.63	1.00E-02
11742987_s_at	EZR	0.64	3.00E-03
11728828_at	F2RL1	0.32	4.00E-04
11745459_a_at	FADS1	2.03	4.00E-08
11754939_x_at	FADS2	1.88	2.00E-07
11741413_a_at	FAM101A	0.22	2.00E-04
11754295_a_at	FAM107B	0.45	6.00E-04
11730690_a_at	FAM111A	0.68	9.00E-04
11741492_x_at	FAM111B	0.31	9.00E-07
11739207_x_at	FAM115A	0.66	4.00E-03

11719147_at	FAM117A	1.73	3.00E-04
11755895_a_at	FAM129A	8.40	9.00E-09
11720350_a_at	FAM129B	0.72	1.00E-02
11717908_s_at	FAM136A	0.66	7.00E-04
11744779_a_at	FAM13A	0.64	7.00E-03
11755583_a_at	FAM161A	0.62	7.00E-03
11729671_s_at	FAM200B	0.73	3.00E-03
11726052_s_at	FAM35A /// FAM35B /// FAM35B2	0.77	7.00E-03
11722342_a_at	FAM38B	0.45	2.00E-05
11733480_x_at	FAM48A	0.78	9.00E-04
11744630_a_at	FAM55C	1.58	4.00E-04
11716745_s_at	FAM60A	0.68	1.00E-03
11723747_a_at	FAM64A	0.50	3.00E-03
11746563_x_at	FAM65A	1.28	4.00E-03
11736479_s_at	FAM72A /// FAM72B /// FAM72C /// FAM72D	0.67	6.00E-03
11739159_at	FAM8A1	1.44	1.00E-04
11744666_at	FAN1	1.80	8.00E-04
11756910_x_at	FANCD2	0.54	4.00E-03
11729458_a_at	FANCI	0.55	4.00E-04
11758994_at	FAR2	0.55	6.00E-03
11756383_a_at	FARSB	0.73	3.00E-04
11756878_a_at	FBL	0.65	4.00E-05
11740804_a_at	FBXL13	2.51	1.00E-03
11757243_s_at	FBXL20	1.47	1.00E-02
11725583_at	FBXO10	1.57	3.00E-05
11747620_a_at	FBXO22	0.68	7.00E-04
11724963_s_at	FBXO5	0.40	1.00E-04
11754259_x_at	FDFT1	2.57	1.00E-08
11718366_s_at	FDPS	1.46	9.00E-03
11758249_s_at	FDPS	1.46	9.00E-03
11748117_a_at	FDXR	0.66	2.00E-03
11751388_a_at	FEN1	0.44	4.00E-06
11718181_s_at	FERMT2	0.75	2.00E-03
11747232_a_at	FGD4	0.55	3.00E-03
11741105_a_at	FHL2	0.44	1.00E-06
11731068_s_at	FIGNL1	0.65	3.00E-03
11725155_at	FJX1	0.33	1.00E-06
11733701_a_at	FKBP11	1.80	2.00E-05
11733891_at	FKBP14	1.33	4.00E-03
11718992_a_at	FKBP2	1.39	7.00E-04
11719478_a_at	FKBP3	0.62	9.00E-07
11754357_a_at	FLNB	0.57	4.00E-05
11744631_a_at	FLT1	2.14	1.00E-04
11756257_a_at	FLYWCH1	1.39	8.00E-03
11717718_at	FNDC3B	0.74	9.00E-03
11736507_s_at	FRMD6	0.47	6.00E-04
11715651_s_at	FSTL1	0.75	8.00E-03
11752577_at	FTH1	1.95	5.00E-04
11757787_x_at	FTL	1.67	8.00E-03
11752399_a_at	FUBP1	0.75	8.00E-04
11745190_a_at	FUS	0.62	8.00E-06
11757655_a_at	FXC1	1.43	1.00E-04
11732322_at	FXN	0.71	1.00E-04
11744233_at	FZD8	0.70	5.00E-03
11716581_a_at	G3BP2	0.78	8.00E-03
11737878_a_at	GALNT2	0.60	9.00E-04
11758592_s_at	GAR1	0.70	3.00E-03
11720969_a_at	GART	0.64	8.00E-03
11719908_a_at	GBA /// GBAP1	1.39	9.00E-03
11725314_a_at	GBAS	0.68	6.00E-03
11720049_a_at	GBE1	1.51	2.00E-05
11726329_x_at	GBP1	0.42	8.00E-06

11723489_at	GCLM	1.77	3.00E-03
11735270_a_at	GCNT1	0.57	1.00E-05
11733746_x_at	GCNT2	2.28	2.00E-05
11723377_a_at	GCOM1	1.83	9.00E-03
11757936_s_at	GCSH /// LOC100329108 /// LOC100329109 /// LOC641746	0.72	4.00E-03
11742888_s_at	GCSH /// LOC100329108 /// LOC729080	0.65	1.00E-03
11716663_a_at	GDF15	2.01	9.00E-03
11734363_at	GDF6	0.06	1.00E-14
11742740_at	GEMIN5	0.66	2.00E-04
11724225_a_at	GGCT	0.67	2.00E-03
11728863_at	GINS1	0.34	1.00E-05
11743513_a_at	GINS2	0.23	3.00E-12
11736597_x_at	GINS4	0.55	1.00E-03
11716718_at	GLA	2.69	3.00E-07
11722307_at	GLCE	0.56	2.00E-03
11720063_a_at	GLIPR2	0.49	2.00E-04
11726309_a_at	GLRB	0.67	9.00E-03
11718048_a_at	GLRX	1.96	1.00E-03
11737747_a_at	GMFB	0.64	4.00E-03
11754937_a_at	GMNN	0.59	2.00E-04
11739065_a_at	GNG12	0.53	3.00E-03
11716471_at	GNL2	0.75	5.00E-03
11749333_a_at	GNL3	0.59	5.00E-03
11723731_at	GNPNAT1	0.72	1.00E-02
11721648_at	GPATCH4	0.67	7.00E-04
11718503_a_at	GPCPD1	1.80	6.00E-04
11726660_a_at	GPN3	0.66	8.00E-04
11724463_a_at	GPNMB	1.54	7.00E-04
11725191_s_at	GPR126	0.48	9.00E-04
11739965_a_at	GPR39	0.57	2.00E-04
11716395_a_at	GPR56	1.67	3.00E-03
11719792_at	GPRC5A	0.66	8.00E-03
11730170_a_at	GPX3	4.26	3.00E-06
11729644_a_at	GPX8	1.89	5.00E-03
11718223_a_at	GSPT1	0.72	4.00E-03
11756634_a_at	GSR	1.83	3.00E-03
11733850_x_at	GTF3A	0.71	6.00E-04
11759176_at	GTSE1	0.57	3.00E-03
11724160_a_at	GXYLT1	0.62	1.00E-03
11758616_s_at	H2AFV	0.70	4.00E-03
11754243_a_at	H2AFX	0.69	5.00E-03
11735189_s_at	H2AFY	0.80	5.00E-03
11729614_x_at	H2AFZ	0.65	1.00E-03
11722555_s_at	HADH	0.64	1.00E-03
11723254_a_at	HAT1	0.71	7.00E-04
11753284_x_at	HAUS7	0.59	4.00E-04
11747411_a_at	HAUS7 /// TREX2	0.63	3.00E-03
11717036_a_at	HDAC5	2.75	2.00E-06
11736838_a_at	HDAC9	1.79	6.00E-03
11735104_x_at	HDLBP	1.27	2.00E-03
11755789_a_at	HEATR1	0.71	2.00E-04
11733539_a_at	HEATR7A	1.61	3.00E-03
11749691_a_at	HELLS	0.51	4.00E-06
11723889_a_at	HELQ	1.37	3.00E-03
11725920_a_at	HEXIM2	1.45	3.00E-04
11759416_x_at	HFE	1.55	5.00E-03
11726245_s_at	HIST1H4C	0.54	4.00E-03
11759258_at	HIST2H2AC	0.51	1.00E-05
11743160_a_at	HJURP	0.54	4.00E-03
11723017_x_at	HM13	1.71	4.00E-05
11739026_x_at	HMGB1	0.65	2.00E-03
11743687_s_at	HMGB2	0.60	1.00E-03

11727374_s_at	HMGCR	3.89	3.00E-12
11716987_a_at	HMGCS1	3.78	2.00E-11
11753812_x_at	HMGN1	0.69	7.00E-04
11724740_x_at	HMGN2	0.60	2.00E-05
11723537_a_at	HMHA1	1.84	8.00E-04
11720141_a_at	HMMR	0.55	7.00E-03
11753445_a_at	HMOX1	3.16	2.00E-03
11717727_s_at	HN1	0.62	5.00E-05
11728659_a_at	HN1L	0.69	7.00E-03
11758264_s_at	HNRNPA0	0.68	2.00E-03
11742679_s_at	HNRNPA2B1	0.66	2.00E-03
11723201_s_at	HNRNPA3	0.62	9.00E-03
11723199_s_at	HNRNPA3 /// HNRNPA3P1	0.72	7.00E-04
11718220_x_at	HNRNPAB	0.67	2.00E-05
11756337_x_at	HNRNPC	0.80	6.00E-03
11749303_s_at	HNRNPD	0.61	2.00E-04
11749786_x_at	HNRNPF	0.75	6.00E-03
11743099_a_at	HNRNPH1	0.75	2.00E-03
11716659_a_at	HNRNPH3	0.69	4.00E-03
11726680_s_at	HNRNPK	0.81	7.00E-03
200072_PM_s_at	HNRNPM	0.66	2.00E-05
11722001_a_at	HNRNPU	0.73	6.00E-04
11746949_x_at	HNRPDL	0.54	2.00E-06
11758015_s_at	HPRT1	0.64	2.00E-05
11722490_a_at	HS1BP3	1.79	3.00E-04
11733913_at	HS3ST1	3.68	3.00E-04
11726476_at	HS6ST3	2.31	6.00E-03
11730778_a_at	HSD17B11	1.59	1.00E-02
11757575_x_at	HSD17B14	1.99	7.00E-05
11747333_a_at	HSD17B4	1.32	9.00E-03
11732374_x_at	HSD17B7	2.16	2.00E-04
11753260_x_at	HSP90B1	1.65	4.00E-03
11719069_s_at	HSPA13	1.80	2.00E-03
11728697_a_at	HSPA4L	0.63	2.00E-03
11715775_a_at	HSPB8	3.43	1.00E-06
11753018_x_at	HSPD1	0.70	5.00E-03
11753996_x_at	HSPE1	0.66	1.00E-04
11732375_a_at	HTATIP2	1.73	1.00E-03
11744584_a_at	HYI	0.37	3.00E-07
11748624_a_at	HYOU1	2.23	4.00E-04
11763184_at	IDE	1.38	1.00E-03
11746174_s_at	IDH1	2.06	1.00E-08
11737793_a_at	IDH3A	0.74	6.00E-03
11739383_a_at	IDI1	2.97	2.00E-06
11732133_a_at	IDS	1.37	4.00E-03
11720551_a_at	IFFO1	1.56	7.00E-03
11729406_a_at	IFNGR1	1.40	9.00E-03
11755876_a_at	IFRD2	0.65	7.00E-05
11757763_s_at	IGF2BP2	0.62	4.00E-04
11716128_at	IGF2R	1.58	3.00E-05
11717909_at	IGFBP6	0.41	9.00E-06
11757033_a_at	IL13RA2	5.62	3.00E-03
11729099_a_at	IL1RAP	0.71	3.00E-03
11759145_s_at	IL1RL1	0.53	3.00E-03
11715402_x_at	ILF2	0.77	4.00E-03
11740781_a_at	ILF3	0.58	4.00E-03
11728799_at	IMMP2L	0.53	9.00E-04
11752369_a_at	IMPDH2	0.69	6.00E-03
11745624_s_at	INADL	1.72	4.00E-04
11757812_s_at	INPP5A	1.84	1.00E-05
11718454_a_at	INPP5K	1.57	1.00E-04
11716339_a_at	INSIG1	3.68	8.00E-09

11727540_a_at	INTS7	0.66	1.00E-04
11718106_s_at	IPO5	0.56	2.00E-04
11758225_s_at	IQGAP2	0.62	7.00E-03
11719099_at	ISOC1	0.71	1.00E-03
11727715_at	ITGA10	2.22	2.00E-03
11735062_at	ITGB3	0.42	1.00E-03
11745297_x_at	ITGB3BP	0.62	3.00E-03
11758527_s_at	ITPR2	0.45	7.00E-03
11722551_s_at	ITSN1	0.55	2.00E-04
11741235_a_at	JKAMP	1.49	2.00E-03
11755160_a_at	JOSD1	1.41	1.00E-04
11726013_a_at	JUB	0.59	1.00E-03
11733807_a_at	KARS	0.80	3.00E-03
11715574_a_at	KDELR2	1.46	1.00E-03
11716574_at	KDM3A	1.40	5.00E-03
11719725_at	KIAA0020	0.71	8.00E-04
11744425_a_at	KIAA0101	0.61	6.00E-03
11746388_x_at	KIAA0146	0.71	5.00E-03
11719432_at	KIAA0513	1.71	2.00E-03
11717806_a_at	KIAA0913	1.43	1.00E-03
11760258_x_at	KIAA1324	1.66	5.00E-03
11742763_a_at	KIAA1370	1.61	7.00E-05
11736610_a_at	KIAA1432	0.63	2.00E-03
11756918_a_at	KIF23	0.52	2.00E-03
11720107_s_at	KIF2A	0.74	9.00E-03
11737197_s_at	KIF4A /// KIF4B	0.54	9.00E-03
11728954_a_at	KITLG	5.06	4.00E-06
11727145_s_at	KLF11	2.66	1.00E-06
11758208_s_at	KLF2	1.48	1.00E-02
11736903_at	KLHDC1	1.42	2.00E-03
11756499_a_at	KLHDC2	1.35	6.00E-03
11718984_a_at	KLHL21	1.72	2.00E-03
11760229_x_at	KLHL23	0.61	8.00E-03
11756440_a_at	KLHL24	1.96	7.00E-04
11722120_a_at	KNTC1	0.67	3.00E-03
11754765_s_at	KPNA2 /// LOC146880 /// PLEKHM1P	0.71	2.00E-04
11743116_s_at	KPNB1	0.76	2.00E-03
11754941_x_at	KRT18	0.36	2.00E-09
11715683_a_at	KRT7	0.35	1.00E-05
11716269_s_at	KRTCAP2	1.27	2.00E-03
11726965_a_at	L3MBTL3	1.61	3.00E-04
11744265_a_at	LAMB2	1.51	4.00E-03
11715629_a_at	LAPTM4A	1.31	7.00E-04
11727773_at	LARP6	2.48	2.00E-06
11721074_a_at	LAS1L	0.65	5.00E-06
11754581_s_at	LASS6	0.67	9.00E-04
11756099_a_at	LDHB	0.73	7.00E-04
11720029_a_at	LDLR	2.77	2.00E-08
11723233_a_at	LEO1	0.76	8.00E-03
11757652_x_at	LEPRE1	1.31	2.00E-03
11726299_x_at	LGALS8	1.84	2.00E-04
11734594_a_at	LIN7B	2.25	3.00E-05
11725469_at	LIPG	2.30	2.00E-05
11724212_at	LMAN1	1.61	7.00E-04
11750441_a_at	LMNB1	0.62	4.00E-03
11750442_s_at	LMNB1 /// PCIF1	0.46	5.00E-04
11716170_at	LMNB2	0.68	5.00E-03
11757703_x_at	LOC100130932 /// SNRPG	0.71	2.00E-04
11737378_s_at	LOC100132288 /// MAFIP /// TEKT4P1	1.35	2.00E-03
11745281_at	LOC100287896	1.54	3.00E-03
11763303_at	LOC100506748	1.73	2.00E-05
11719965_s_at	LOC100509022 /// U2AF1	0.74	4.00E-04

11731632_s_at	LOC392288 /// MAP1LC3B	1.97	3.00E-05
11718376_s_at	LOC401127 /// WDR5	0.70	5.00E-04
11756139_s_at	LOC728554 /// THOC3	0.73	3.00E-03
11755167_s_at	LOC729678	1.47	3.00E-03
11751288_a_at	LOXL2	0.57	4.00E-03
11715638_s_at	LPCAT1	1.46	9.00E-03
11754573_a_at	LPIN1	2.20	4.00E-04
11722506_a_at	LPPR2	1.33	4.00E-03
11742795_at	LRP10	1.39	2.00E-03
11728896_a_at	LRP8	2.08	3.00E-05
11726291_a_at	LRRC17	0.24	2.00E-04
11728405_s_at	LRRC33	2.04	1.00E-04
11723248_at	LRRC40	0.71	2.00E-04
11729414_s_at	LRRC8A	1.33	7.00E-04
11725899_a_at	LRRC8D	0.62	2.00E-05
11757324_s_at	LSM12	0.79	3.00E-03
11717390_a_at	LSM2	0.73	4.00E-03
11753808_s_at	LSM3	0.74	3.00E-03
11723958_s_at	LSM5	0.65	9.00E-04
11724199_x_at	LSS	1.84	6.00E-04
11720527_at	LTBP2	0.60	3.00E-03
11746136_a_at	LTV1	0.69	4.00E-03
11725730_a_at	LUC7L	0.71	9.00E-04
11728507_at	LY96	2.58	3.00E-06
11722839_at	LYAR	0.64	6.00E-04
11718084_a_at	LYN	1.83	3.00E-03
11739166_a_at	LYPD1	0.47	7.00E-03
11720185_a_at	MAD2L1	0.46	2.00E-04
11717133_a_at	MAFG	1.41	2.00E-03
11755486_s_at	MAGT1	1.92	4.00E-10
11725392_a_at	MAK16	0.69	2.00E-03
11723448_x_at	MALL	4.35	3.00E-04
11736101_at	MAN1A1	3.14	9.00E-09
11757369_s_at	MAN2B1	1.36	1.00E-03
11727029_a_at	MAP1A	2.41	2.00E-07
11748422_a_at	MAP1LC3B	1.92	1.00E-04
11736559_a_at	MAP2	2.75	8.00E-07
11719297_s_at	MAPKAP1	0.81	8.00E-03
11715587_at	MAPRE1	0.77	8.00E-04
11742948_at	MARCKS	0.51	2.00E-04
11729711_at	MARS2	0.57	2.00E-03
11759019_x_at	MATR3	0.68	5.00E-03
11729236_at	MCCC2	0.66	7.00E-04
11715487_a_at	MCL1	0.65	2.00E-03
11736367_a_at	MCM10	0.43	1.00E-05
11746594_a_at	MCM2	0.48	9.00E-05
11748174_x_at	MCM3	0.50	3.00E-09
11759510_x_at	MCM4	0.40	8.00E-06
11732997_a_at	MCM6	0.37	4.00E-07
11753558_x_at	MCM7	0.48	2.00E-06
11734971_s_at	MCM8	0.67	6.00E-05
11718554_a_at	MCOLN1	1.57	1.00E-03
11763965_s_at	MDN1	0.61	7.00E-07
11750913_a_at	ME1	2.57	5.00E-03
11739452_a_at	ME2	0.70	8.00E-04
11732580_a_at	ME3	0.65	7.00E-04
11734794_a_at	MED31	1.35	5.00E-03
11721563_a_at	MELK	0.53	6.00E-05
11723029_x_at	MEST	0.16	4.00E-03
11747170_x_at	METTL2B	0.70	5.00E-03
11752336_x_at	MFSD11	1.72	4.00E-06
11720534_a_at	MFSD5	1.32	9.00E-03

11726667_a_at	MFSD8	1.49	9.00E-03
11715706_x_at	MGEA5	1.45	4.00E-03
11724471_x_at	MGST1	1.82	8.00E-03
11753715_a_at	MGST2	1.65	3.00E-03
11716165_a_at	MGST3	1.36	9.00E-03
11761440_a_at	MICAL2	0.39	2.00E-04
11729389_a_at	MIER1	0.73	7.00E-03
11727083_a_at	MINA	0.67	2.00E-05
11750821_x_at	MIPOL1	0.68	1.00E-04
11718657_a_at	MIR21 /// TMEM49	1.57	1.00E-04
11721145_s_at	MKI67	0.52	9.00E-03
11718558_s_at	MKRN1	1.53	8.00E-04
11729648_at	MLF1IP	0.59	5.00E-03
11753830_a_at	MLLT11	3.07	4.00E-04
11720537_at	MMAB	1.65	3.00E-03
11736866_a_at	MMP16	0.54	1.00E-03
11733445_at	MMS22L	0.67	3.00E-03
11753000_s_at	MOBP	0.62	9.00E-03
11727450_a_at	MORN4	1.69	7.00E-03
11731778_a_at	MOSC1	1.57	2.00E-04
11764146_s_at	MOSPD2	1.46	7.00E-03
11726721_x_at	MPG	1.37	5.00E-03
11743466_at	MPHOSPH6	0.58	7.00E-06
11719834_a_at	MPZL2	0.42	4.00E-03
11763627_a_at	MRPL12	0.69	3.00E-04
11715772_x_at	MRPL13	0.77	5.00E-03
11727410_a_at	MRPL19	0.80	1.00E-02
11717643_a_at	MRPL24	0.73	2.00E-03
11757551_a_at	MRPL3	0.68	6.00E-05
11741883_a_at	MRPL39	0.71	7.00E-03
11743806_x_at	MRPL42	0.70	3.00E-03
11730212_a_at	MRPS7	0.78	8.00E-03
11724751_at	MRTO4	0.59	4.00E-04
11755368_a_at	MSH6	0.58	3.00E-06
11757884_s_at	MSRB3	0.73	7.00E-03
11726117_a_at	MTAP	0.72	1.00E-02
11716667_a_at	MTHFD1	0.49	6.00E-07
11728986_x_at	MTHFD1L	0.73	5.00E-03
11727179_a_at	MTHFR	1.57	2.00E-03
11739413_at	MTR	0.69	4.00E-03
11715395_a_at	MUTED /// TXNDC5	0.75	5.00E-03
11729695_a_at	MVD	1.79	6.00E-07
11747747_a_at	MVK	1.69	5.00E-03
11725861_a_at	MYBL1	0.38	3.00E-06
11744169_a_at	MYBL2	0.55	2.00E-05
11727802_s_at	MYCBP	0.69	2.00E-03
11754984_s_at	MYH10	0.47	1.00E-03
11739009_a_at	MYH9	0.74	6.00E-04
11718007_a_at	MYLIP	0.46	2.00E-03
11721837_a_at	MYLK	6.50	5.00E-08
11718068_at	MYO1E	1.41	7.00E-03
11753890_a_at	MYST2	1.66	3.00E-03
11745181_a_at	NAA15	0.60	9.00E-04
11751697_a_at	NAA50	0.71	5.00E-03
11724221_at	NAP1L2	2.10	4.00E-03
11743505_a_at	NARS2	0.68	5.00E-03
11722211_a_at	NASP	0.59	1.00E-02
11754947_a_at	NAV1	0.68	6.00E-04
11723147_a_at	NAV3	2.31	1.00E-08
11754260_a_at	NBR2	1.59	1.00E-03
11720550_a_at	NCAPD3	0.63	5.00E-04
11722826_a_at	NCAPG	0.55	2.00E-03

11756784_x_at	NCAPG2	0.54	6.00E-05
11721854_a_at	NCAPH	0.52	6.00E-03
11725978_at	NCBP1	0.74	2.00E-04
11747323_x_at	NCBP2	0.78	8.00E-03
11745154_a_at	NCL	0.60	4.00E-06
11746005_a_at	NCRNA00085	0.60	3.00E-03
11744365_a_at	NCRNA00275	1.53	1.00E-02
11715509_a_at	NDFIP1	1.32	5.00E-03
11753431_x_at	NDRG1	1.84	9.00E-03
11741789_a_at	NDUFB6	0.72	3.00E-03
11715997_a_at	NDUFC1	0.76	6.00E-03
11722425_s_at	NEDD4L	3.71	1.00E-06
11725057_s_at	NEDD9	1.47	1.00E-02
11724821_a_at	NEK6	0.68	1.00E-03
11717451_at	NEK7	0.49	6.00E-03
11718576_s_at	NET1	0.65	5.00E-03
11722865_at	NETO2	0.45	7.00E-07
11715838_a_at	NEU1	1.92	3.00E-07
11719746_a_at	NF2	0.56	6.00E-03
11739078_x_at	NFE2L1	1.39	7.00E-04
11722661_at	NFE2L3	0.57	6.00E-03
11717220_at	NFIB	0.61	6.00E-04
11746913_x_at	NHEDC2	1.53	5.00E-03
11757725_a_at	NHP2	0.65	6.00E-05
11727849_a_at	NIP7	0.74	3.00E-03
11725627_at	NIPAL3	2.06	1.00E-08
11745029_a_at	NKRF	0.69	7.00E-03
11725117_a_at	NLN	0.73	4.00E-03
11717155_a_at	NME1 /// NME1-NME2	0.57	5.00E-07
11722197_at	NMT1	1.27	7.00E-04
11743018_at	NOC3L	0.66	5.00E-03
11722996_a_at	NOL11	0.74	3.00E-03
11725338_a_at	NOL12	0.76	9.00E-03
11715697_a_at	NOLC1	0.52	8.00E-08
11758780_a_at	NOP16	0.53	5.00E-06
11756849_x_at	NOP2	0.65	1.00E-03
11755342_x_at	NOP56	0.57	2.00E-05
11747090_a_at	NOP58	0.65	6.00E-04
11743916_a_at	NPC1	3.14	4.00E-10
11730790_x_at	NPM1	0.75	5.00E-03
11722213_at	NPM3	0.61	4.00E-04
11717567_a_at	NQO1	1.53	2.00E-03
11722087_a_at	NR1D2	1.77	5.00E-03
11744801_a_at	NR2C2AP	0.66	4.00E-03
11732248_at	NR2F1	0.29	1.00E-08
11727749_a_at	NR5A2	0.42	1.00E-03
11740346_a_at	NRG1	0.43	1.00E-05
11720153_s_at	NRIP1	0.55	3.00E-03
11720522_a_at	NSDHL	2.00	4.00E-07
11741390_x_at	NSFL1C	1.36	7.00E-03
11756424_a_at	NSMCE4A	0.76	1.00E-03
11755207_a_at	NT5E	1.31	5.00E-03
11739973_s_at	NUAK1	1.49	8.00E-03
11720088_x_at	NUCB2	2.14	5.00E-04
11728648_a_at	NUCKS1	0.68	5.00E-03
11744612_a_at	NUDCD1	0.57	4.00E-07
11758564_s_at	NUDT15	0.68	4.00E-03
11756899_a_at	NUDT21	0.70	1.00E-03
11755484_a_at	NUP107	0.70	8.00E-04
11737301_a_at	NUP155	0.63	4.00E-04
11736253_a_at	NUP160	0.71	5.00E-03
11755763_a_at	NUP188	0.74	2.00E-03

11717997_at	NUP205	0.69	3.00E-03
11751106_x_at	NUP35	0.59	2.00E-05
11758447_s_at	NUP50	0.71	7.00E-03
11743662_a_at	NUP54	0.69	3.00E-03
11754052_a_at	NUP85	0.65	4.00E-03
11722553_a_at	NUP88	0.69	3.00E-03
11754814_a_at	NUP93	0.68	6.00E-06
11720796_x_at	NUPL1	0.70	5.00E-03
11716937_a_at	NUPR1	4.41	1.00E-06
11742735_a_at	NUSAP1	0.53	4.00E-03
11758981_a_at	NUTF2	0.72	7.00E-03
11717320_a_at	OAF	1.85	2.00E-04
11723699_s_at	OAS3	0.47	3.00E-05
11732155_a_at	OGT	1.79	5.00E-04
11727543_at	OIP5	0.53	2.00E-03
11748908_a_at	ORAI2	1.31	9.00E-03
11744228_x_at	ORAI3	2.23	2.00E-08
11727837_at	ORC6	0.61	3.00E-04
11718990_x_at	OS9	1.39	8.00E-03
11755121_x_at	OSCP1	1.57	1.00E-03
11719882_s_at	OSTM1	1.43	6.00E-03
11743412_at	OTUD1	1.78	6.00E-03
11745194_a_at	OXCT1	0.67	4.00E-04
11748548_a_at	P2RX4	2.03	2.00E-03
11723412_s_at	PA2G4	0.55	2.00E-05
11718303_a_at	PAFAH1B3	0.71	3.00E-03
11720103_a_at	PAK1	0.62	2.00E-03
11728043_at	PANX2	3.94	3.00E-09
11745950_a_at	PARP1	0.55	8.00E-05
11757624_s_at	PARP12	0.60	2.00E-03
11731569_at	PAWR	0.63	5.00E-03
11723950_a_at	PBK	0.48	2.00E-03
11749105_a_at	PCDH1	1.64	9.00E-04
11759895_at	PCDH9	1.93	7.00E-04
11758060_s_at	PCGF5	0.66	2.00E-03
11719089_a_at	PCK2	2.73	6.00E-05
11716103_a_at	PCNA	0.46	3.00E-06
11763265_s_at	PCYT1A	1.39	6.00E-03
11717304_x_at	PDAP1	0.79	7.00E-03
11723985_a_at	PDCD11	0.73	5.00E-04
11722085_a_at	PDCD2L	0.70	2.00E-03
11720953_x_at	PDCD5	0.70	2.00E-03
11723484_x_at	PDCL3	0.72	1.00E-03
11737003_a_at	PDE3A	0.66	9.00E-03
11719056_s_at	PDE8A	1.49	1.00E-04
11751373_a_at	PDGFC	0.46	1.00E-03
11722855_at	PDGFD	2.35	7.00E-03
11743440_at	PDIA3	1.25	3.00E-03
11716118_a_at	PDIA4	1.65	1.00E-03
11745720_s_at	PDIA6	1.25	9.00E-03
11716974_a_at	PDK4	2.25	6.00E-03
11743904_s_at	PDLIM1	0.62	5.00E-03
11717107_a_at	PDLIM5	0.71	6.00E-03
11730905_a_at	PDS5B	0.76	5.00E-03
11720547_a_at	PDSS1	0.49	1.00E-07
11725762_a_at	PELI3	1.45	2.00E-04
11732482_a_at	PEX13	0.79	7.00E-03
11756227_s_at	PEX2	0.71	3.00E-03
11726711_x_at	PFAS	0.51	8.00E-06
11722406_at	PFKFB4	1.96	2.00E-03
11753674_a_at	PFN2	1.59	8.00E-03
11763354_a_at	PGAM5	0.59	4.00E-03

11755641_s_at	PGAP1	0.62	9.00E-05
11726504_at	PGBD3	0.52	4.00E-03
11715488_s_at	PGRMC1	1.36	5.00E-03
11720610_x_at	PHB	0.74	8.00E-03
11740230_a_at	PHEX	1.52	2.00E-03
11724070_a_at	PHF10	0.55	3.00E-04
11722880_a_at	PHF19	0.64	7.00E-04
11728203_a_at	PHGDH	4.76	3.00E-09
11737042_s_at	PHLDB2	0.55	5.00E-03
11719250_s_at	PHLPP1	0.63	3.00E-03
11727180_a_at	PHTF1	1.60	9.00E-03
11729216_at	PIGX	0.72	6.00E-03
11724361_s_at	PIKFYVE	1.33	9.00E-03
11739347_a_at	PIP4K2A	1.66	7.00E-04
11756270_x_at	PKP4	0.62	5.00E-03
11727754_a_at	PLA2G4A	2.97	3.00E-05
11743056_at	PLAA	1.47	3.00E-04
11720244_at	PLCG2	0.68	7.00E-03
11723110_at	PLEKHB2	1.31	2.00E-03
11717846_a_at	PLEKHJ1	0.77	5.00E-03
11722403_a_at	PLEKHO1	1.51	2.00E-04
11742745_a_at	PLIN2	1.84	9.00E-03
11722162_a_at	PLK2	0.49	6.00E-03
11721335_x_at	PLOD2	0.62	2.00E-04
11741315_a_at	PLSCR4	0.40	4.00E-04
11734479_at	PLXNA4	0.50	6.00E-06
11733425_at	PMCH	3.73	2.00E-05
11721699_a_at	PMS1	0.66	3.00E-04
11746348_x_at	PNN	0.70	5.00E-03
11729186_at	PNO1	0.65	3.00E-05
11718400_a_at	PNP	1.66	8.00E-08
11739766_at	PNPLA3	3.61	2.00E-06
11734725_a_at	PNPT1	0.67	5.00E-03
11715763_a_at	PODXL	1.59	7.00E-04
11726156_at	POLA1	0.52	8.00E-06
11721567_a_at	POLD1	0.78	9.00E-03
11733102_at	POLD3	0.65	6.00E-03
11744519_a_at	POLE2	0.35	2.00E-09
11763572_s_at	POLE3	0.64	1.00E-03
11722215_at	POLE4	0.69	3.00E-03
11729178_at	POLH	0.68	1.00E-02
11724544_a_at	POLR1A	0.70	2.00E-03
11755321_x_at	POLR1B	0.59	3.00E-05
11745730_a_at	POLR1E	0.66	1.00E-02
11722451_s_at	POLR2D	0.72	6.00E-04
11720324_a_at	POLR2F	0.79	4.00E-03
11758396_s_at	POLR3G	0.54	9.00E-05
11721868_a_at	POP5	0.67	9.00E-03
11716114_x_at	POR	1.29	7.00E-03
11715684_a_at	PPA1	0.66	7.00E-05
11741824_x_at	PPA2	0.71	5.00E-03
11754801_x_at	PPAP2A	1.89	1.00E-06
11756162_s_at	PPAP2B	2.85	2.00E-04
11744418_a_at	PPAPDC1B	2.03	4.00E-04
11743337_at	PPAPDC2	1.66	4.00E-04
11731900_s_at	PPAT	0.49	1.00E-06
11719383_x_at	PPIH	0.67	5.00E-03
11718036_at	PPIL1	0.66	7.00E-04
11744306_a_at	PPIL5	0.65	7.00E-04
11715575_x_at	PPM1G	0.81	8.00E-03
11755018_a_at	PPP1CC	0.70	6.00E-03
11744465_x_at	PPP1R15A	1.55	8.00E-04

11748282_a_at	PPP3CB	0.66	5.00E-04
11757779_s_at	PPP4R1	0.72	5.00E-04
11718606_a_at	PPPDE2	1.72	1.00E-07
11748712_a_at	PPRC1	0.64	1.00E-03
11742107_a_at	PPT1	0.76	6.00E-03
11755451_x_at	PQLC2	1.42	2.00E-03
11716358_s_at	PRC1	0.58	3.00E-03
11754018_a_at	PRDX3	0.77	8.00E-03
11728040_x_at	PRDX5	1.28	5.00E-03
11716849_s_at	PREX1	1.46	4.00E-03
11730261_a_at	PRKAR2B	0.60	7.00E-04
11722776_at	PRKD1	1.62	3.00E-05
11740423_a_at	PRKDC	0.65	7.00E-03
11715825_a_at	PRMT1	0.63	7.00E-05
11755951_s_at	PRMT5	0.77	3.00E-03
11745482_s_at	PRNP	2.00	3.00E-03
11737798_a_at	PROCR	0.78	7.00E-03
11743386_s_at	PRPF40A	0.76	3.00E-03
11757570_s_at	PRR15	0.36	2.00E-05
11718767_x_at	PRSS23	0.71	2.00E-03
11752510_x_at	PSAP	1.39	2.00E-03
11747349_s_at	PSAT1	3.89	3.00E-07
11745940_a_at	PSD3	0.58	6.00E-04
11742933_a_at	PSIP1	0.64	3.00E-03
11715668_a_at	PSMA5	1.37	1.00E-03
11754482_a_at	PSMB1	1.30	6.00E-03
200039_PM_s_at	PSMB2	1.48	6.00E-05
11754271_a_at	PSMB4	1.47	7.00E-04
11715595_a_at	PSMB7	1.57	3.00E-05
11734865_a_at	PSMC3IP	0.48	2.00E-07
11718881_a_at	PSMC4	1.58	1.00E-04
11743902_at	PSMD1	1.36	5.00E-03
11753142_a_at	PSMD11	1.44	1.00E-03
11719927_a_at	PSMD13	1.35	6.00E-03
11744362_a_at	PSMD14	1.42	4.00E-03
11715515_a_at	PSMD3	1.66	2.00E-06
11729131_a_at	PSME3	0.72	1.00E-04
11743499_a_at	PSMG4	0.64	5.00E-03
11742456_a_at	PTBP1	0.66	3.00E-05
11724441_x_at	PTGIS	0.16	1.00E-08
11736655_a_at	PTGR1	2.48	5.00E-06
11724037_at	PTGS2	3.43	9.00E-03
11715845_s_at	PTPRF	0.57	1.00E-04
11729976_a_at	PTPRN2	1.99	1.00E-04
11728787_a_at	PTPRR	3.56	1.00E-08
11730730_s_at	PTRF	0.68	8.00E-04
11717521_x_at	PTTG1 /// PTTG3P	0.58	2.00E-03
11755540_a_at	PUS7	0.59	5.00E-03
11718065_a_at	PVRL2	0.70	9.00E-04
11734644_a_at	PWWP2B	1.59	3.00E-04
11723314_x_at	PXMP2	0.62	6.00E-04
11722214_a_at	QTRT1	0.63	6.00E-03
11724089_a_at	RAB24	1.39	9.00E-04
11757731_s_at	RAB31	1.33	8.00E-03
11727877_a_at	RAB4B	1.53	3.00E-03
11716287_at	RABAC1	1.36	6.00E-04
11720808_a_at	RABEPK	0.69	3.00E-04
11734841_a_at	RAD51	0.59	1.00E-04
11743190_s_at	RAD51AP1	0.46	9.00E-05
11732520_a_at	RAD51C	0.70	7.00E-03
11728447_at	RAD54B	0.57	2.00E-03
11753308_s_at	RAET1G /// RAET1L /// ULBP2	1.96	1.00E-03

11726551_s_at	RAET1L /// ULBP2	2.00	4.00E-03
11745936_a_at	RAG1AP1	1.43	6.00E-03
11717758_x_at	RALA	1.42	7.00E-03
11728004_s_at	RALGPS2	0.58	1.00E-03
11753560_s_at	RAN	0.63	1.00E-04
11723001_s_at	RANBP1	0.51	1.00E-07
11757841_s_at	RANBP9	1.21	6.00E-03
11717164_a_at	RANGAP1	0.63	7.00E-04
11716435_a_at	RAPGEF1	1.53	9.00E-05
11722116_a_at	RARB	0.38	9.00E-04
11754224_x_at	RASA4	2.14	6.00E-03
11716839_a_at	RASD1	2.51	2.00E-05
11740140_a_at	RASGRP3	4.29	4.00E-03
11727547_s_at	RBBP8	0.62	1.00E-03
11744210_s_at	RBM14 /// RBM4	0.76	7.00E-03
11721679_a_at	RBM17	0.76	4.00E-03
11754999_x_at	RBM25	0.75	8.00E-03
11756968_a_at	RBM28	0.69	1.00E-02
11717423_s_at	RBM8A	0.69	4.00E-04
11761738_x_at	RBMX	0.63	3.00E-03
11741442_s_at	RBMX /// RBMXL1	0.59	6.00E-05
11732251_s_at	RCOR3	1.29	3.00E-03
11748807_a_at	RDH11	1.54	5.00E-03
11759658_a_at	RECK	0.58	2.00E-03
11748563_s_at	REEP5	1.39	2.00E-03
11744199_a_at	RETSAT	1.38	2.00E-04
11735167_x_at	RFC2	0.52	1.00E-04
11718403_at	RFC3	0.54	1.00E-03
11722872_a_at	RFC4	0.54	2.00E-03
11744232_a_at	RFC5	0.66	6.00E-03
11716046_a_at	RGL2	1.58	2.00E-04
11740106_a_at	RGS3	0.61	9.00E-04
11719551_s_at	RHOBTB1	1.57	3.00E-04
11758676_s_at	RHOQ	2.41	2.00E-06
11732625_a_at	RIMS1	0.57	7.00E-03
11725079_a_at	RIT1	1.41	9.00E-03
11718988_s_at	RNASE4	3.10	1.00E-06
11757017_x_at	RNASEH2A	0.60	2.00E-04
11734359_a_at	RNASEL	1.84	4.00E-04
11717807_at	RNF103	1.60	2.00E-06
11743013_a_at	RNF115	2.06	1.00E-07
11754399_a_at	RNF121	0.64	6.00E-03
11758506_s_at	RNF13	1.68	5.00E-04
11723232_x_at	RNF138	0.66	1.00E-03
11731751_at	RNF213	1.65	5.00E-03
11721954_s_at	RNF41	0.64	9.00E-04
11751858_a_at	RNF7	1.36	4.00E-04
200060_PM_s_at	RNPS1	0.73	4.00E-03
11716572_s_at	RPA1	0.66	6.00E-05
11724468_a_at	RPA3	0.73	3.00E-03
11743266_a_at	RPL34	0.73	7.00E-03
11759540_a_at	RPL35A	0.57	3.00E-07
11739074_x_at	RPL7L1	0.72	1.00E-03
11748113_a_at	RPN1	1.45	3.00E-03
11725715_x_at	RPS14	0.50	1.00E-07
11757128_at	RPSA /// SNORA62	0.51	9.00E-05
11719398_s_at	RRAGC	1.68	9.00E-03
11743604_s_at	RRM1	0.62	7.00E-04
11758219_x_at	RRM2	0.23	9.00E-07
11719810_at	RRS1	0.68	7.00E-05
11737848_a_at	RSPH3	1.43	3.00E-03
11724619_at	RSPO3	0.25	9.00E-06

11736204_a_at	RSU1	0.72	6.00E-03
11720304_s_at	RTN3	1.29	5.00E-03
11747775_x_at	RUVBL1	0.60	3.00E-05
11730398_a_at	RUVBL2	0.74	4.00E-04
11756438_a_at	SAFB	0.70	2.00E-03
11757764_s_at	SC4MOL	3.68	5.00E-07
11717950_s_at	SC5DL	2.68	4.00E-06
11715561_s_at	SCD	1.58	7.00E-03
11749550_a_at	SCPEP1	1.83	2.00E-05
11744785_x_at	SCRIB	0.66	1.00E-04
11746798_x_at	SDC2	0.60	9.00E-03
11717639_x_at	SDC4	0.56	8.00E-03
11716564_a_at	SDCCAG3	0.76	5.00E-03
11725694_at	SDPR	0.29	2.00E-03
11744264_a_at	SEC11C	2.45	3.00E-10
11749955_a_at	SEC61A1	1.65	4.00E-04
11734049_a_at	SEH1L	0.55	2.00E-05
11753915_a_at	SEL1L3	1.77	6.00E-03
11730390_at	SEMA3A	0.28	3.00E-05
11746511_s_at	SEPHS1	0.70	3.00E-03
11739942_s_at	SEPSECS	1.62	2.00E-03
11715806_a_at	SEPW1	0.72	3.00E-04
11718573_s_at	SERBP1	0.61	7.00E-05
11719267_s_at	SERINC1	1.41	2.00E-03
11747464_a_at	SERINC3	1.61	1.00E-05
11720309_s_at	SERP1	1.34	5.00E-03
11716376_at	SERPINA5	0.63	4.00E-05
11727056_s_at	SERPIND1	0.16	9.00E-04
11757442_a_at	SERPINE2	2.68	7.00E-06
11715769_a_at	SET	0.76	6.00E-04
11720474_a_at	SFPQ	0.68	1.00E-06
11756286_a_at	SFXN2	0.62	4.00E-03
11721102_a_at	SGSH	1.38	5.00E-04
11724816_a_at	SH3KBP1	1.33	9.00E-03
11733203_at	SH3TC2	1.61	4.00E-04
11723370_s_at	SHANK3	1.51	3.00E-03
11732256_at	SHB	0.68	1.00E-03
11754600_a_at	SHC3	0.45	4.00E-05
11728403_at	SHCBP1	0.53	7.00E-03
11756240_a_at	SHISA4	1.34	6.00E-04
11740366_a_at	SHMT1	0.52	2.00E-03
11750992_x_at	SHMT2	1.49	9.00E-03
11728887_x_at	SIP1	0.73	8.00E-03
11719727_a_at	SIRPA	1.64	3.00E-03
11729174_a_at	SKA2	0.62	8.00E-03
11729310_a_at	SKA3	0.55	5.00E-04
11720465_a_at	SKIV2L2	0.75	5.00E-03
11744273_a_at	SLBP	0.75	7.00E-03
11724134_a_at	SLC12A2	1.75	2.00E-03
11726809_x_at	SLC12A8	1.91	2.00E-03
11758156_s_at	SLC17A5	1.77	5.00E-06
11723149_a_at	SLC19A2	0.61	2.00E-03
11743477_at	SLC25A13	0.66	2.00E-04
11724064_a_at	SLC25A19	0.64	2.00E-03
11719829_a_at	SLC26A11	1.92	1.00E-06
11733413_a_at	SLC26A6	1.52	9.00E-03
11744517_a_at	SLC2A10	1.64	8.00E-04
11734657_s_at	SLC2A14 /// SLC2A3	1.72	9.00E-03
11733799_a_at	SLC31A2	1.62	1.00E-03
11746569_x_at	SLC35C2	1.36	6.00E-03
11723274_a_at	SLC35F2	0.59	2.00E-03
11720075_s_at	SLC35F5	1.82	7.00E-07

11743091_x_at	SLC36A4	1.49	8.00E-03
11735352_x_at	SLC37A2	1.69	5.00E-03
11744715_a_at	SLC38A6	2.57	5.00E-09
11756786_x_at	SLC38A9	1.38	1.00E-04
11733071_a_at	SLC3A2	2.14	3.00E-05
11719813_s_at	SLC48A1	1.69	7.00E-04
11720333_a_at	SLC5A6	0.67	3.00E-03
11721875_at	SLC7A11	2.28	3.00E-07
11743938_at	SLC7A14	0.44	1.00E-07
11727907_a_at	SLC9A3R2	1.66	8.00E-04
11757926_s_at	SLC9A9	2.69	2.00E-03
11718266_s_at	SMAD3	0.64	7.00E-03
11754590_x_at	SMARCA4	0.76	3.00E-03
11742782_at	SMARCC1	0.67	1.00E-03
11752770_a_at	SMARCE1	0.71	8.00E-04
11718879_s_at	SMC1A	0.70	4.00E-03
11724351_a_at	SMC6	0.71	7.00E-03
11723585_a_at	SMPDL3A	1.79	4.00E-06
11716139_s_at	SMS	0.64	5.00E-06
11723821_a_at	SMURF2	0.65	2.00E-03
11734793_at	SNAPC1	0.51	2.00E-05
11722036_a_at	SND1	1.35	7.00E-03
11757152_s_at	SNORA18 /// TAF1D	0.66	7.00E-04
11757119_at	SNORA6	0.62	1.00E-03
11720630_a_at	SNRNP25	0.63	2.00E-04
11754463_a_at	SNRNP70	0.70	4.00E-03
11720945_x_at	SNRPA1	0.60	4.00E-06
11757661_x_at	SNRPB	0.78	4.00E-03
11724203_at	SNRPD1	0.63	7.00E-04
11717431_a_at	SNRPD3	0.76	1.00E-03
11718040_x_at	SNRPE	0.70	2.00E-03
11717014_x_at	SNRPF	0.67	1.00E-04
11750607_a_at	SNX13	1.40	4.00E-03
11743589_at	SNX29	2.31	2.00E-05
11737753_s_at	SNX5	0.62	2.00E-04
11746327_a_at	SNX6	0.73	7.00E-03
11746543_a_at	SNX9	1.48	4.00E-03
11716935_a_at	SOD1	1.27	9.00E-03
11725743_s_at	SORBS1	1.44	7.00E-03
11743874_a_at	SOX17	1.46	1.00E-03
11718896_x_at	SPAG9	2.17	3.00E-08
11744886_a_at	SPAG9	2.03	1.00E-03
11723998_at	SPC25	0.36	2.00E-04
11721117_at	SPCS3	1.53	3.00E-03
11758490_s_at	SPG20	1.41	4.00E-04
11721122_x_at	SPNS1	1.33	3.00E-03
11721125_at	SPPL2A	1.40	3.00E-04
11754344_s_at	SPRY4	1.75	7.00E-03
11717729_a_at	SQLE	2.60	4.00E-09
11752843_x_at	SQSTM1	3.36	2.00E-06
11759548_a_at	SREBF2	1.48	4.00E-04
11747210_a_at	SRI	0.67	1.00E-02
11717377_s_at	SRPRB	1.41	4.00E-03
11745984_a_at	SRRM1	0.74	3.00E-03
11743269_a_at	SRSF1	0.64	1.00E-03
11718505_s_at	SRSF10	0.69	2.00E-03
11754191_x_at	SRSF11	0.73	2.00E-03
11749302_s_at	SRSF2	0.67	5.00E-06
11717293_x_at	SRSF7	0.62	1.00E-06
11718665_s_at	SRSF9	0.77	4.00E-03
11716950_s_at	SRXN1	2.43	1.00E-07
11750014_x_at	SSBP1	0.79	2.00E-03

11754319_at	SSH1	1.53	5.00E-03
11734068_a_at	SSR3	1.39	8.00E-03
11715748_at	SSRP1	0.64	1.00E-03
11743839_a_at	ST3GAL6	1.93	4.00E-03
11734317_a_at	STARD8	2.07	6.00E-07
11741400_a_at	STMN1	0.46	2.00E-04
11716055_s_at	STMN3	0.54	8.00E-03
11716606_s_at	STOML2	0.74	3.00E-04
11725944_a_at	STT3A	1.41	3.00E-03
11727994_at	STX11	0.39	7.00E-03
11719534_a_at	STX3	1.60	2.00E-03
11729699_a_at	STXBP1	1.34	3.00E-03
11758245_s_at	SUB1	0.69	6.00E-04
11755266_x_at	SUCLA2	0.75	4.00E-03
11717114_a_at	SUPT16H	0.70	3.00E-05
11715831_s_at	SURF4	1.45	1.00E-03
11734831_at	SUV39H2	0.74	6.00E-03
11721430_a_at	SYBU	1.99	9.00E-03
11720666_at	SYNCRIP	0.79	9.00E-03
11755757_a_at	SYNM	3.92	1.00E-08
11757050_x_at	TAGLN2	0.69	8.00E-03
11715447_at	TALDO1	1.71	5.00E-07
11755810_s_at	TAPT1	1.68	6.00E-07
11727377_a_at	TBC1D15	1.56	5.00E-03
11752602_a_at	TBC1D17	1.37	7.00E-03
11728034_a_at	TBC1D7	1.67	2.00E-03
11723525_at	TBC1D8	2.58	1.00E-08
11752579_x_at	TBXA2R	1.49	6.00E-03
11740678_a_at	TBXAS1	1.88	7.00E-03
11721140_a_at	TCEA3	1.69	3.00E-03
11715202_s_at	TCEAL1	1.29	3.00E-03
11740674_a_at	TCERG1	0.63	1.00E-04
11724026_a_at	TCF19	0.47	9.00E-05
11717959_a_at	TCF3	0.68	2.00E-03
11725064_x_at	TCOF1	0.72	4.00E-03
11737373_at	TCP11L2	1.67	3.00E-03
11754822_x_at	TDP1	0.69	6.00E-04
11727852_x_at	TEX264	1.45	1.00E-04
11743811_a_at	TFAM	0.59	3.00E-03
11725349_s_at	TFDP1	0.66	2.00E-07
11732084_a_at	TFEC	3.39	6.00E-05
11740596_a_at	TGFB2	0.23	4.00E-07
11739781_a_at	TGM2	0.32	3.00E-06
11754439_x_at	TH1L	0.70	2.00E-03
11754632_a_at	THAP8	1.74	4.00E-04
11723682_at	THOC4	0.64	4.00E-06
11719380_a_at	THOC7	0.74	3.00E-03
11755929_a_at	THOP1	0.64	1.00E-03
11744117_at	THSD7A	0.59	7.00E-03
11736572_at	TIGD6	1.40	4.00E-04
11719123_a_at	TIMELESS	0.59	7.00E-05
11754015_a_at	TIMM50	0.74	9.00E-03
11715868_a_at	TINF2	1.31	7.00E-03
11761622_x_at	TIPIN	0.68	3.00E-03
11718127_at	TK1	0.45	2.00E-06
11727557_s_at	TK2	1.42	9.00E-04
11721680_a_at	TM2D1	1.42	2.00E-03
11753131_x_at	TM4SF1	1.60	4.00E-03
11725403_at	TM4SF18	3.05	5.00E-05
11728884_a_at	TM6SF1	1.54	3.00E-03
11752841_x_at	TM7SF2	1.29	2.00E-03
11747326_a_at	TM9SF1	1.35	9.00E-03

11759506_a_at	TM9SF2	1.78	2.00E-04
11718408_x_at	TMBIM6	1.29	3.00E-03
11740736_x_at	TMC7	1.71	6.00E-05
11721006_s_at	TMCO3	1.40	1.00E-03
11715544_a_at	TMED10	1.39	1.00E-03
200087_PM_s_at	TMED2	1.27	2.00E-03
11751524_x_at	TMED5	1.36	7.00E-03
11725312_a_at	TMED7	1.53	5.00E-03
11744110_at	TMEM101	1.44	5.00E-03
11716729_a_at	TMEM111	1.69	6.00E-07
11718421_at	TMEM115	1.29	8.00E-03
11725498_a_at	TMEM117	2.01	2.00E-05
11746670_a_at	TMEM120A	1.58	6.00E-05
11752036_s_at	TMEM123	0.76	2.00E-03
11724248_s_at	TMEM127	1.47	7.00E-03
11758500_s_at	TMEM140	4.56	2.00E-06
11731602_at	TMEM154	2.48	1.00E-05
11719110_a_at	TMEM161A	1.39	3.00E-03
11727044_a_at	TMEM173	2.46	5.00E-06
11755308_x_at	TMEM189	1.37	3.00E-03
11724739_s_at	TMEM204	2.11	2.00E-05
11732105_at	TMEM208	1.55	4.00E-04
11727860_at	TMEM209	1.55	9.00E-04
11724122_a_at	TMEM22	1.91	3.00E-05
11722756_at	TMEM37	1.80	3.00E-03
11758119_s_at	TMEM41B	1.72	5.00E-03
11721758_at	TMEM47	1.35	9.00E-03
11752409_a_at	TMEM48	0.71	5.00E-03
11744587_a_at	TMEM57	1.55	8.00E-04
11745879_a_at	TMEM66	1.65	7.00E-04
11724642_at	TMEM88	0.24	1.00E-04
11758848_at	TMEM97	1.51	8.00E-03
11731918_a_at	TMPO	0.52	5.00E-04
11720240_at	TMSB15A	0.46	8.00E-05
11726136_a_at	TMSB15B	0.57	5.00E-03
11732011_at	TNFAIP8L3	2.57	5.00E-08
11753764_s_at	TNFRSF21	0.49	1.00E-03
11739878_at	TNIK	0.58	3.00E-03
11756811_x_at	TOM1	1.72	3.00E-03
11716107_a_at	TOMM22	0.75	2.00E-03
11753887_x_at	TOMM5	0.78	3.00E-03
11717438_x_at	TOP1	0.72	3.00E-05
11723612_a_at	TOX2	2.91	6.00E-06
11716399_at	TP53INP2	1.62	3.00E-04
11721486_at	TP53RK	0.74	4.00E-03
11761349_x_at	TPM1	0.52	8.00E-07
11748342_x_at	TPP1	1.54	4.00E-03
11744698_x_at	TPRG1L	1.36	6.00E-03
11750598_s_at	TPX2	0.58	5.00E-03
11746041_a_at	TRA2B	0.67	4.00E-03
11725373_a_at	TRADD	1.47	5.00E-04
11716148_a_at	TRAF3IP2	1.52	9.00E-04
11720285_s_at	TRAK1	1.41	3.00E-03
11716784_at	TRAK2	1.34	2.00E-04
11757983_s_at	TRAM1	1.71	9.00E-07
11715814_a_at	TRAP1	0.62	1.00E-04
11753721_s_at	TRAPPC2 /// TRAPPC2P1	1.36	1.00E-03
11718280_s_at	TRIAP1	0.76	7.00E-03
11716940_a_at	TRIB2	0.59	3.00E-04
11728668_x_at	TRIM16	2.01	1.00E-03
11749777_s_at	TRIM16 /// TRIM16L	2.10	5.00E-03
11722862_x_at	TRIM2	2.99	2.00E-08

11721085_a_at	TRIM37	0.74	2.00E-03
11759631_at	TRIO	1.42	2.00E-03
11731526_s_at	TRIP13	0.51	2.00E-05
11728825_at	TRNP1	2.06	2.00E-03
11719877_a_at	TRPT1	0.75	5.00E-03
11751415_a_at	TSC22D3	1.99	3.00E-04
11754940_s_at	TSN	0.77	1.00E-03
11732142_at	TSR1	0.61	2.00E-05
11759667_a_at	TTC17	1.59	5.00E-04
11718142_a_at	TTC27	0.69	8.00E-04
11728326_a_at	TTC30A	1.78	2.00E-05
11739863_a_at	TTF2	0.68	8.00E-04
11743676_a_at	TTLL7	1.53	3.00E-03
11756979_x_at	TUBA1B	0.69	3.00E-04
11756670_x_at	TUBA1C	0.66	4.00E-05
11716460_x_at	TUBA4A	2.27	6.00E-04
11750415_x_at	TUBB2C	0.67	2.00E-04
11715791_at	TUBG1	0.67	2.00E-04
11755462_a_at	TUBGCP4	0.71	3.00E-04
11757541_x_at	TXNDC17	0.53	5.00E-06
11756431_s_at	TXNIP	1.68	1.00E-04
11715649_s_at	TXNRD1	1.31	1.00E-03
11751805_a_at	TYMS	0.24	4.00E-03
11719966_x_at	U2AF1	0.72	1.00E-03
11729317_a_at	UACA	1.78	6.00E-03
11724535_a_at	UBA5	0.75	5.00E-03
11724328_a_at	UBE2C	0.57	8.00E-03
11742690_a_at	UBE2I	0.77	5.00E-03
11758226_s_at	UBE2J1	1.67	2.00E-04
11718483_s_at	UBE2N	0.67	9.00E-04
11722845_s_at	UBE2R2	1.28	4.00E-03
11720501_a_at	UBE4B	1.48	2.00E-03
11736925_s_at	UBP1	0.76	2.00E-03
11725737_s_at	UBR3	1.46	6.00E-04
11719284_a_at	UBR7	0.72	5.00E-03
11758680_s_at	UCHL1	1.95	8.00E-06
11725084_a_at	UCHL5	0.70	2.00E-03
11727063_a_at	UCK2	0.52	5.00E-08
11725385_at	UGCG	0.51	5.00E-04
11723192_a_at	UHRF1	0.21	3.00E-14
11726552_x_at	ULBP2	1.91	8.00E-03
11756373_a_at	UMPS	0.71	4.00E-04
11747253_a_at	UNG	0.48	2.00E-04
11752238_x_at	UNKL	1.97	7.00E-07
11734018_at	URB1	0.71	6.00E-04
11746690_a_at	URB2	0.66	4.00E-03
11727789_a_at	USP1	0.57	9.00E-03
11717777_a_at	USP15	0.72	7.00E-03
11754638_a_at	USP30	1.54	6.00E-04
11731664_at	UTP20	0.62	2.00E-05
11743256_s_at	VAPA	0.75	2.00E-03
11757769_s_at	VCL	0.63	2.00E-04
11715655_s_at	VDAC1	0.77	2.00E-04
11754497_a_at	VIPAR	1.26	4.00E-03
11721181_a_at	VLDLR	2.51	9.00E-05
11721173_a_at	VPS8	1.33	8.00E-03
11723562_a_at	VWA5A	1.57	6.00E-04
11757981_s_at	WASF3	0.73	1.00E-03
11753429_a_at	WBP2	1.67	5.00E-03
11725709_a_at	WDHD1	0.68	6.00E-04
11746789_x_at	WDR12	0.71	4.00E-04
11720158_a_at	WDR19	1.46	3.00E-03

11742734_s_at	WDR3	0.65	9.00E-03
11723738_a_at	WDR36	0.66	1.00E-03
11739322_a_at	WDR4	0.62	9.00E-03
11727834_at	WDR43	0.63	3.00E-03
11728227_a_at	WDR45	1.78	2.00E-05
11717309_x_at	WDR75	0.79	4.00E-03
11746102_a_at	WDR76	0.57	7.00E-03
11716081_a_at	WDR77	0.72	8.00E-03
11719047_s_at	WEE1	0.63	3.00E-04
11735832_a_at	WHSC1	0.61	5.00E-04
11747808_a_at	WIPI1	1.67	2.00E-03
11733513_a_at	WLS	1.72	1.00E-06
11732584_a_at	WRN	0.70	9.00E-04
11748586_x_at	XPO1	0.81	1.00E-02
11720312_at	XRCC5	0.68	5.00E-05
11746472_a_at	XRN2	0.80	5.00E-03
11719415_a_at	YES1	0.69	3.00E-04
11721355_a_at	YPEL3	2.11	1.00E-03
11717797_at	ZER1	1.39	1.00E-03
11750575_a_at	ZFPM2	0.45	4.00E-03
11754428_a_at	ZFYVE26	1.27	3.00E-03
11760321_at	ZMYM6	2.03	1.00E-05
11728865_at	ZMYND19	0.70	5.00E-04
11743149_at	ZNF22	0.75	9.00E-03
11721601_s_at	ZNF238	0.67	3.00E-03
11718789_x_at	ZNF302	1.32	4.00E-03
11728101_at	ZNF367	0.40	1.00E-08
11732682_at	ZNF471	1.37	5.00E-03
11759097_a_at	ZNF703	0.63	5.00E-03
11721827_a_at	ZWILCH	0.57	3.00E-05
11740253_a_at	ZWINT	0.46	8.00E-05

Supplementary Table S2. Genes differentially expressed in human microvascular endothelial cells (HMVECs) in response to treatment with reconstituted high-density lipoproteins (rHDL) in inflammation. Fold changes and *P*-values are shown relative to HMVECs treated with phosphate-buffered saline (PBS) alone prior to inflammatory stimulation.

Probe ID	Gene	Fold Change	*P*-value
11716636_a_at	AARS	1.40	4.00E-03
11739503_at	ABCA1	0.30	3.00E-07
11730520_a_at	ABCA9	0.56	7.00E-03
11759048_at	ABCB1	0.54	9.00E-03
11724634_s_at	ABCB10	0.74	7.00E-04
11716187_a_at	ABCB6 /// ATG9A	1.88	9.00E-03
11743804_at	ABCE1	0.67	1.00E-03
11722025_x_at	ABCF2	0.71	3.00E-03
11725516_s_at	ABCG1	0.13	4.00E-06
11746269_a_at	ABHD3	2.38	8.00E-04
11718255_at	ABHD4	2.07	3.00E-05
11727020_at	ABI2	0.68	2.00E-03
11744255_a_at	ACADVL	1.61	6.00E-03
11746009_a_at	ACAT2	2.07	3.00E-05
11751740_a_at	ACER3	0.56	3.00E-03
11743758_a_at	ACO1	0.69	9.00E-03
11718647_at	ACVR1	1.49	6.00E-03
11731109_a_at	ADAM17	2.22	4.00E-04
11755797_x_at	ADAM9	0.75	5.00E-03
11730923_x_at	ADAMTS4	2.17	4.00E-03
11741720_s_at	ADAR	0.68	7.00E-05
11716514_s_at	ADD1	0.76	4.00E-03
11746331_a_at	ADK	1.71	2.00E-03
11730578_at	ADM2	2.30	2.00E-03
11716498_s_at	ADRM1	1.55	9.00E-05
11724877_a_at	AEBP2	0.64	3.00E-03
11754441_a_at	AGAP3	1.54	8.00E-04
11753687_x_at	AGTRAP	1.30	9.00E-03
11751921_s_at	AHR	1.75	9.00E-03
11723707_a_at	AIFM2	1.99	2.00E-03
11716563_s_at	AIMP2	0.65	2.00E-03
11747276_a_at	AKAP1	0.67	1.00E-04
11723804_a_at	AKAP12	1.99	4.00E-04
11750884_a_at	AKAP8L	1.57	2.00E-03
11724490_x_at	AKR1B10	2.53	2.00E-03
11719172_x_at	AKR1C1	3.97	3.00E-04
11754189_s_at	AKR1C1 /// AKR1C2	2.83	5.00E-03
11715711_a_at	AKR1C3	1.62	3.00E-04
11752549_x_at	ALDH1A1	0.33	7.00E-04
11752503_x_at	ALDH7A1	0.64	1.00E-03
11722488_a_at	ALG11 /// UTP14C	1.49	7.00E-03
11757951_s_at	ALG2	1.60	2.00E-03
11757588_a_at	ALG5	1.51	5.00E-03
11720274_x_at	ALKBH6	1.42	2.00E-03
11730691_s_at	ALS2	1.60	2.00E-03
11727576_x_at	ALS2CR4	0.55	1.00E-04
11758768_x_at	AMD1	0.68	1.00E-02
11741184_a_at	AMPD3	2.23	1.00E-04
11757527_a_at	ANAPC7	0.67	2.00E-04
11721569_a_at	ANG	3.34	9.00E-07
11722525_a_at	ANKHD1 /// ANKHD1-EIF4EBP3	1.65	2.00E-04
11727065_s_at	ANKRD11	1.69	4.00E-04
11748347_a_at	ANKRD28	2.91	1.00E-03
11742909_at	ANKRD50	0.55	2.00E-03
11724465_a_at	ANLN	0.41	9.00E-04

11717410_a_at	ANO6	1.62	2.00E-04
11739079_a_at	ANP32A	0.57	2.00E-03
11723643_a_at	ANXA7	1.39	4.00E-03
11719535_a_at	AP3M2	1.71	4.00E-03
11740603_a_at	APH1B	1.48	8.00E-03
11740303_a_at	APITD1	0.69	8.00E-03
11750223_a_at	APOA1BP	0.71	9.00E-03
11732901_a_at	APOBEC3A /// APOBEC3B	0.34	3.00E-04
11758898_x_at	APOBEC3C	0.67	1.00E-03
11741257_x_at	APOBEC3F	0.65	9.00E-03
11720510_a_at	APOBEC3G	0.66	3.00E-03
11723975_x_at	APOL3	0.51	1.00E-02
11715429_s_at	ARF4	1.31	3.00E-03
11718746_x_at	ARHGAP21	1.65	8.00E-04
11715466_at	ARHGDIB	0.60	3.00E-03
11721646_s_at	ARL1	1.47	8.00E-03
11729916_s_at	ARL5B	1.59	1.00E-03
11749803_s_at	ARL8B	1.45	4.00E-03
11744690_a_at	ARMC10	0.73	8.00E-04
11733928_a_at	ARMC8	0.57	3.00E-04
11737781_a_at	ARMCX2	0.57	5.00E-03
11755151_a_at	ARNTL	1.60	6.00E-03
11746862_x_at	ARSJ	0.49	5.00E-03
11716365_x_at	ASNS	8.28	1.00E-12
11725722_at	ATAD2	0.45	1.00E-04
11739868_a_at	ATF2	1.53	9.00E-03
11753631_a_at	ATF3	8.22	2.00E-05
11757047_x_at	ATF4	2.46	2.00E-06
11755593_a_at	ATG13	1.47	9.00E-03
11725488_a_at	ATG14	1.41	7.00E-04
11715755_a_at	ATIC	0.51	2.00E-05
11740332_a_at	ATP2A2	1.75	2.00E-03
11754003_x_at	ATP5J2	0.76	8.00E-03
11754478_x_at	ATP6V0B	1.62	2.00E-05
11716072_s_at	ATP6V1B2	1.73	1.00E-04
11741370_a_at	ATP6V1E1	1.36	6.00E-03
11754005_x_at	ATP6V1G1	1.36	1.00E-03
11715526_s_at	ATP6V1G2 /// BAT1	0.69	5.00E-03
11717264_a_at	ATPAF1	0.72	6.00E-03
11758202_s_at	ATPIF1	0.58	5.00E-04
11720203_a_at	AURKB	0.54	8.00E-03
11755585_a_at	AZI2	1.54	3.00E-03
11759495_at	BAG4	0.75	5.00E-03
11756612_a_at	BARD1	0.43	2.00E-04
11720443_s_at	BAZ1A	0.73	1.00E-04
11727881_at	BBS12	1.58	4.00E-03
11749895_a_at	BCAT1	1.59	1.00E-02
11727544_a_at	BCAT2	0.71	7.00E-03
11743504_at	BCCIP	0.61	2.00E-03
11742809_s_at	BCL2L13	1.27	8.00E-03
11759322_at	BCL6	2.04	3.00E-04
11724716_a_at	BCOR	2.48	4.00E-03
11758566_s_at	BCS1L	0.73	6.00E-03
11735902_a_at	BDNF	0.32	3.00E-03
11733864_a_at	BIRC5	0.51	7.00E-04
11756756_a_at	BLVRB	2.19	5.00E-06
11726120_a_at	BMP2K	1.88	2.00E-04
11734013_a_at	BMP4	0.25	2.00E-07
11758476_s_at	BNC2	0.57	3.00E-03
11743084_s_at	BNIP3	1.32	3.00E-03
11745828_x_at	BNIP3L	1.60	3.00E-05
11757102_a_at	BOP1	0.60	1.00E-04

11756939_s_at	BRD7	0.72	3.00E-04
11743005_s_at	BRF2	1.91	6.00E-05
11719169_s_at	BRI3	1.35	2.00E-03
11748918_a_at	BRIX1	0.61	1.00E-03
11729846_a_at	BST1	0.49	7.00E-03
11716036_x_at	BST2	0.44	4.00E-03
11733023_s_at	BTG1	2.71	3.00E-04
11717444_at	BYSL	0.56	1.00E-05
11748735_s_at	BZW1	0.73	7.00E-03
11747678_x_at	BZW2	0.67	2.00E-03
11717648_x_at	C10orf119	0.73	6.00E-03
11740731_x_at	C10orf125	0.73	3.00E-03
11728935_at	C11orf41	0.48	2.00E-03
11718063_a_at	C11orf48	0.69	5.00E-03
11754779_a_at	C11orf61	0.67	4.00E-03
11717046_at	C11orf95	0.67	5.00E-04
11719899_at	C11orf96	5.82	4.00E-03
11721899_x_at	C12orf24	0.42	6.00E-06
11726112_at	C12orf26	0.72	9.00E-03
11723719_at	C12orf45	0.72	7.00E-03
11740402_at	C12orf69	0.62	2.00E-03
11757616_s_at	C14orf1	1.80	5.00E-05
11719419_a_at	C14orf109	1.69	1.00E-03
11728044_a_at	C14orf126	0.69	3.00E-03
11754198_at	C14orf139	5.39	2.00E-10
11716270_a_at	C14orf156	0.75	9.00E-04
11736067_a_at	C15orf63 /// SERF2	0.72	5.00E-03
11717150_s_at	C16orf57	0.67	2.00E-03
11755095_a_at	C16orf80	0.76	2.00E-03
11723539_a_at	C16orf88	0.64	5.00E-03
11735553_a_at	C16orf93	1.45	1.00E-02
11742703_x_at	C17orf89	0.75	5.00E-03
11736304_a_at	C17orf91	1.57	7.00E-04
11741392_a_at	C18orf34	0.51	2.00E-03
11757284_x_at	C19orf10	1.39	4.00E-03
11726618_a_at	C1GALT1C1	0.70	9.00E-03
11724318_a_at	C1orf103	1.61	4.00E-03
11742483_a_at	C1orf110	0.17	4.00E-08
11718237_a_at	C1orf115	0.55	4.00E-03
11749341_a_at	C1orf131	0.64	4.00E-03
11741161_a_at	C1orf26	1.61	2.00E-04
11722431_a_at	C1orf27	1.49	4.00E-03
11719756_s_at	C1orf31	1.39	6.00E-03
11743322_at	C20orf111	2.01	7.00E-05
11749774_x_at	C21orf7	2.46	1.00E-06
11731505_a_at	C22orf39	0.58	1.00E-04
11764071_s_at	C2orf3	0.54	1.00E-03
11741631_a_at	C3orf58	2.04	5.00E-06
11722936_at	C3orf59	0.45	8.00E-05
11740430_x_at	C3orf63	0.72	6.00E-03
11730176_x_at	C4orf3	0.71	3.00E-03
11743405_a_at	C4orf34	2.03	9.00E-05
11727935_at	C4orf49	0.19	5.00E-03
11716579_a_at	C5orf32	1.52	1.00E-03
11756876_a_at	C6orf125	0.75	6.00E-03
11718540_at	C7orf23	2.13	6.00E-07
11722514_a_at	C9orf103	0.52	5.00E-03
11720515_s_at	C9orf150	2.04	7.00E-04
11720531_at	C9orf72	3.16	2.00E-05
11758550_x_at	C9orf91	1.71	8.00E-03
11743565_at	CABLES2	1.57	7.00E-03
11744395_s_at	CALCOCO2	1.41	8.00E-03

11730275_at	CALCRL	3.10	1.00E-05
11754149_s_at	CALM2	0.76	7.00E-03
11715374_a_at	CALR	2.20	7.00E-03
11717268_a_at	CAMK2G	1.49	5.00E-05
11727426_a_at	CANX	1.52	2.00E-04
11720456_at	CAPRIN1	0.63	6.00E-04
11737757_a_at	CARS	1.71	2.00E-03
11716843_a_at	CASC4	1.42	2.00E-04
11732870_a_at	CASP1	0.52	6.00E-03
11745716_s_at	CAV1	0.73	2.00E-03
11744286_s_at	CBS	2.06	3.00E-03
11715546_s_at	CBX1	0.65	3.00E-03
11716277_a_at	CBX5	0.49	5.00E-03
11722927_at	CCDC137	0.69	1.00E-03
11745072_a_at	CCDC149	1.69	2.00E-04
11721904_a_at	CCDC28B	0.66	3.00E-03
11744318_at	CCDC85A	0.27	5.00E-04
11756681_a_at	CCDC86	0.61	6.00E-05
11722572_at	CCNA2	0.44	1.00E-02
11716701_a_at	CCNDBP1	1.39	9.00E-03
11728300_at	CCNE2	0.54	2.00E-03
11726624_a_at	CCNG2	2.07	2.00E-03
11750355_a_at	CCPG1	3.10	1.00E-05
11748896_s_at	CCRL1	0.45	7.00E-03
11729424_s_at	CCRL2	3.51	1.00E-04
11715421_a_at	CCT2	0.73	4.00E-04
11750433_x_at	CCT3	0.76	2.00E-03
11747067_a_at	CCT5	0.76	7.00E-03
11728316_at	CD274	0.65	4.00E-03
11723691_at	CD302	0.57	3.00E-03
11739615_at	CD3EAP	0.60	3.00E-03
11743903_a_at	CD46	1.38	4.00E-03
11752832_x_at	CD55	1.61	2.00E-03
11758570_s_at	CDADC1	1.45	5.00E-03
11717163_s_at	CDC20	0.48	1.00E-03
11726756_a_at	CDC25A	0.54	9.00E-03
11720499_at	CDC42EP3	0.61	3.00E-03
11755012_a_at	CDC42SE2	0.76	6.00E-03
11732058_a_at	CDC7	0.48	6.00E-03
11719598_s_at	CDCA3	0.53	6.00E-04
11758478_s_at	CDCA7	0.25	1.00E-08
11717436_a_at	CDCA7L	0.61	7.00E-03
11742873_a_at	CDH11	0.44	1.00E-03
11753760_s_at	CDK2AP2	2.39	2.00E-07
11758661_s_at	CDKN2C	0.55	5.00E-03
11753788_x_at	CDKN3	0.55	6.00E-03
11722949_at	CDRT4	2.50	2.00E-05
11743077_s_at	CEBPB	3.01	3.00E-04
11717693_at	CEBPG	2.27	4.00E-04
11723465_a_at	CELF2	0.67	7.00E-03
11725231_a_at	CEP290	1.56	7.00E-03
11719053_s_at	CEP350	1.39	3.00E-03
11743543_x_at	CEP57	1.77	7.00E-03
11717116_at	CERK	1.55	5.00E-04
11720016_at	CGNL1	0.50	1.00E-03
11722407_a_at	CHAC1	2.48	1.00E-05
11728808_at	CHAC2	0.44	8.00E-06
11743284_at	CHD2	1.52	2.00E-03
11754263_s_at	CHD9	1.53	2.00E-03
11728758_at	CHIC2	1.37	7.00E-03
11736286_a_at	CHKA	1.83	2.00E-03
11731477_at	CHMP1B	1.44	6.00E-03

11741135_s_at	CHN1	0.57	3.00E-03
11717511_a_at	CIRH1A	0.67	5.00E-03
11721656_s_at	CISD2	1.77	3.00E-04
11724948_at	CITED4	0.61	3.00E-04
11753856_a_at	CKS2	0.66	2.00E-03
11743022_a_at	CLCC1	1.48	1.00E-02
11742798_a_at	CLCN3	1.74	6.00E-03
11729621_at	CLCN4	0.44	1.00E-09
11720434_at	CLCN6	1.84	1.00E-04
11728232_a_at	CLDN1	0.31	5.00E-03
11747704_a_at	CLDN11	0.49	2.00E-05
11735525_a_at	CLDN14	3.10	4.00E-03
11728603_a_at	CLDN23	1.51	9.00E-03
11747597_x_at	CLEC2D	0.30	1.00E-05
11727412_a_at	CLN8	1.64	2.00E-03
11754644_x_at	CNN2	0.63	6.00E-05
11755036_a_at	CNP	0.63	2.00E-04
11758133_s_at	COL4A3BP	1.61	4.00E-03
11739134_a_at	COL5A2	0.68	8.00E-03
11757481_a_at	COPS6	0.73	1.00E-03
11730823_at	COX19	1.67	7.00E-03
11748834_a_at	CRBN	1.52	1.00E-03
11758876_at	CREBRF	2.10	4.00E-04
11719327_a_at	CRELD2	2.39	4.00E-07
11736310_x_at	CSNK1A1	1.48	1.00E-03
11722981_a_at	CSPG5	0.44	1.00E-03
11730216_x_at	CSRP1	0.42	1.00E-05
11729239_x_at	CSRP2	0.59	9.00E-03
11723798_a_at	CSTF3	0.59	2.00E-05
11731874_x_at	CTAGE5	1.83	7.00E-05
11715441_a_at	CTGF	0.63	4.00E-03
11742278_a_at	CTH	4.38	6.00E-05
11720494_a_at	CTNNAL1	0.56	6.00E-03
11749064_x_at	CTNS	1.64	3.00E-03
11750141_a_at	CTPS	0.65	2.00E-04
11734552_a_at	CX3CL1	0.29	2.00E-07
11720298_at	CXCL10	0.52	5.00E-03
11728716_x_at	CXCL5	0.44	1.00E-03
11757901_x_at	CXXC5	1.80	5.00E-03
11748576_a_at	CYB5R1	1.75	7.00E-04
11717676_a_at	CYFIP2	0.55	8.00E-03
11722563_x_at	CYP51A1	2.60	2.00E-03
11738202_x_at	DAXX	0.75	5.00E-03
11729702_a_at	DBN1	0.70	5.00E-03
11730327_s_at	DCAF13	0.69	3.00E-03
11743061_at	DCTPP1	0.67	3.00E-05
11758198_s_at	DDIT3	9.19	1.00E-04
11743972_a_at	DDIT4	3.25	6.00E-07
11724796_at	DDIT4L	2.79	9.00E-04
11723859_a_at	DDR2	2.66	2.00E-04
11718995_at	DDX20	0.76	6.00E-03
11733073_a_at	DDX21	0.71	3.00E-03
11750684_a_at	DDX31	0.69	6.00E-03
11715849_a_at	DDX47	0.62	6.00E-06
11757922_s_at	DDX52	0.72	6.00E-03
11733149_a_at	DDX58	0.62	6.00E-03
11744236_a_at	DDX60	0.54	6.00E-03
11721114_a_at	DERA	0.72	8.00E-03
11717553_at	DERL2	1.78	4.00E-03
11726261_at	DFFA	0.63	2.00E-03
11721069_a_at	DGKD	1.91	5.00E-05
11715460_a_at	DHCR24	2.25	9.00E-07

11743808_a_at	DHCR7	2.07	4.00E-04
11749344_a_at	DHRS3	0.58	4.00E-04
11744862_x_at	DHRS7	1.39	4.00E-03
11727950_s_at	DHX33	0.69	2.00E-03
11722339_a_at	DIAPH2	1.97	3.00E-05
11743467_at	DKC1	0.68	5.00E-03
11744730_s_at	DLEU2 /// DLEU2L	0.39	7.00E-03
11744793_x_at	DLGAP5	0.51	9.00E-03
11724711_a_at	DMXL1	2.04	2.00E-04
11723330_a_at	DNAJB12	1.43	2.00E-03
11757735_s_at	DNAJB9	4.50	3.00E-04
11742906_a_at	DNAJC1	1.45	6.00E-04
11752102_s_at	DNAJC10	1.75	1.00E-04
11734719_a_at	DNAJC14	0.72	3.00E-03
11717373_at	DNAJC3	2.79	3.00E-03
11763164_at	DNAJC9 /// MRPS16	0.67	2.00E-03
11724448_a_at	DNMBP	0.72	2.00E-03
11756434_a_at	DNMT1	0.53	8.00E-06
11719941_s_at	DSCR3	1.41	2.00E-03
11727219_a_at	DSE	2.30	9.00E-05
11717562_x_at	DTX3L	0.56	8.00E-03
11716642_s_at	DTYMK	0.70	3.00E-03
11727383_a_at	DYM	1.41	2.00E-03
11737527_s_at	DYRK1A	1.46	2.00E-03
11731512_a_at	DYRK4	1.48	5.00E-04
11722703_a_at	DZIP1	0.64	3.00E-04
11730029_a_at	E2F7	2.50	3.00E-04
11715346_at	EBI3	0.45	2.00E-04
11756448_x_at	EBNA1BP2	0.59	2.00E-06
11717859_a_at	EBP	1.60	1.00E-04
11757606_s_at	ECHS1	0.75	4.00E-03
11724159_x_at	ECSCR	0.76	8.00E-03
11728640_s_at	EDARADD /// ENO1	0.70	3.00E-03
11722260_a_at	EDEM1	1.72	8.00E-03
11747223_a_at	EDNRB	3.18	1.00E-03
11724302_a_at	EEA1	0.49	3.00E-03
11748659_a_at	EFEMP1	0.71	6.00E-05
11733857_x_at	EGLN1	0.68	6.00E-04
11756805_a_at	EHMT1	0.69	9.00E-04
11754143_x_at	EIF1	1.25	9.00E-03
11754107_a_at	EIF1B	1.62	2.00E-03
11750883_a_at	EIF2A	1.46	3.00E-04
11717673_a_at	EIF2B2	1.57	4.00E-03
11717831_a_at	EIF4E2	0.79	4.00E-03
11718284_x_at	EIF4EBP1	1.61	9.00E-04
11716530_at	ELAVL1	0.70	4.00E-05
11721574_a_at	ELF4	1.61	9.00E-03
11747799_x_at	ENO1	0.74	6.00E-03
11758802_a_at	ENY2	0.58	3.00E-03
11757980_s_at	EP400	0.73	4.00E-04
11760477_a_at	EP400NL	0.58	6.00E-03
11754990_a_at	EPB41	1.74	4.00E-03
11718952_at	EPB41L5	0.74	1.00E-02
11751886_s_at	ERBB2IP	1.62	8.00E-04
11717915_s_at	ERCC5	1.55	1.00E-03
11740067_a_at	ERCC6 /// PGBD3	0.59	6.00E-03
11716643_s_at	ERLEC1	1.73	4.00E-03
11730914_a_at	ERLIN1	0.71	5.00E-03
11734856_at	ERO1LB	3.16	2.00E-04
11718355_x_at	ERP44	1.57	8.00E-04
11718354_a_at	ERP44	1.51	6.00E-03
11751065_x_at	EXOG	0.68	5.00E-03

11736034_x_at	EXOSC2	0.55	1.00E-03
11731563_a_at	EXOSC7	0.76	3.00E-03
11753011_a_at	EXOSC9	0.66	1.00E-03
11750985_a_at	EXTL2	0.57	9.00E-03
11717657_a_at	EZH2	0.62	8.00E-03
11755733_a_at	F3	1.88	3.00E-03
11752670_a_at	FADS1	2.11	6.00E-06
11744900_x_at	FADS2	1.69	6.00E-05
11741413_a_at	FAM101A	0.26	2.00E-03
11754371_a_at	FAM102A	2.30	2.00E-04
11755895_a_at	FAM129A	3.20	8.00E-04
11758621_s_at	FAM134A	1.39	7.00E-03
11717908_s_at	FAM136A	0.62	2.00E-04
11757671_a_at	FAM200B	0.61	7.00E-04
11744550_x_at	FAM36A	0.71	1.00E-02
11722342_a_at	FAM38B	0.49	3.00E-04
11743609_a_at	FAM3C	1.46	7.00E-03
11716746_a_at	FAM60A	0.73	2.00E-03
11723747_a_at	FAM64A	0.47	2.00E-03
11735324_s_at	FAM72A /// FAM72B /// FAM72C /// FAM72D	0.61	5.00E-03
11730875_a_at	FANCI	0.64	9.00E-03
11747638_a_at	FARSB	0.72	5.00E-03
11756878_a_at	FBL	0.72	3.00E-03
11720507_at	FBN1	0.66	5.00E-03
11737032_x_at	FBXL13	3.41	1.00E-03
11757243_s_at	FBXL20	1.53	8.00E-03
11743854_s_at	FBXO21	0.74	9.00E-03
11723321_at	FBXO30	1.57	5.00E-03
11719394_a_at	FBXO32	2.45	6.00E-05
11724529_s_at	FBXO8	1.45	3.00E-04
11721242_s_at	FDFT1	2.14	2.00E-04
11756481_x_at	FDXR	0.71	6.00E-03
11726393_at	FGD4	0.52	8.00E-05
11725155_at	FJX1	0.37	2.00E-05
11733701_a_at	FKBP11	1.74	1.00E-04
11759118_at	FKBP14	1.53	7.00E-03
11720633_a_at	FLNC	0.70	7.00E-04
11744631_a_at	FLT1	1.92	3.00E-03
11731054_s_at	FNIP1	1.80	5.00E-03
11723091_s_at	FNIP2	1.79	1.00E-04
11757728_s_at	FTL	1.38	5.00E-03
11728310_a_at	FTSJD1	0.64	3.00E-03
11745190_a_at	FUS	0.71	2.00E-03
11725291_at	FUT1	0.69	8.00E-03
11757655_a_at	FXC1	1.39	1.00E-03
11727552_at	FZD2	2.11	6.00E-04
11758023_x_at	GABARAPL1	2.06	1.00E-03
11723069_at	GABBR1 /// UBD	0.14	3.00E-09
11737879_s_at	GALNT2	0.73	3.00E-03
11758592_s_at	GAR1	0.70	6.00E-03
11753958_a_at	GARS	2.11	2.00E-05
11723838_at	GATA6	0.44	1.00E-02
11720049_a_at	GBE1	1.54	3.00E-05
11726329_x_at	GBP1	0.49	5.00E-04
11733951_at	GBP4	0.38	3.00E-04
11747086_a_at	GCA	0.66	7.00E-03
11754931_a_at	GCLC	1.41	4.00E-03
11723489_at	GCLM	2.22	1.00E-04
11735271_a_at	GCNT1	0.58	7.00E-03
11733746_x_at	GCNT2	2.38	3.00E-05
11741301_s_at	GCOM1 /// GRINL1A	1.54	2.00E-03
11716664_x_at	GDF15	2.87	1.00E-04

11734363_at	GDF6	0.32	1.00E-05
11733935_a_at	GEMIN4	0.59	2.00E-03
11742740_at	GEMIN5	0.59	2.00E-05
11720221_at	GFPT1	2.03	3.00E-03
11729246_x_at	GGCX	0.68	6.00E-03
11720346_at	GGNBP2	1.45	7.00E-03
11728863_at	GINS1	0.41	6.00E-04
11743513_a_at	GINS2	0.31	3.00E-09
11736596_s_at	GINS4	0.58	3.00E-03
11716718_at	GLA	2.16	9.00E-05
11720063_a_at	GLIPR2	0.48	4.00E-04
11747441_a_at	GLRX	2.25	2.00E-04
11739063_at	GNG12	0.53	5.00E-03
11743701_at	GNL3L	0.68	1.00E-03
11754010_x_at	GOLGA2	1.52	6.00E-04
11744729_x_at	GOLGA4	1.84	2.00E-03
11719387_a_at	GOLGA5	1.46	9.00E-03
11754456_a_at	GOLGB1	1.72	4.00E-03
11754179_a_at	GOLT1B	2.07	1.00E-05
11757538_a_at	GOT1	2.77	4.00E-03
11721649_a_at	GPATCH4	0.60	3.00E-03
11755533_a_at	GPR125	0.59	8.00E-03
11759208_at	GPR176	1.61	9.00E-03
11719162_a_at	GPT2	1.82	1.00E-03
11757266_s_at	GPX3	2.11	1.00E-02
11729644_a_at	GPX8	1.91	8.00E-03
11722785_x_at	GRWD1	0.70	2.00E-03
11759080_at	GSR	1.54	2.00E-03
11749979_s_at	GTF2H2 /// GTF2H2B /// GTF2H2C /// GTF2H2D	0.68	1.00E-03
11750604_a_at	GTF2IRD1	1.91	7.00E-05
11759176_at	GTSE1	0.59	9.00E-03
11724519_a_at	GUF1	0.64	4.00E-03
11754243_a_at	H2AFX	0.64	2.00E-03
11735189_s_at	H2AFY	0.78	5.00E-03
11722555_s_at	HADH	0.55	8.00E-05
11741031_a_at	HAX1	1.56	2.00E-04
11716698_s_at	HBP1	1.40	8.00E-04
11717036_a_at	HDAC5	2.22	3.00E-04
11736838_a_at	HDAC9	1.92	4.00E-03
11755821_x_at	HDLBP	1.36	3.00E-03
11755789_a_at	HEATR1	0.77	9.00E-03
11758931_a_at	HEATR5A	1.78	4.00E-03
11755566_a_at	HEATR7A	1.45	2.00E-03
11749691_a_at	HELLS	0.40	7.00E-08
11749257_a_at	HERPUD1	3.61	3.00E-03
11718201_at	HEY1	6.54	5.00E-05
11742779_a_at	HIBCH	0.71	8.00E-03
11740470_x_at	HM13	1.67	1.00E-03
11727375_a_at	HMGCR	4.38	1.00E-03
11716987_a_at	HMGCS1	4.14	1.00E-11
11724740_x_at	HMGN2	0.60	5.00E-05
11730856_s_at	HMGN3	0.69	9.00E-03
11753446_x_at	HMOX1	10.56	3.00E-07
11717727_s_at	HN1	0.59	6.00E-05
11728660_at	HN1L	0.64	5.00E-03
11716291_s_at	HNRNPA0	0.68	1.00E-04
11742679_s_at	HNRNPA2B1	0.68	1.00E-02
11723197_at	HNRNPA3	0.68	2.00E-03
11723199_s_at	HNRNPA3 /// HNRNPA3P1	0.75	5.00E-03
11718220_x_at	HNRNPAB	0.73	2.00E-03
11758144_x_at	HNRNPC	0.85	8.00E-03
11749303_s_at	HNRNPD	0.66	5.00E-03

11716659_a_at	HNRNPH3	0.66	2.00E-03
200072_PM_s_at	HNRNPM	0.71	9.00E-04
11725617_s_at	HNRNPR	0.73	3.00E-03
11722001_a_at	HNRNPU	0.75	4.00E-03
11718270_s_at	HNRNPUL1	0.74	7.00E-03
11758015_s_at	HPRT1	0.72	3.00E-03
11755915_a_at	HPS3	1.54	5.00E-03
11717515_a_at	HSD17B10	0.76	1.00E-02
11730778_a_at	HSD17B11	1.97	4.00E-04
11737762_x_at	HSD17B4	1.30	9.00E-03
11732374_x_at	HSD17B7	2.30	2.00E-04
11753260_x_at	HSP90B1	1.79	2.00E-03
11761479_x_at	HSP90B2P	1.62	4.00E-03
11719071_s_at	HSPA13	2.39	9.00E-06
11758770_at	HSPA4	0.68	5.00E-03
11728697_a_at	HSPA4L	0.59	9.00E-04
11725931_at	HSPA5	3.61	1.00E-03
11759417_s_at	HSPA9	1.96	4.00E-03
11715775_a_at	HSPB8	3.20	1.00E-05
11740073_a_at	HTATIP2	1.51	9.00E-03
11744584_a_at	HYI	0.48	2.00E-04
11748625_x_at	HYOU1	1.89	4.00E-03
11751966_a_at	IARS	1.67	6.00E-03
11753919_a_at	IDE	1.43	9.00E-03
11718474_a_at	IDH1	2.00	1.00E-03
11739383_a_at	IDI1	3.56	8.00E-07
11732132_a_at	IDS	1.47	8.00E-03
11716796_s_at	IFI16	1.60	5.00E-04
11754061_x_at	IFITM2	0.64	4.00E-03
11729406_a_at	IFNGR1	1.44	7.00E-03
11748629_a_at	IFRD1	2.16	7.00E-04
11755876_a_at	IFRD2	0.67	7.00E-04
11716129_at	IGF2R	1.25	5.00E-03
11717909_at	IGFBP6	0.31	3.00E-07
11732110_a_at	IL4I1	1.77	2.00E-04
11746463_a_at	IL6	3.89	9.00E-03
11740781_a_at	ILF3	0.55	3.00E-03
11757812_s_at	INPP5A	1.48	8.00E-03
11716339_a_at	INSIG1	3.92	2.00E-08
11742676_a_at	IRF2BP2	2.20	3.00E-06
11739415_at	IRGQ	0.75	5.00E-03
11743617_at	ITGA2	1.67	1.00E-03
11735066_at	ITGB3	0.45	2.00E-04
11739538_at	ITPR2	0.61	7.00E-03
11728073_a_at	ITPRIP	1.56	6.00E-03
11722552_x_at	ITSN1	0.49	6.00E-06
11730903_a_at	JDP2	2.04	3.00E-05
11732640_a_at	JKAMP	1.36	4.00E-03
11720283_at	JMY	1.85	1.00E-03
11755160_a_at	JOSD1	1.64	2.00E-06
11758652_s_at	KANK1	1.80	2.00E-03
11736458_x_at	KCNK6	1.60	6.00E-04
11715951_s_at	KCTD12	0.43	2.00E-04
11756019_a_at	KDELR2	1.45	7.00E-04
11730045_a_at	KDELR3	1.53	3.00E-03
11716574_at	KDM3A	1.77	2.00E-05
11722928_a_at	KIAA0415	1.73	1.00E-04
11718635_at	KIF1B	1.52	3.00E-03
11723010_a_at	KIF20A	0.40	2.00E-03
11721932_a_at	KIF23	0.56	8.00E-03
11728955_a_at	KITLG	1.99	9.00E-03
11727145_s_at	KLF11	2.23	1.00E-04

11721805_at	KLF2	1.79	3.00E-03
11719633_a_at	KLF4	1.46	7.00E-04
11756499_a_at	KLHDC2	1.37	7.00E-03
11744409_a_at	KLHL18	0.67	4.00E-03
11718984_a_at	KLHL21	2.25	2.00E-05
11760229_x_at	KLHL23	0.55	3.00E-03
11756440_a_at	KLHL24	2.64	1.00E-05
11762368_at	KLHL8	0.73	6.00E-03
11754765_s_at	KPNA2 /// LOC146880 /// PLEKHM1P	0.72	8.00E-04
11728288_a_at	KRT15	0.66	6.00E-03
11754941_x_at	KRT18	0.46	2.00E-06
11715683_a_at	KRT7	0.32	1.00E-05
11715629_a_at	LAPTM4A	1.26	9.00E-03
11755709_s_at	LARP6	3.48	2.00E-04
11721074_a_at	LAS1L	0.73	1.00E-03
11754581_s_at	LASS6	0.70	7.00E-03
11755581_a_at	LATS2	0.69	6.00E-03
11716162_at	LBH	3.41	1.00E-04
11733592_x_at	LCA5	1.56	4.00E-04
11742116_x_at	LDHA	0.59	1.00E-04
11720028_x_at	LDLR	4.00	1.00E-08
11734157_a_at	LENG1	1.31	1.00E-03
11748131_a_at	LGALS8	2.06	1.00E-03
11734594_a_at	LIN7B	1.80	4.00E-03
11724213_at	LMAN1	2.16	9.00E-03
11750442_s_at	LMNB1 /// PCIF1	0.52	8.00E-03
11716170_at	LMNB2	0.66	5.00E-03
11721660_s_at	LMO4	2.10	3.00E-06
11737378_s_at	LOC100132288 /// MAFIP /// TEKT4P1	1.33	6.00E-03
11763303_at	LOC100506748	1.52	2.00E-03
11731632_s_at	LOC392288 /// MAP1LC3B	2.28	5.00E-06
11718376_s_at	LOC401127 /// WDR5	0.70	1.00E-03
11753773_x_at	LOC653566 /// SPCS2	1.67	6.00E-03
11756139_s_at	LOC728554 /// THOC3	0.66	3.00E-04
11725963_a_at	LONP1	1.51	3.00E-03
11718832_a_at	LOXL2	0.72	9.00E-04
11715638_s_at	LPCAT1	1.96	2.00E-05
11755123_x_at	LPCAT3	0.52	3.00E-03
11757747_s_at	LPIN1	1.57	1.00E-05
11728896_a_at	LRP8	2.06	1.00E-04
11723248_at	LRRC40	0.70	4.00E-04
11748065_a_at	LRRC49	3.16	7.00E-06
11757324_s_at	LSM12	0.77	3.00E-03
11723958_s_at	LSM5	0.66	5.00E-03
11728490_a_at	LSMD1	0.74	4.00E-03
11717620_a_at	LSS	1.61	4.00E-03
11739658_a_at	LTB	0.64	2.00E-03
11719873_x_at	LTV1	0.73	9.00E-03
11728507_at	LY96	1.85	3.00E-03
11722839_at	LYAR	0.65	2.00E-03
11741122_a_at	LYPD1	0.36	4.00E-09
11731734_a_at	LYPD6	0.44	6.00E-06
11721149_a_at	LYSMD2	0.74	6.00E-03
11758398_s_at	LYSMD3	1.99	3.00E-03
11722982_a_at	LYST	1.79	5.00E-03
11743773_s_at	LZTFL1	2.14	5.00E-04
11748136_a_at	MAD2L1	0.50	2.00E-03
11717133_a_at	MAFG	1.61	6.00E-05
11719388_x_at	MAFK	1.64	3.00E-03
11715222_at	MAGI3	0.67	1.00E-03
11755486_s_at	MAGT1	1.87	6.00E-09
11725393_s_at	MAK16	0.66	2.00E-04

11723447_at	MALL	3.41	3.00E-03
11736101_at	MAN1A1	1.83	1.00E-03
11715795_at	MANF	2.51	9.00E-05
11727029_a_at	MAP1A	1.77	6.00E-04
11748422_a_at	MAP1LC3B	2.14	3.00E-05
11736559_a_at	MAP2	3.29	2.00E-07
11742948_at	MARCKS	0.55	2.00E-03
11755845_x_at	MARS	1.54	7.00E-03
11729711_at	MARS2	0.37	3.00E-06
11756985_a_at	MAT2A	0.64	9.00E-03
11719799_a_at	MBD4	1.39	9.00E-03
11729236_at	MCCC2	0.70	9.00E-03
11715487_a_at	MCL1	0.65	4.00E-03
11736367_a_at	MCM10	0.49	4.00E-04
11756666_x_at	MCM2	0.62	3.00E-03
11742997_x_at	MCM3	0.62	7.00E-05
11736670_a_at	MCM4	0.45	2.00E-06
11732997_a_at	MCM6	0.39	6.00E-06
11755469_x_at	MCM7	0.63	5.00E-03
11763965_s_at	MDN1	0.54	9.00E-08
11739452_a_at	ME2	0.71	3.00E-03
11750038_a_at	MECR	0.71	6.00E-03
11724815_at	MED13L	1.51	4.00E-03
11734794_a_at	MED31	1.46	7.00E-04
11724863_a_at	MEF2A	1.78	4.00E-03
11755577_x_at	MEI1	2.87	1.00E-03
11724650_a_at	MEIS2	0.57	1.00E-03
11717196_a_at	MEST	0.11	2.00E-03
11722330_at	MEX3A	0.49	1.00E-03
11724499_at	MFNG	0.64	6.00E-03
11752336_x_at	MFSD11	1.49	1.00E-03
11750984_a_at	MGAT2	1.41	5.00E-03
11752439_s_at	MIA3	1.52	2.00E-04
11718480_s_at	MID1IP1	1.45	9.00E-03
11739591_s_at	MINA	0.66	5.00E-03
11752442_a_at	MIR17HG	0.62	5.00E-03
11763310_at	MIR21	1.66	9.00E-03
11718657_a_at	MIR21 /// TMEM49	1.68	3.00E-05
11721144_a_at	MKI67	0.53	4.00E-03
11753830_a_at	MLLT11	2.95	1.00E-03
11736866_a_at	MMP16	0.54	2.00E-03
11764146_s_at	MOSPD2	1.51	7.00E-03
11743466_at	MPHOSPH6	0.56	1.00E-05
11744376_a_at	MPI	0.71	1.00E-03
11719834_a_at	MPZL2	0.40	4.00E-03
11763627_a_at	MRPL12	0.71	2.00E-03
11715995_at	MRPL16	0.77	7.00E-03
11727410_a_at	MRPL19	0.76	5.00E-03
11717643_a_at	MRPL24	0.66	1.00E-04
11757551_a_at	MRPL3	0.70	4.00E-04
11756687_a_at	MRPL46	0.75	8.00E-03
11730213_s_at	MRPS7	0.76	7.00E-03
11724751_at	MRTO4	0.63	4.00E-03
11729288_at	MSL3	1.44	6.00E-03
11757718_a_at	MTDH	1.55	1.00E-03
11716667_a_at	MTHFD1	0.53	2.00E-05
11745892_a_at	MTHFD2	3.61	3.00E-05
11753047_a_at	MTHFD2L	1.93	6.00E-03
11727178_x_at	MTHFR	1.73	5.00E-03
11733512_s_at	MTMR1	0.70	4.00E-03
11739413_at	MTR	0.68	7.00E-03
11729695_a_at	MVD	1.60	9.00E-05

11719098_a_at	MVK	1.34	5.00E-03
11716167_a_at	MX1	0.33	3.00E-03
11719249_at	MXD1	1.83	3.00E-03
11725861_a_at	MYBL1	0.43	1.00E-04
11745021_a_at	MYC	0.57	3.00E-05
11727802_s_at	MYCBP	0.70	4.00E-03
11730878_x_at	MYCT1	1.66	1.00E-04
11754984_s_at	MYH10	0.49	5.00E-03
11718006_a_at	MYLIP	0.68	9.00E-03
11721837_a_at	MYLK	4.96	4.00E-06
11740233_s_at	MYST4	1.59	6.00E-05
11743709_at	NAA15	0.67	6.00E-03
11717250_a_at	NAE1	0.68	1.00E-03
11748516_a_at	NAP1L5	0.58	4.00E-05
11722631_s_at	NARG2	0.75	3.00E-03
11743505_a_at	NARS2	0.66	7.00E-03
11723147_a_at	NAV3	1.73	9.00E-05
11720550_a_at	NCAPD3	0.67	7.00E-03
11756784_x_at	NCAPG2	0.63	5.00E-03
11725978_at	NCBP1	0.78	6.00E-03
11718026_a_at	NCOA7	0.47	2.00E-03
11759550_at	NCRNA00275	0.59	3.00E-03
11753431_x_at	NDRG1	2.46	3.00E-04
11741547_a_at	NEDD4L	2.43	1.00E-03
11723394_s_at	NEIL2	0.62	4.00E-05
11724821_a_at	NEK6	0.58	3.00E-05
11717451_at	NEK7	0.47	8.00E-03
11722865_at	NETO2	0.56	4.00E-04
11715838_a_at	NEU1	1.74	2.00E-05
11719746_a_at	NF2	0.50	2.00E-03
11743380_s_at	NFE2L2	1.49	1.00E-03
11722661_at	NFE2L3	0.57	9.00E-03
11717219_s_at	NFIB	0.65	7.00E-03
11743010_at	NFIL3	3.97	2.00E-04
11727161_a_at	NFIX	0.52	9.00E-03
11757726_x_at	NHP2	0.70	2.00E-03
11727849_a_at	NIP7	0.70	9.00E-04
11725627_at	NIPAL3	1.55	3.00E-04
11732404_a_at	NKRF	0.74	4.00E-03
11725117_a_at	NLN	0.63	1.00E-04
11717155_a_at	NME1 /// NME1-NME2	0.60	1.00E-05
11757561_s_at	NOC2L	0.73	9.00E-03
11722997_s_at	NOL11	0.71	3.00E-03
11715697_a_at	NOLC1	0.64	2.00E-04
11715301_s_at	NOP16	0.51	5.00E-06
11736019_a_at	NOP2	0.50	1.00E-04
11758679_s_at	NOP56	0.55	1.00E-06
11743916_a_at	NPC1	3.84	4.00E-11
11717567_a_at	NQO1	1.52	4.00E-03
11722087_a_at	NR1D2	2.22	2.00E-04
11744801_a_at	NR2C2AP	0.65	6.00E-03
11732248_at	NR2F1	0.56	5.00E-03
11735194_a_at	NR3C1	1.67	1.00E-02
11727749_a_at	NR5A2	0.37	6.00E-04
11741386_a_at	NRF1	0.70	1.00E-03
11740346_a_at	NRG1	0.42	5.00E-05
11728221_at	NRIP3	2.19	4.00E-03
11720522_a_at	NSDHL	2.00	2.00E-06
11716978_a_at	NSFL1C	1.42	1.00E-04
11763187_a_at	NUCB2	2.35	9.00E-05
11732160_a_at	NUDCD1	0.46	9.00E-04
11730839_at	NUDT16	0.68	5.00E-03

11758034_s_at	NUDT16L1	0.70	4.00E-03
11754453_s_at	NUDT4	0.52	4.00E-04
11758559_s_at	NUDT4 /// NUDT4P1	0.55	3.00E-03
11717946_x_at	NUMB	1.29	8.00E-03
11736253_a_at	NUP160	0.67	3.00E-03
11750468_a_at	NUP35	0.61	1.00E-03
11727856_s_at	NUP50	0.71	7.00E-04
11754814_a_at	NUP93	0.71	1.00E-04
11716937_a_at	NUPR1	4.72	2.00E-06
11723698_a_at	OAS3	0.56	6.00E-04
11727543_at	OIP5	0.45	3.00E-04
11744228_x_at	ORAI3	1.66	2.00E-04
11755384_s_at	OSBP	1.74	5.00E-03
11744045_a_at	OSGIN1	1.78	1.00E-03
11743412_at	OTUD1	2.89	6.00E-06
11748548_a_at	P2RX4	2.08	4.00E-03
11730853_s_at	P4HA1	1.40	5.00E-03
11723412_s_at	PA2G4	0.61	6.00E-04
11718303_a_at	PAFAH1B3	0.63	3.00E-04
11728043_at	PANX2	2.57	2.00E-05
11726249_a_at	PARP1	0.51	1.00E-02
11757624_s_at	PARP12	0.55	8.00E-04
11744434_a_at	PARP9	0.52	4.00E-03
11755176_a_at	PAXIP1	0.61	6.00E-03
11723950_a_at	PBK	0.43	1.00E-03
11749105_a_at	PCDH1	2.38	5.00E-07
11745210_s_at	PCDH9	2.04	6.00E-03
11739196_a_at	PCGF3	0.58	1.00E-03
11728867_a_at	PCK2	2.10	1.00E-05
11716103_a_at	PCNA	0.55	4.00E-04
11734920_s_at	PCYOX1L	0.68	1.00E-02
11717304_x_at	PDAP1	0.77	7.00E-03
11723985_a_at	PDCD11	0.74	2.00E-03
11722085_a_at	PDCD2L	0.64	3.00E-04
11723484_x_at	PDCL3	0.71	2.00E-03
11754738_a_at	PDE2A	0.54	8.00E-03
11720579_a_at	PDE5A	0.26	3.00E-06
11723338_a_at	PDGFC	0.43	2.00E-04
11743440_at	PDIA3	1.38	7.00E-05
11716118_a_at	PDIA4	1.85	2.00E-04
11716919_a_at	PDIA5	1.36	6.00E-04
11745720_s_at	PDIA6	1.39	3.00E-04
11743904_s_at	PDLIM1	0.49	9.00E-05
11717107_a_at	PDLIM5	0.67	4.00E-03
11720547_a_at	PDSS1	0.45	7.00E-08
11725762_a_at	PELI3	1.59	2.00E-05
11719918_s_at	PELO	1.87	3.00E-04
11719285_a_at	PEX11B	0.67	2.00E-03
11726711_x_at	PFAS	0.46	4.00E-06
11760362_x_at	PGAM5	0.57	3.00E-03
11755641_s_at	PGAP1	0.60	1.00E-04
11726504_at	PGBD3	0.32	6.00E-06
11763174_at	PGM3	1.55	7.00E-03
11720609_at	PHB	0.63	1.00E-04
11722880_a_at	PHF19	0.64	1.00E-03
11742830_a_at	PHF3	1.41	5.00E-04
11728203_a_at	PHGDH	4.38	5.00E-08
11732188_at	PI4K2A	1.56	1.00E-04
11729150_at	PIGM	0.70	1.00E-02
11729215_x_at	PIGX	0.64	3.00E-03
11724361_s_at	PIKFYVE	1.38	6.00E-03
11717257_s_at	PIM1	1.57	1.00E-03

11715888_s_at	PIP4K2B	0.69	3.00E-03
11756548_s_at	PKP4	0.67	6.00E-03
11743056_at	PLAA	1.64	3.00E-05
11743062_a_at	PLAUR	1.60	1.00E-03
11731118_x_at	PLEKHA9	1.74	1.00E-04
11755184_x_at	PLEKHM3	1.69	2.00E-04
11722403_a_at	PLEKHO1	1.45	1.00E-03
11742745_a_at	PLIN2	3.32	4.00E-06
11756417_x_at	PLK1S1	1.54	7.00E-03
11722052_at	PLLP	0.48	4.00E-03
11721333_a_at	PLOD2	0.61	3.00E-03
11751652_x_at	PLSCR1	0.60	4.00E-03
11741315_a_at	PLSCR4	0.29	2.00E-05
11727712_at	PLXNA4	0.37	1.00E-05
11733425_at	PMCH	2.87	1.00E-03
11748770_a_at	PMPCA	0.74	4.00E-03
11756161_x_at	PMVK	0.65	1.00E-02
11757503_s_at	PNN	0.56	1.00E-05
11729187_x_at	PNO1	0.65	1.00E-04
11718400_a_at	PNP	1.68	2.00E-07
11739766_at	PNPLA3	2.36	2.00E-03
11745750_a_at	PNPLA8	2.43	1.00E-04
11722377_at	PNPO	0.63	6.00E-03
11734726_x_at	PNPT1	0.59	5.00E-03
11715763_a_at	PODXL	1.82	7.00E-05
11726156_at	POLA1	0.60	7.00E-04
11744519_a_at	POLE2	0.42	4.00E-07
11755956_x_at	POLE3	0.68	7.00E-04
11722215_at	POLE4	0.67	4.00E-03
11724544_a_at	POLR1A	0.69	3.00E-03
11741721_x_at	POLR1B	0.54	3.00E-04
11720324_a_at	POLR2F	0.76	2.00E-03
11756185_s_at	POLR3K	0.59	1.00E-03
11723138_at	POP1	0.56	1.00E-03
11721868_a_at	POP5	0.56	4.00E-04
11720213_x_at	PPAN	0.71	3.00E-03
11715739_s_at	PPAP2B	3.58	9.00E-05
11730681_at	PPAPDC1B	1.56	3.00E-03
11731899_s_at	PPAT	0.64	7.00E-04
11725712_s_at	PPIA	0.69	9.00E-03
11715607_at	PPIB	1.29	9.00E-03
11719382_a_at	PPIH	0.69	9.00E-03
11744465_x_at	PPP1R15A	1.74	7.00E-05
11722420_a_at	PPP3CC	1.58	2.00E-03
11718607_x_at	PPPDE2	1.48	2.00E-04
11748712_a_at	PPRC1	0.58	3.00E-04
11716533_a_at	PPT1	0.72	2.00E-03
11755451_x_at	PQLC2	1.54	5.00E-04
11716358_s_at	PRC1	0.55	2.00E-03
11728171_a_at	PRDM10	0.70	8.00E-03
11727804_s_at	PRDX3	0.78	4.00E-03
11753861_x_at	PRICKLE4 /// TOMM6	0.80	6.00E-03
11730261_a_at	PRKAR2B	0.58	7.00E-04
11736257_at	PRKCE	1.62	1.00E-03
11735730_s_at	PRKRIR	0.68	2.00E-05
11742086_a_at	PRMT3	0.66	2.00E-03
11751039_a_at	PRMT6	0.55	3.00E-03
11736151_a_at	PRMT7	0.63	2.00E-04
11715679_s_at	PRNP	2.28	3.00E-06
11759011_at	PRPF4	0.58	4.00E-04
11744848_x_at	PRR16	1.61	5.00E-03
11747349_s_at	PSAT1	5.98	6.00E-09

11715668_a_at	PSMA5	1.37	3.00E-03
200039_PM_s_at	PSMB2	1.37	3.00E-03
11715407_s_at	PSMB4	1.34	4.00E-03
11715596_at	PSMB7	1.45	7.00E-05
11715901_a_at	PSMC2	1.48	1.00E-03
11734865_a_at	PSMC3IP	0.62	7.00E-04
11718881_a_at	PSMC4	1.61	2.00E-04
11744362_a_at	PSMD14	1.67	1.00E-04
11715515_a_at	PSMD3	1.64	1.00E-05
11729114_s_at	PSMD7	1.38	1.00E-03
11756207_s_at	PSME3	0.75	7.00E-03
11743499_a_at	PSMG4	0.61	3.00E-03
11730796_x_at	PSPH	1.83	2.00E-03
11729338_x_at	PSTPIP2	0.40	1.00E-05
11742457_s_at	PTBP1	0.74	2.00E-03
11758421_s_at	PTEN	1.37	3.00E-03
11725793_s_at	PTGER4	0.33	6.00E-03
11717340_at	PTGFRN	0.53	2.00E-04
11724441_x_at	PTGIS	0.31	1.00E-04
11736655_a_at	PTGR1	2.04	6.00E-04
11724038_a_at	PTGS2	4.89	1.00E-03
11757829_a_at	PTPLAD1	0.76	7.00E-03
11748647_a_at	PTPRR	2.10	3.00E-03
11730730_s_at	PTRF	0.70	4.00E-03
11743212_x_at	PTTG2	0.74	6.00E-03
11757414_x_at	PUF60	0.78	7.00E-03
11718065_a_at	PVRL2	0.66	4.00E-04
11757394_a_at	PWP2	0.62	2.00E-04
11723314_x_at	PXMP2	0.54	6.00E-05
11718210_s_at	PYCR1	1.53	5.00E-03
11723801_s_at	PYROXD1	1.69	4.00E-03
11722214_a_at	QTRT1	0.61	6.00E-03
11716019_at	RAB31	1.38	6.00E-03
11754764_a_at	RAB7B	0.52	7.00E-04
11716287_at	RABAC1	1.32	4.00E-03
11720808_a_at	RABEPK	0.74	8.00E-03
11757294_x_at	RABGGTB	1.43	4.00E-03
11732191_s_at	RAD51AP1	0.54	8.00E-03
11732520_a_at	RAD51C	0.68	5.00E-03
11717758_x_at	RALA	1.58	1.00E-03
11753560_s_at	RAN	0.70	5.00E-03
11723001_s_at	RANBP1	0.56	6.00E-06
11720369_at	RANBP6	0.68	1.00E-03
11717164_a_at	RANGAP1	0.63	1.00E-03
11758403_s_at	RAPH1	0.65	1.00E-03
11722116_a_at	RARB	0.29	9.00E-05
11716839_a_at	RASD1	3.32	1.00E-06
11743480_s_at	RB1CC1	1.62	1.00E-03
11743869_a_at	RBBP9	0.70	8.00E-03
11719924_a_at	RBM12	0.64	5.00E-04
11717422_s_at	RBM8A	0.72	1.00E-03
11728285_at	RBMS2	0.66	5.00E-03
11757795_s_at	RCOR3	1.43	3.00E-04
11719626_at	RDH10	2.11	9.00E-04
11743034_x_at	RDH11	1.59	4.00E-03
11759658_a_at	RECK	0.52	6.00E-04
11750034_a_at	REEP5	1.36	1.00E-03
11744199_a_at	RETSAT	1.36	8.00E-04
11718404_at	RFC3	0.51	1.00E-04
11741284_a_at	RFC5	0.53	4.00E-05
11716046_a_at	RGL2	1.54	1.00E-03
11741572_a_at	RGS4	0.32	5.00E-06

11754028_s_at	RHEB	1.46	5.00E-03
11731613_a_at	RHEBL1	1.60	2.00E-03
11759345_at	RHOJ	0.68	1.00E-03
11757340_s_at	RHOQ	2.53	3.00E-05
11725950_s_at	RIOK3	1.61	3.00E-04
11725079_a_at	RIT1	1.47	6.00E-03
11718988_s_at	RNASE4	2.89	2.00E-05
11757017_x_at	RNASEH2A	0.60	5.00E-04
11717807_at	RNF103	1.46	2.00E-04
11743013_a_at	RNF115	1.79	2.00E-05
11754399_a_at	RNF121	0.50	1.00E-04
11758506_s_at	RNF13	1.80	3.00E-04
11723232_x_at	RNF138	0.58	6.00E-05
11723231_s_at	RNF138 /// RNF138P1	0.53	8.00E-04
11716443_at	RNF181	1.30	9.00E-03
11716735_s_at	RNF185	1.35	3.00E-03
11716572_s_at	RPA1	0.67	3.00E-04
11759540_a_at	RPL35A	0.65	9.00E-05
11754767_x_at	RPL7L1	0.76	8.00E-03
11742852_a_at	RPN1	1.62	2.00E-05
11725715_x_at	RPS14	0.66	1.00E-03
11732942_a_at	RPS26	0.65	6.00E-03
11735610_a_at	RPS6KA5	2.10	4.00E-04
11722114_a_at	RPS6KC1	1.58	8.00E-03
11751353_a_at	RRAGC	1.48	2.00E-03
11750667_a_at	RRM1	0.62	2.00E-03
11758219_x_at	RRM2	0.31	2.00E-04
11721195_s_at	RRP1B	0.61	3.00E-05
11719810_at	RRS1	0.51	1.00E-08
11724619_at	RSPO3	0.25	4.00E-05
11747775_x_at	RUVBL1	0.67	2.00E-03
11730398_a_at	RUVBL2	0.73	4.00E-04
11725905_a_at	S1PR3	0.58	5.00E-03
11757604_a_at	SAMM50	0.78	6.00E-03
11744142_a_at	SARS	1.51	2.00E-03
11722241_x_at	SAV1	2.73	3.00E-04
11740092_x_at	SC4MOL	3.86	8.00E-07
11733780_a_at	SC5DL	2.41	5.00E-05
11756174_s_at	SCD	1.52	2.00E-03
11716433_s_at	SCD5	0.61	8.00E-04
11723960_at	SCFD2	0.65	1.00E-03
11742095_a_at	SCLY /// UBE2F	0.63	6.00E-03
11744785_x_at	SCRIB	0.64	1.00E-04
11718591_at	SDF2L1	2.99	4.00E-03
11725695_s_at	SDPR	0.34	8.00E-03
11744264_a_at	SEC11C	2.22	2.00E-08
11717319_a_at	SEC24D	1.60	3.00E-03
11755426_x_at	SEC61A1	1.57	2.00E-05
11716223_a_at	SEC61G	1.39	6.00E-04
11734049_a_at	SEH1L	0.65	4.00E-03
11717204_at	SEL1L	2.27	1.00E-04
11755826_a_at	SELP	0.55	9.00E-03
11742952_at	SELS	1.92	3.00E-04
11730391_at	SEMA3A	0.40	5.00E-04
11754308_s_at	SEPHS1	0.67	5.00E-03
11715806_a_at	SEPW1	0.75	5.00E-03
11758800_x_at	SERBP1	0.68	2.00E-04
11747464_a_at	SERINC3	1.51	4.00E-04
11720308_x_at	SERP1	1.64	6.00E-03
11727056_s_at	SERPIND1	0.16	3.00E-03
11741680_a_at	SERPINE2	2.91	2.00E-04
11718324_s_at	SESN2	3.25	2.00E-04

11715770_x_at	SET	0.78	1.00E-03
11746492_s_at	SGCE	1.56	9.00E-03
11721818_s_at	SH3BP5L	1.61	3.00E-04
11723370_s_at	SHANK3	1.56	3.00E-03
11754600_a_at	SHC3	0.46	2.00E-04
11746438_a_at	SHMT2	2.38	2.00E-05
11728887_x_at	SIP1	0.70	4.00E-03
11719728_s_at	SIRPA	1.69	2.00E-03
11729310_a_at	SKA3	0.61	8.00E-03
11751498_a_at	SLC10A7	1.61	8.00E-03
11744053_a_at	SLC11A2	1.61	3.00E-03
11730593_at	SLC16A12	0.35	6.00E-03
11758156_s_at	SLC17A5	1.78	2.00E-05
11721590_a_at	SLC17A9	1.74	7.00E-04
11721352_at	SLC1A1	2.97	6.00E-03
11730614_a_at	SLC1A4	3.34	2.00E-03
11716865_a_at	SLC1A5	1.59	1.00E-03
11756145_s_at	SLC25A15	0.63	1.00E-03
11724064_a_at	SLC25A19	0.61	1.00E-03
11717499_at	SLC25A36	1.33	6.00E-03
11744517_a_at	SLC2A10	1.64	2.00E-03
11734657_s_at	SLC2A14 /// SLC2A3	2.58	3.00E-05
11726006_at	SLC30A1	2.36	6.00E-03
11732336_at	SLC31A1	1.69	4.00E-03
11759757_a_at	SLC33A1	1.97	7.00E-04
11754813_a_at	SLC35B1	1.53	1.00E-03
11746569_x_at	SLC35C2	1.51	4.00E-04
11720075_s_at	SLC35F5	1.40	6.00E-03
11743090_a_at	SLC36A4	1.54	5.00E-03
11716064_a_at	SLC37A4	0.64	9.00E-03
11717102_a_at	SLC38A1	2.57	2.00E-05
11739000_a_at	SLC38A2	1.78	4.00E-04
11744715_a_at	SLC38A6	2.17	1.00E-06
11736570_a_at	SLC39A14	2.57	2.00E-03
11721095_x_at	SLC39A4 /// SLC39A7	1.42	6.00E-03
11733071_a_at	SLC3A2	3.29	5.00E-08
11720333_a_at	SLC5A6	0.62	9.00E-04
11721993_at	SLC6A6	2.01	3.00E-03
11722411_a_at	SLC7A1	1.53	9.00E-03
11721875_at	SLC7A11	2.27	2.00E-06
11743938_at	SLC7A14	0.41	1.00E-07
11739061_at	SLC7A5	2.51	3.00E-04
11733450_a_at	SLFN11	2.28	5.00E-03
11724469_at	SLU7	1.49	4.00E-04
11740886_a_at	SMARCA4	0.67	7.00E-03
11718773_s_at	SMARCE1	0.68	5.00E-03
11724351_a_at	SMC6	0.64	8.00E-04
11716139_s_at	SMS	0.76	7.00E-03
11723820_a_at	SMURF2	0.55	2.00E-04
11716700_at	SNAPIN	0.68	1.00E-03
11755133_x_at	SNHG7	1.58	2.00E-04
11755132_s_at	SNHG7 /// SNORA43	1.56	8.00E-03
11720630_a_at	SNRNP25	0.64	1.00E-03
11754463_a_at	SNRNP70	0.68	4.00E-03
11758650_s_at	SNRPC	0.76	7.00E-03
11724202_s_at	SNRPD1	0.66	9.00E-03
11763977_x_at	SNRPF	0.71	2.00E-03
11750607_a_at	SNX13	1.42	6.00E-03
11742742_a_at	SNX2	1.29	4.00E-03
11743589_at	SNX29	1.83	4.00E-03
11737753_s_at	SNX5	0.70	9.00E-03
11746543_a_at	SNX9	1.47	9.00E-03

11719430_at	SOS2	1.46	4.00E-03
11718897_at	SPAG9	2.08	7.00E-06
11759151_at	SPC24	0.47	3.00E-03
11723998_at	SPC25	0.30	6.00E-05
11721117_at	SPCS3	1.69	5.00E-04
11720101_s_at	SPIN1	0.77	7.00E-03
11720131_a_at	SPIRE1	1.54	7.00E-03
11717731_at	SQLE	2.08	2.00E-05
11745754_x_at	SQSTM1	2.10	1.00E-04
11752012_a_at	SREBF2	1.42	6.00E-03
11758254_s_at	SRI	0.61	3.00E-03
11717377_s_at	SRPRB	1.54	9.00E-04
11749302_s_at	SRSF2	0.72	3.00E-04
11751603_x_at	SRSF7	0.62	2.00E-06
200044_PM_at	SRSF9	0.82	3.00E-03
11716950_s_at	SRXN1	3.01	8.00E-09
11717054_a_at	SSR1	1.71	5.00E-05
11734069_s_at	SSR3	2.10	1.00E-03
11715749_s_at	SSRP1	0.75	1.00E-03
11743839_a_at	ST3GAL6	2.68	7.00E-05
11748321_x_at	STARD4	2.55	5.00E-03
11726372_a_at	STAU2	0.71	5.00E-03
11723400_a_at	STK4	0.65	6.00E-03
11753705_x_at	STMN1	0.51	3.00E-03
11754609_x_at	STMN3	0.70	8.00E-03
11725944_a_at	STT3A	1.43	4.00E-03
11729195_x_at	STX16	1.30	8.00E-03
11747878_a_at	STX3	2.22	8.00E-04
11751297_s_at	SUB1	0.63	9.00E-03
11717114_a_at	SUPT16H	0.78	6.00E-03
11751269_a_at	SUPT3H	1.64	6.00E-03
11730376_at	SURF2	0.70	7.00E-03
11715831_s_at	SURF4	1.48	1.00E-03
11720666_at	SYNCRIP	0.75	2.00E-03
11755757_a_at	SYNM	2.77	1.00E-05
11716172_at	SYVN1	2.57	4.00E-05
11720518_a_at	TACC2	1.74	2.00E-03
11715447_at	TALDO1	1.46	4.00E-04
11735341_s_at	TANC1	0.70	6.00E-03
11745266_x_at	TAP2	0.62	7.00E-03
11723832_a_at	TAPT1	1.44	7.00E-03
11727377_a_at	TBC1D15	2.16	1.00E-05
11728738_x_at	TBC1D20	1.52	1.00E-03
11731503_at	TBC1D4	1.89	3.00E-03
11728034_a_at	TBC1D7	1.96	2.00E-04
11723525_at	TBC1D8	2.10	6.00E-06
11717624_at	TBL2	1.53	4.00E-03
11735273_at	TBX18	0.58	3.00E-03
11721661_a_at	TBXAS1	2.48	1.00E-04
11755180_x_at	TCF7	0.55	2.00E-03
11725065_a_at	TCOF1	0.69	7.00E-03
11744699_a_at	TDP1	0.70	2.00E-03
11720230_at	TES	1.66	5.00E-03
11743642_s_at	TFAP2A	0.54	1.00E-03
11725349_s_at	TFDP1	0.70	1.00E-05
11748859_a_at	TFEC	3.46	6.00E-04
11715598_at	TFG	1.39	1.00E-03
11740596_a_at	TGFB2	0.32	8.00E-05
11718929_a_at	TGIF2	0.62	8.00E-03
11739781_a_at	TGM2	0.31	7.00E-06
11716901_s_at	TGOLN2	0.63	6.00E-03
11754439_x_at	TH1L	0.66	9.00E-04

11723682_at	THOC4	0.75	6.00E-03
11744117_at	THSD7A	0.43	7.00E-05
11730553_a_at	TICAM2 /// TMED7-TICAM2	2.68	2.00E-03
11719123_a_at	TIMELESS	0.55	5.00E-05
11727081_a_at	TIPIN	0.64	5.00E-04
11718127_at	TK1	0.55	6.00E-04
11721680_a_at	TM2D1	1.41	5.00E-03
11753131_x_at	TM4SF1	2.06	6.00E-05
11725403_at	TM4SF18	2.57	1.00E-03
11728885_x_at	TM6SF1	1.88	4.00E-04
11721006_s_at	TMCO3	1.38	6.00E-03
11729295_at	TMCO7	0.71	3.00E-03
11756630_a_at	TMED10	1.47	1.00E-04
11756147_s_at	TMED2	1.42	3.00E-05
11752760_a_at	TMED7	1.71	7.00E-03
11716729_a_at	TMEM111	1.41	1.00E-03
11758583_s_at	TMEM154	2.08	3.00E-04
11716356_at	TMEM167A	1.28	1.00E-03
11724356_a_at	TMEM177	0.64	1.00E-02
11755308_x_at	TMEM189	1.74	3.00E-06
11727094_at	TMEM19	0.60	6.00E-03
11724739_s_at	TMEM204	1.77	2.00E-03
11727860_at	TMEM209	1.53	3.00E-03
11724122_a_at	TMEM22	1.99	4.00E-05
11732201_a_at	TMEM39A	1.97	4.00E-03
11758119_s_at	TMEM41B	2.69	5.00E-06
11721757_at	TMEM47	1.41	7.00E-03
11758892_a_at	TMEM48	0.57	7.00E-04
11747628_a_at	TMEM49	1.72	7.00E-03
11717522_at	TMEM50B	2.17	1.00E-03
11726747_at	TMEM57	1.59	2.00E-04
11717248_s_at	TMEM66	1.39	4.00E-03
11724642_at	TMEM88	0.25	6.00E-04
11739770_s_at	TMOD2	0.66	7.00E-03
11736447_at	TMPO	0.57	7.00E-03
11720240_at	TMSB15A	0.42	7.00E-05
11747483_a_at	TNFAIP8	1.88	2.00E-03
11732012_x_at	TNFAIP8L3	1.56	8.00E-03
11739342_a_at	TNFRSF10C	0.70	8.00E-03
11740393_at	TNFRSF9	0.33	3.00E-05
11752491_x_at	TOM1	1.48	4.00E-03
11753887_x_at	TOMM5	0.77	4.00E-03
11750094_x_at	TOMM6	0.81	7.00E-03
11718528_a_at	TOR3A	0.55	4.00E-03
11750925_a_at	TOX	0.35	8.00E-05
11723612_a_at	TOX2	1.95	9.00E-03
11749789_a_at	TP53I3	0.62	4.00E-03
11721485_at	TP53RK	0.64	9.00E-03
11732392_x_at	TPI1	0.75	5.00E-03
11761349_x_at	TPM1	0.66	2.00E-03
11754717_a_at	TPP2	1.45	8.00E-03
11721589_a_at	TRAF1	0.59	2.00E-03
11716657_at	TRAF4	0.66	4.00E-03
11757983_s_at	TRAM1	1.72	4.00E-06
11715814_a_at	TRAP1	0.69	5.00E-03
11753721_s_at	TRAPPC2 /// TRAPPC2P1	1.30	8.00E-03
11718280_s_at	TRIAP1	0.73	6.00E-03
11751172_a_at	TRIB3	2.50	8.00E-03
11724023_x_at	TRIM13	0.68	9.00E-03
11725413_x_at	TRIM14	0.38	5.00E-03
11722862_x_at	TRIM2	2.60	1.00E-06
11731526_s_at	TRIP13	0.63	9.00E-03

11759692_at	TRNT1	0.65	9.00E-03
11715413_s_at	TSC22D1	1.67	8.00E-04
11751415_a_at	TSC22D3	2.53	1.00E-05
11723436_a_at	TSGA14	0.68	3.00E-03
11716693_a_at	TSPAN6	0.74	4.00E-03
11719030_a_at	TSPYL2	2.08	7.00E-04
11732142_at	TSR1	0.64	3.00E-04
11764151_s_at	TTC17	1.55	4.00E-03
11760881_x_at	TTF2	0.59	1.00E-04
11743676_a_at	TTLL7	1.79	1.00E-04
11757913_x_at	TUBA1A	0.67	5.00E-04
11744345_x_at	TUBA1B	0.68	4.00E-03
11756670_x_at	TUBA1C	0.67	3.00E-04
11719788_s_at	TUBB2A	0.62	4.00E-05
11750415_x_at	TUBB2C	0.57	4.00E-06
11715791_at	TUBG1	0.67	7.00E-04
11755462_a_at	TUBGCP4	0.73	2.00E-03
11725396_at	TWISTNB	0.63	2.00E-04
11744404_a_at	TXNDC11	1.44	3.00E-03
11757541_x_at	TXNDC17	0.67	6.00E-03
11754067_a_at	TXNDC9	1.28	8.00E-03
11717190_s_at	TXNIP	1.84	1.00E-03
11764006_a_at	TXNL1	1.41	6.00E-03
11715649_s_at	TXNRD1	1.38	4.00E-04
11718058_a_at	TYMS	0.34	2.00E-07
11733346_at	UAP1L1	1.60	6.00E-03
11744653_x_at	UBC	1.32	8.00E-03
11719281_a_at	UBE2I	0.79	1.00E-03
11758226_s_at	UBE2J1	1.88	3.00E-05
11718482_at	UBE2N	0.67	2.00E-03
11729158_at	UBE2T	0.61	9.00E-03
11729304_a_at	UBE3B	0.76	9.00E-03
11727510_at	UBN2	1.78	9.00E-03
11725737_s_at	UBR3	1.40	5.00E-03
11719284_a_at	UBR7	0.70	4.00E-03
11754803_s_at	UCHL1	1.78	5.00E-04
11753842_x_at	UFM1	1.34	6.00E-03
11742021_a_at	UGGT1	1.37	2.00E-03
11723192_a_at	UHRF1	0.29	5.00E-11
11756373_a_at	UMPS	0.71	7.00E-04
11757366_x_at	UNC50	1.41	5.00E-03
11747253_a_at	UNG	0.53	2.00E-03
11752238_x_at	UNKL	1.56	1.00E-03
11734018_at	URB1	0.65	7.00E-05
11746690_a_at	URB2	0.61	2.00E-03
11759627_at	USP15	0.64	3.00E-04
11733911_a_at	UTP15	0.68	8.00E-03
11731664_at	UTP20	0.59	2.00E-05
11743257_x_at	VAPA	0.64	4.00E-03
11719675_a_at	VCAM1	0.28	9.00E-03
11757769_s_at	VCL	0.59	9.00E-05
11721181_a_at	VLDLR	2.50	3.00E-04
11717079_a_at	VPS52	0.74	7.00E-03
11736315_s_at	WBP5	1.51	6.00E-03
11756386_s_at	WDR1	0.78	9.00E-03
11746789_x_at	WDR12	0.72	2.00E-03
11720158_a_at	WDR19	1.47	5.00E-03
11742734_s_at	WDR3	0.57	1.00E-03
11723738_a_at	WDR36	0.67	3.00E-03
11727834_at	WDR43	0.64	8.00E-03
11717308_a_at	WDR75	0.73	4.00E-03
11749299_a_at	WDR77	0.70	3.00E-03

11762563_at	WHSC1	0.74	7.00E-03
11747808_a_at	WIPI1	2.39	4.00E-06
11716831_at	XPOT	2.01	1.00E-05
11720311_a_at	XRCC5	0.73	2.00E-04
11758118_s_at	YARS	1.35	3.00E-03
11724222_at	YIPF4	1.52	5.00E-03
11727717_at	ZBTB42	0.60	2.00E-03
11730940_a_at	ZBTB8A	1.65	4.00E-03
11735241_at	ZC3H12C	2.33	4.00E-03
11749965_x_at	ZCCHC17	1.34	9.00E-03
11744628_x_at	ZCCHC7	1.72	3.00E-03
11756224_s_at	ZDHHC9	1.30	8.00E-03
11723753_a_at	ZFAND2A	3.07	7.00E-05
11750575_a_at	ZFPM2	0.45	7.00E-03
11720334_a_at	ZMIZ1	2.23	3.00E-05
11734397_x_at	ZMYM5	1.51	1.00E-02
11721601_s_at	ZNF238	0.60	4.00E-04
11750300_a_at	ZNF277	1.51	2.00E-03
11740122_a_at	ZNF419	1.64	5.00E-03
11721234_a_at	ZNF75D	1.61	4.00E-03
11739307_s_at	ZNF770	1.38	2.00E-03
11758596_s_at	ZRANB1	1.46	6.00E-03
11740253_a_at	ZWINT	0.48	4.00E-04

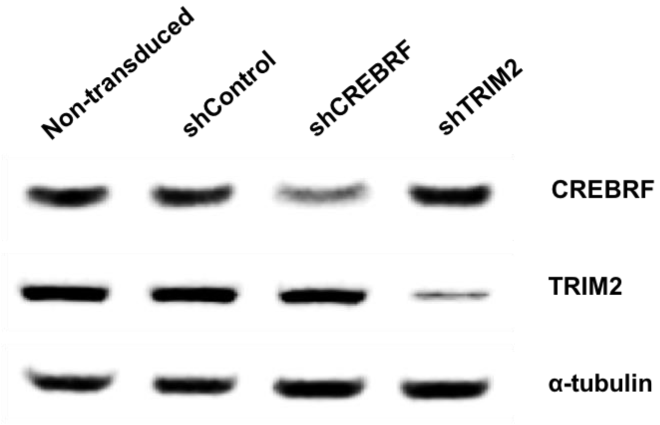

Figure S1. Western blots showing knockdown of CREBRF and TRIM2 protein expression relative to the loading control α-tubulin expression in human coronary artery endothelial cells (HCAECs) following lentiviral delivery of short hairpin RNA directed against *CREBRF* (shCREBRF), *TRIM2* (shTRIM2) and a random control sequence (shControl), compared to non-transduced HCAECs.
